# Oxidation-Cyclisation of Biphenyl Thioethers to Dibenzothiophenium Salts for Ultrarapid ^18^F-Labelling of PET Tracers

**DOI:** 10.3390/ijms232415481

**Published:** 2022-12-07

**Authors:** Fatih Sirindil, Sinead Maher, Michael Schöll, Kerstin Sander, Erik Årstad

**Affiliations:** 1 Centre for Radiopharmaceutical Chemistry, University College London, 5 Gower Place, London WC1E 6BS, UK; 2 Department of Chemistry, University College London, 20 Gordon Street, London WC1H 0AJ, UK; 3 Wallenberg Centre for Molecular and Translational Medicine and the Department of Psychiatry and Neurochemistry, University of Gothenburg, 405 30 Gothenburg, Sweden; 4 Dementia Research Centre, Queen Square Institute of Neurology, University College London, London WC1N 3BG, UK

**Keywords:** fluorine-18, labelling, radiochemistry, positron emission tomography, radiotracer, sulfonium salt, dibenzothiophenium, sulfoxide, UCB-J, AldoView

## Abstract

^18^F-labelled radiotracers are in high demand and play an important role for diagnostic imaging with positron emission tomography (PET). Challenges associated with the synthesis of the labelling precursors and the incorporation of [^18^F]fluoride with practical activity yields at batch scale are the main limitations for the development of new ^18^F-PET tracers. Herein, we report a high-yielding and robust synthetic method to access naked dibenzothiophenium salt precursors of complex PET tracers and their labelling with [^18^F]fluoride. C-S cross-coupling of biphenyl-2-thioacetate with aryl halides followed by sequential oxidation-cyclisation of the corresponding thioethers gives dibenzothiophenium salts in good to excellent yields. Labelling of neutral and electron-deficient substrates with [^18^F]fluoride is ultrarapid and occurs under mild conditions (1 min at 90 °C) with high activity yields. The method enables facile synthesis of complex and sensitive radiotracers, as exemplified by radiofluorination of three clinically relevant PET tracers [^18^F]UCB-J, [^18^F]AldoView and [^18^F]FNDP, and can accelerate the development and clinical translation of new ^18^F-radiopharmaceuticals.

## 1. Introduction

Fluorine-18 labelled tracers are widely used for drug discovery, medical research, and diagnostic imaging with positron emission tomography (PET) [1,2,3]. However, incorporation of fluorine-18 (half-life 110 min) into specific positions of drugs and pharmacological tool compounds is difficult to achieve. For substrates that are amiable to labelling, the need to automate tracer production in accordance with Good Manufacturing Practice (GMP) brings additional challenges that represent a substantial barrier for translation to human studies [4]. Development of more practical and robust labelling strategies can substantially impact on medical research and human health by facilitating translational studies and allowing a broader range of tracers to be used for imaging with PET [5,6]. Herein, we report a concise and high-yielding route for regiospecific incorporation of naked dibenzothiophenium salts as leaving groups for ^18^F-labelling of structurally complex radioligands.

**Scheme 1:**
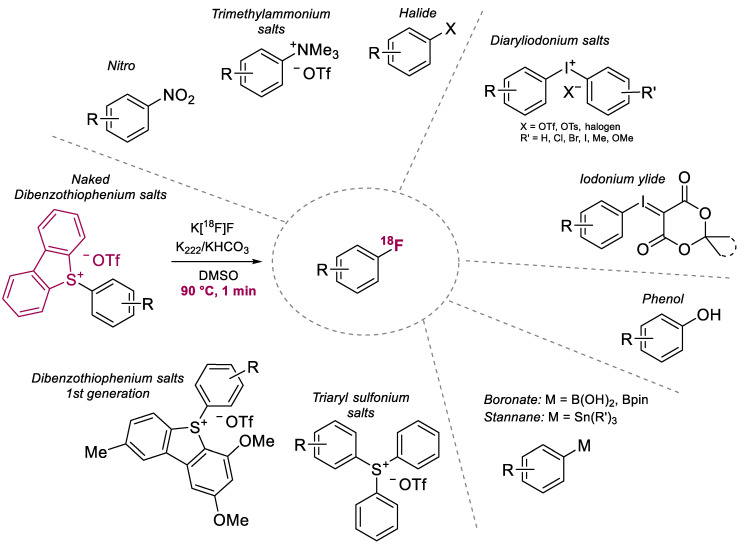
Overview of precursors for radiofluorination of arenes. The precursor used in this work is highlighted.

Due to the common occurrence of aromatic motifs and fluorinated arenes in medicinal chemistry, aromatic [^18^F]fluorination is a particularly appealing strategy for the labelling of PET tracers. Until recently, radiofluorination of arenes was largely confined to nucleophilic aromatic substitution reactions (S_N_Ar) of highly electron-deficient arenes with [^18^F]fluoride, typically using nitro, trimethylammonium, and, to a lesser extent, halides and other leaving groups (Scheme 1) [7,8]. Despite the narrow scope and often low radiochemical yields (RCYs), this remains the predominant method for GMP tracer production [4]. Modern methods for late-stage aromatic [^18^F]fluorination, such as fluorodemetallation, deoxyfluorination, fluorination of onium salt and ylids, and photoredox catalysis has transformed fluorine-18 chemistry (Scheme 1) [9,10]. Despite these advances, only a few of the methods are yet practical and due to their inherent limitations, labelling of compounds with complex functional groups remains fraught with difficulties.

Diaryliodonium salts have been used for labelling of a broad range of PET radiopharmaceuticals for over 20 years, but applications are limited by the low stability of the precursors, poor regioselectivity, and sensitivity to the substitution pattern of the substrate [11]. While the development of spirocyclic iodonium ylides have largely overcome these drawbacks, the precursors for labelling can be challenging to synthesize due to the lack of a generic method to oxidize aryl iodides and the need to protect acidic groups including primary and secondary amides [12]. Among the fluorodemetallation reactions, copper-mediated radiofluorination (CMRF) of arylboronates, stannanes, and iodonium salts has proven particularly versatile, and has been widely adopted in the field [9]. Nevertheless, the method gives unpredictable results for basic amines and heterocycles, and activity yields (AY) are often low and highly variable when the labelling of arylboronates is automated at batch scale [13,14,15]. The use of copper (and stannane precursors) also poses toxicity concerns, which hamper translation to GMP [16,17].

**Scheme 2:**
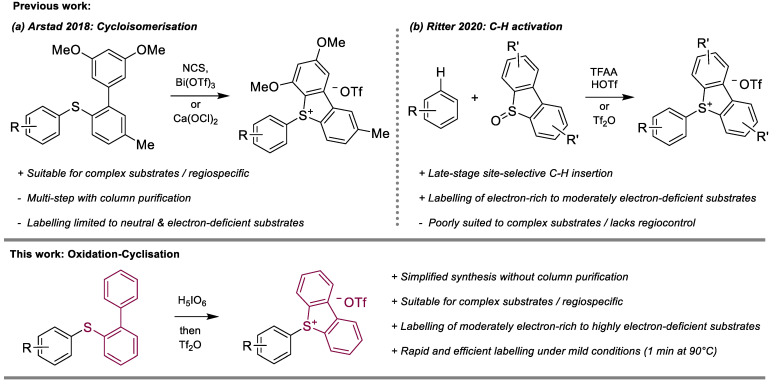
Previous methods for the synthesis of dibenzothiophenium salts and current work.

We, and others, have shown that aryl sulfonium salts allow aromatic [^18^F]fluorination of complex drug-like compounds with predictable RCYs, high process efficiency at batch scale, and that labelling can be automated with minimal need for optimisation [18,19,20,21,22]. As triaryl sulfonium salts can be difficult to access, we developed a ring-closing reaction to form dibenzothiophenium salts from biaryl thioethers and demonstrated their use as leaving groups for labelling of electron-deficient and neutral arenes (Scheme 2a) [19]. However, electron-donating substituents on the biaryl thioether moiety were required to promote cyclisation, which compromised labelling yields and complicated the synthetic route. More recently, the Ritter group reported that activation of dibenzothiophene *S*-oxides with anhydrides to form Pummerer-type intermediates enables site-selective aromatic C–H insertion of dibenzothiophenium salts (Scheme 2b) [22]. This strategy allowed labelling of electron-rich arenes by matching the electron-density of the leaving group to that of the substrate. However, C-H insertion does not allow control over the site of fluorination, which is needed for the majority of PET tracers.

We envisaged that intramolecular cyclisation of biphenyl sulfoxides by anhydride activation would enable regiospecific incorporation of naked dibenzothiophenium salts, which would simplify the synthetic route, broaden the scope, and increase the labelling efficiency. To achieve this, we designed and optimised a reaction sequence involving a C-S cross coupling reaction followed by one-pot sequential oxidation-cyclisation to give the desired precursors for labelling with [^18^F]fluoride.

## 2. Results

**Scheme 3:**
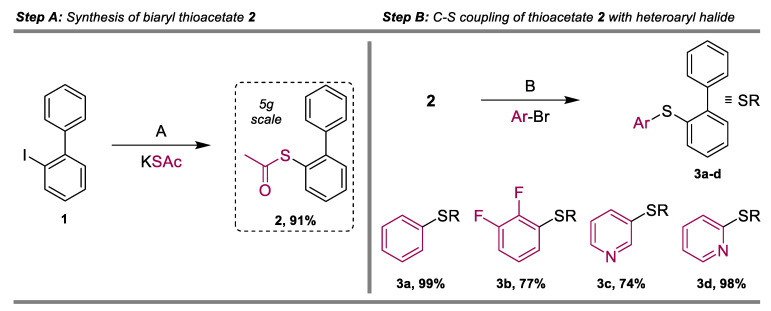
Preparation of the biaryl thioacetate **2** and (hetero)aryl thioether **3a-d**. Reaction conditions for the Step A: **1** (1 equiv.), CuI (20 mol%), 1,10-phenanthroline (40 mol%), KSAc (1.5 equiv.), toluene (0.7 M), 110 °C for 16 h. Reaction conditions for the Step B: **2** (1.2 equiv.), Pd_2_(dba)_3_ (5 mol%), DPEphos (10 mol%), KO*t*Bu (1.2 equiv.), toluene (0.1 M) at 110 °C. Isolated yields are given.

Our first objective was to identify an easily accessible biphenyl thiol surrogate that could be deprotected under mild conditions and allow efficient C-S cross-coupling with aryl halides to form the corresponding thioethers (Scheme 3). The unprotected biphenyl-2-thiol and the corresponding thiobenzoate were difficult to obtain in synthetically useful yields. The thioacetate **2** was particularly appealing as the compound is stable, easy to handle, and odour free, yet can readily be deprotected with potassium *tert*-butoxide (1 equiv.). Inspired by a previously reported method for preparation of aryl thioacetate esters, we optimised conditions for the synthesis of biphenyl thioacetate **2** from commercially available 2-iodobiphenyl (**1**) (91% yield, Scheme 3, Step A) [23,24]. Pleasingly, one-pot in situ deprotection of **2** and palladium catalysed C-S cross-coupling with (hetero)aryl halides, afforded the thioethers **3a-d** in good to excellent yields (74–99%) (Step B) [25].

**Scheme 4:**
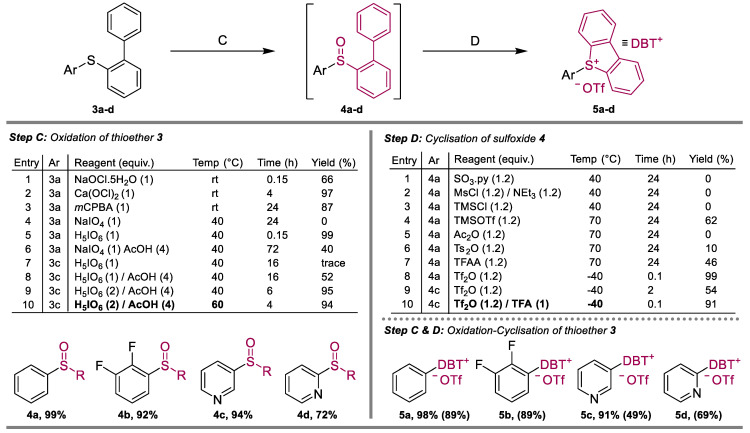
Optimisation of the reaction conditions for one-pot oxidation (Step C)-cyclisation (Step D) and synthesis of dibenzothiophenium salts. Reactions were carried out in MeCN (0.1 M). Step C (optimised): **3** (1 equiv.), H_5_IO_6_ (2 equiv.), AcOH (4 equiv.), 60 °C; 10 min–16 h; isolated yields are given. Step D (optimised): **4** (1 equiv.), TFA (1 equiv.), Tf_2_O 1M in DCM (1.2 equiv.), −40 °C, 5–30 min; Step C & D: isolated yields from thioether **3** are given for the sequential and (one-pot) reactions; for compound **5d**, an excess of Tf_2_O (3 equiv.) and TFA (3 equiv.) was used.

With a practical and efficient method to access biphenyl thioethers in hand, we investigated the oxidation step to the corresponding sulfoxides **4a-d** (Scheme 4, Step C). While several methods have been reported for formation of sulfoxides from biaryl thioethers, most require harsh conditions and are prone to overoxidation to give the corresponding sulfones as side products [26,27,28,29,30]. We aimed to achieve selective oxidation of the biphenyl thioethers in the presence of complex functional groups and basic moieties. Ultimately our goal was to identify conditions for a one-pot oxidation-cyclisation reaction sequence. Using the biaryl thioether **3a** for screening, sodium hypochlorite pentahydrate (NaOCl·5H_2_O) [31] and calcium hypochlorite (Ca(OCl)_2_) gave the sulfoxide **4a** at room temperature in 66% and 97% yield, with 0.15 h and 4 h reaction time, respectively (entry 1–2). *Meta*-chloroperoxybenzoic acid (*m*CPBA) gave 87% yield but required substantially longer reaction time (24 h) (entry 3). Sodium periodate (NaIO_4_) failed to react (entry 4) whereas the acidic congener, periodic acid (H_5_IO_6_), gave **4a** with 99% yield in 10 min at 40 °C (entry 5). While sodium periodate enabled oxidation of **3a** in the presence of excess acetic acid, the reaction was sluggish and low-yielding (entry 6). Of the reagents investigated, we opted for H_5_IO_6_ as it was the most selective and practical to handle. In contrast, hypochlorites are prone to chlorinate electron-rich arenes, while *m*CPBA can oxidize a broad range of functional groups [32,33]. However, when we attempted oxidation of the pyridine thioether **3c** with periodic acid, no reaction occurred (entry 7). It is known that periodic acid is most efficient as an oxidant under moderately acidic conditions as this shifts the dissociation equilibria towards the periodate ion (IO_4_^−^), which is the reactive species [34].

For this reason, we explored the use of acetic acid (pKa 4.76 vs. 3.29 for H_5_IO_6_) to buffer against basic functional groups. Under these conditions, **3c** oxidised to **4c** in 52% yield, but the reaction was sluggish (16 h) (entry 8). Further optimisation revealed the optimal conditions to be excess H_5_IO_6_ (2 equiv.) and acetic acid (4 equiv) with heating to 60 °C, which gave **4c** in 94% yield after 4 h (entry 10). It should be noted that for substrates with multiple basic groups, additional equivalents of acetic acid may be needed for the oxidation to proceed efficiently. To investigate the selectivity for sulfoxide over sulfone formation under the optimised conditions, oxidation of **3c** was monitored by HPLC over a period of 30 h. As <10% of the substrate was converted to the sulfone over this period of time, it is clear that the reaction is highly selective for the sulfoxide even in the presence of excess of periodic acid (for more detail see Supporting Information Figure S1). Using the optimised conditions, thioether **3a-d** were converted to the corresponding sulfoxides **4a-d** (reaction time of 10 min to 16 h) in good to excellent yields (72–99%).

Next, the intramolecular cyclisation of sulfoxides **4a-d** to the corresponding dibenzothiophenium salts **5a-d** was investigated (Scheme 4, Step D). This type of ring closing reaction has previously been reported with alkyl sulfoxide substituted aromatic polymers using trifluoromethanesulfonic acid (HOTf) as solvent or excess (2 equiv.) of trifluoromethanesulfonic anhydride (Tf_2_O) [35,36,37]. However, cyclisation was limited to simple arenes (Me, *t*Bu, OMe, CHO, and CF_3_ substituents) and has not been demonstrated in the presence of complex functional groups. Intermolecular C-H dibenzothiophenylation reactions have been shown for a broader scope of substrates but the reliance on highly acidic conditions (2–4 equiv. of HOTf combined with 3 equiv. of trifluoroacetic anhydride, TFAA), nevertheless limits the tolerance to sensitive functional groups. To find milder cyclisation conditions, we explored several sulfoxide activators using substrate **4a**. Sulfur trioxide pyridine complex (SO_3_.py) (Parikh-Doering type activation) and methanesulfonyl chloride/triethylamine (MsCl/NEt_3_) failed to trigger the cyclisation (entry 1–2). While trimethylsilyl chloride (TMSCl) also failed to react, the triflate counterpart (TMSOTf) provided **5a** in 62% yield after 24 h at 70 °C (entry 2–4). With the exception of acetic anhydride, **4a** underwent cyclisation to **5a** to various degrees in the presence of carboxylic/sulfonic anhydrides. Of the reagents investigated, Tf_2_O proved the most efficient (entry 5–8). Similar to the oxidation step, masking basic groups with acid (TFA) shortened the reaction time and increased the yield as shown for pyridine **4c** (entry 9–10). A combination of Tf_2_O (1.2 equiv.) and TFA (1 equiv.) in acetonitrile at −40 °C was found to be optimal. In case of substrates with multiple basic sites, or less reactive compounds such as 2-pyridines, additional equivalents of TFA may be needed to ensure that the cyclisation proceeds efficiently.

The compatibility of the reagents and solvent for the oxidation and the cyclisation steps allowed the reaction sequence to be carried out in one-pot (Scheme 4, Steps C & D). With the exception of **5c,** the one-pot reaction sequence gave comparable yields to the sequential reactions (e.g., 89% yield for **5a** from **3a** for the one-pot method vs. 98% yield for the sequential reactions). The 2,3-difluorophenyl dibenzothiophenium salt **5b** was obtained with 89% yield in one-pot from **3b** while the pyridines **5c** and **5d** were obtained in 49% (91% sequentially) and 69% yield from **3c** and **3d**, respectively. In contrast to our previously reported ring-closure method, the oxidation-cyclisation route described herein does not give rise to ionic side products, which greatly simplifies purification. Inspired by a procedure to purify sulfonium salts with extraction, we developed a simple trituration procedure that allows the target compounds to be isolated in high purity and good yields without the need for flash chromatography on silica [38]. Thus, dibenzothiophenium salts **5a-d** were obtained with >95% purity as determined by HPLC after trituration of the crude reaction mixtures with diethyl ether.

**Scheme 5:**
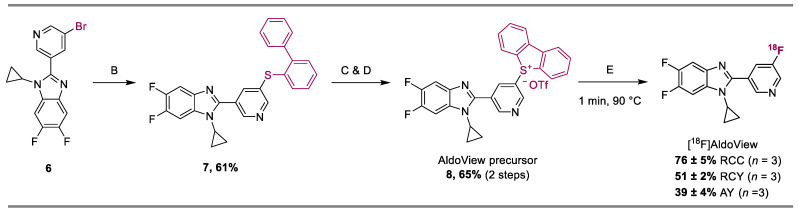
Synthesis of [^18^F]AldoView. Step B: **2** (1.2 equiv.), Pd_2_(dba)_3_ (5 mol%), DPEphos (10 mol%), KO*t*Bu (1.2 equiv.), toluene (0.1 M), 110 °C, 2 h. Step C & D: **7** (1 equiv.), H_5_IO_6_ (2 equiv.), AcOH (4 equiv.), 60 °C, 4 h then TFA (1 equiv.), Tf_2_O 1M in DCM (1.2 equiv.), −40 °C, 5 min. Step E: [^18^F]fluoride, Kryptofix 222/KHCO_3_, DMSO, 90 °C, 1 min.

With the aim to demonstrate practicability of the method, we embarked on the synthesis of precursors for labelling of clinically relevant PET tracers. To maximize the synthetic yields, we adopted a sequential sequence involving C-S coupling, oxidation with extractive workup of the crude sulfoxides, followed by cyclisation. Firstly, we prepared the precursor for labelling of [^18^F]AldoView, a potential biomarker developed by our group for the detection of aldosterone-producing adenomas with PET (Scheme 5) [39]. Coupling of aryl bromide **6** with fragment **2** (61% yield, Step B) followed by oxidation of the thioether **7** and cyclisation of the crude sulfoxide (Step C, D) gave the naked dibenzothiophenium precursor **8** in 65% yield with 97% purity after trituration of the product mixture (Scheme 5). In contrast, using our previously reported ring-closure method the corresponding sulfonium salt precursor of AldoView was obtained in 32% yield and required purification with flash chromatography on silica with two different gradient solvent systems to remove side-products.

**Scheme 6:**
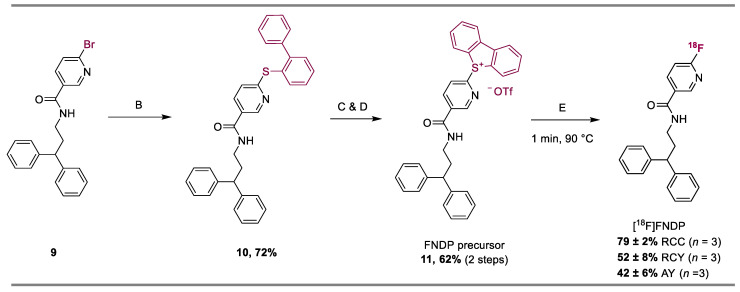
Synthesis of [^18^F]FNDP. Step B: **2** (1.2 equiv.), Pd_2_(dba)_3_ (5 mol%), DPEphos (10 mol%), KO*t*Bu (1.2 equiv.), toluene (0.1 M), 110 °C, 6 h. Step C & D: **10** (1 equiv.), H_5_IO_6_ (2 equiv.), AcOH (4 equiv.), 60 °C, 5 h then TFA (3 equiv.), Tf_2_O 1M in DCM (2 equiv.), −40 °C, 5 min. Step E: [^18^F]fluoride, Kryptofix 222/KHCO_3_, DMSO, 90 °C, 1 min.

**Scheme 7:**
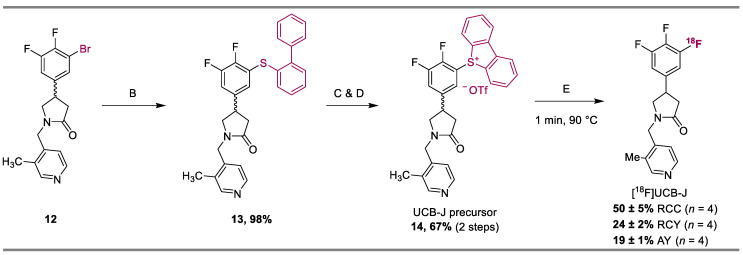
Synthesis of [^18^F]UCBJ. Step B: **2** (1.2 equiv.), Pd_2_(dba)_3_ (5 mol%), DPEphos (10 mol%), KO*t*Bu (1.2 equiv.), toluene (0.1 M), 110 °C, 4 h. Step C & D: **13** (1 equiv.), H_5_IO_6_ (2 equiv.), AcOH (4 equiv.), 60 °C, 20 min then TFA (1 equiv.), Tf_2_O 1M in DCM (1.2 equiv.), −40 °C, 5 min. Step E: [^18^F]fluoride, Kryptofix 222/KHCO_3_, DMSO, 90 °C, 1 min.

Further, we synthetised the precursor **11** for labelling of [^18^F]FNDP, a promising PET tracer for imaging of soluble epoxidase hydrolase (sEH) (Scheme 6) [40,41,42]. Coupling of aryl bromide **9** with fragment **2** gave thioether **10** in 72% yield. Subsequent oxidation and cyclisation using excess of Tf_2_O (2 equiv.) and TFA (3 equiv.) afforded the labelling precursor **11** in 62% yield (95% purity). Finally, we prepared precursor **14** for labelling of [^18^F]UCB-J, a biomarker of synaptic density (Scheme 7) [43,44,45,46,47]. Coupling of aryl bromide **12** with fragment **2** gave the thioether **13** in near quantitative yield (98%, Step B). Subsequent oxidation and cyclisation provided the dibenzothiophenium salt **14** with 67% yield with 98% purity (Scheme 7). Dibenzothiophenium salts are generally non-hygroscopic crystalline solids and stable at ambient temperature [19]. The precursors **8**, **11**, and **14** remained stable following storage for over one year in the cold (+2–8 °C).

With the dibenzothiophenium precursors **8**, **11**, and **14** in hand we investigated ^18^F-labelling of the respective PET tracers. Our initial focus was to label [^18^F]UCB-J as alternative strategies have failed to provide the ^18^F-labelled version of this tracer in practical RCYs [48,49]. Using our previously reported conditions for labelling of dibenzothiophenium salts (2 mg of **14**, [^18^F]KF/K_222_, 0.5 mL DMSO, 100 °C, 15 min), afforded [^18^F]UCB-J in 13% RCY. However, labelling was accompanied by formation of large amounts of non-radioactive UCB-J, presumably due to competing nucleophilic substitution of the aryl fluoride substituents in the precursor. Screening of the reaction parameters revealed that 2 mg **14** in DMSO at 90 °C for 1 min gave the best compromise between RCY and formation of non-radioactive UCB-J. Under these conditions, [^18^F]UCB-J was obtained with 24 ± 2% RCY/19 ± 1% AY (*n* = 4) with a radiochemical purity >99% and a total of 66 ± 7 μg of the cold compound at batch scale (Scheme 7) (Supporting Information Figures S10–S14). Intrigued by the mild conditions and short reaction time, we subjected the precursors **8** and **11** to the same labelling conditions. This provided [^18^F]AldoView in 51 ± 2% RCY/39 ± 4% AY (*n* = 3) with a molar activity of 4.1 ± 0.4 GBq/μmol (*n* = 2, starting from 300 MBq) (Scheme 5) (Supporting Information Figures S2–S5) and [^18^F]FNDP in 52 ± 8% RCY/42 ± 6% AY (*n* = 3) with a molar activity of 71 ± 4 GBq/μmol (*n* = 3, starting from 400 MBq) (Scheme 6) (Supporting Information Figures S6–S9). The molar activity reflects the ratio of ^18^F to ^19^F fluoride in the labelling reaction. As the main sources of ^19^F comes from the synthesis process, including contamination from the QMA cartridge used for trapping and release of [^18^F]fluoride, K_2_CO_3_, and the solvent, the molar activity is highly dependent on the activity level used for labelling [50,51]. In this study, labelling was limited to low activity (300–400 MBq), manual reactions, and hence, the exemplar tracers were obtained with low molar activity. However, we and others have previously shown that dibenzothiophenium salts allow automated labelling of tracers with high molar activity at scale [20,21,22]. While the total amount of cold UCB-J formed during labelling was substantially higher than for other tracers, scale up to higher activity levels may nevertheless allow production of [^18^F]UCB-J with comparable molar activity to that of [^11^C]UCB-J [52]. We are currently preparing the *R* isomer of precursor **14** with the view to develop an automated method and investigate its suitability for production of [^18^F]UCB-J for clinical studies.

## 3. Discussion

As compared to our previously reported ring-closing synthesis of dibenzothiophenium salts, the oxidation-cyclisation route reported herein offers important practical advantages. Firstly, the key thiol coupling fragment **2** is readily accessible via a one-step scalable reaction from commercially available 2-iodobiphenyl. The ease of deprotection of the thioacetate **2** facilitates C-S coupling as the thiol can be liberated without the need for excess base, and the resulting acetate byproduct can readily be removed during purification. We have previously shown that coupling of aryl halides (bromide and iodide) with biaryl thiophenols tolerates a broad range of functional groups [19]. The subsequent oxidation-cyclisation reaction sequence is highly robust and enables facile synthesis of labelling precursors of complex ligands that otherwise are difficult to access, such as UCB-J [48,49] (We were unable to prepare the precursor using the method reported in reference [19]), electron-deficient substrates as exemplified by FNDP, and 3-pyridines which are challenging to label with alternative strategies [53]. While further studies are needed to determine the scope of the oxidation and cyclisation steps, it is worth noting that periodic acid typically requires a catalyst to mediate oxidation of other functional groups, with the exception of anilines, unprotected amino acids, and 1,2-diols [54]. Due to the high chemical stability of biaryl thioethers and sulfoxides, the C-S coupling and oxidation steps can (in most cases) be carried out at any stage of the precursor synthesis. We anticipate that the main limitation of the synthetic strategy is the need for triflic anhydride to mediate cyclisation, which precludes applications to substrates with acid-labile functional groups, including BOC protected amines.

Finally, the high reactivity of the leaving group allows ultrarapid labelling of neutral and electron-withdrawing substrates under mild conditions (90 °C for 1 min). This not only confers practical benefits and maximizes the activity yields, but also allows labelling of heat-sensitive compounds. In comparison, labelling of [^18^F]AldoView with our previously reported method required heating to 110 °C for 15 min, which gave 42 ± 8% RCY (vs. 51 ± 2% RCY in this study) [39]. For [^18^F]UCB-J, hypervalent iodonium salts and alternative modern leaving groups required heating 170 °C for 20 min to react with [^18^F]fluoride, which caused racemisation and only afforded ~5% RCC/1–2% RCY (vs. 19 ± 1% AY in this study) [48,49]. Labelling of [^18^F]FNDP using the bromide precursor **9** required heating 160 °C for 12 min to give 14 ± 7% AY (vs. 42 ± 6% AY in this study) [40,41,42]. However, it should be noted that for labelling of moderately electron-rich substrates, such as substituted *ortho* and *para* phenol ethers [22], higher temperatures and longer reaction times may be required.

## 4. Materials and Methods

### 4.1. Procedure for Step A

To a solution of 2-iodobiphenyl **1** (5.0 g, 17.8 mmol, 1 equiv.) in dry toluene (0.7 M, 26 mL) under argon, copper (I) iodide (680 mg, 3.6 mmol, 20 mol%), 1,10-phenanthroline (1.3 g, 7.1 mmol, 40 mol%) and potassium thioacetate (3.0 g, 26.8 mmol, 1.5 equiv.) were added sequentially, and the resulting mixture was stirred at 110 °C for 16 h. The reaction was cooled to reach room temperature, filtered over a pad of Celite^®^ with EtOAc and concentrated in vacuo. The crude product was purified by flash column chromatography (cyclohexane/EtOAc gradient 0% to 5%) on silica gel to afford the desired product **2** as an orange oil in 91% yield (3.7 g).

### 4.2. Procedure for Step B

To a flame-dried tube with screw cap (15 mL) under argon, Pd_2_(dba)_3_ (5 mol%), DPEphos (10 mol%), aryl halide (1 equiv.), S-([1,1′-biphenyl]-2-yl)ethanethioate **2** (1.2 equiv.), and dry toluene (0.1 M) were added sequentially. The resulting mixture was degassed by bubbling argon through the mixture for 5 min. Then, potassium *tert*-butoxide (1.2 equiv.) was added and the tube was sealed. The reaction mixture was then heated at 110 °C and monitored by TLC until completion (1–16 h). After cooling to room temperature, the reaction mixture was concentrated in vacuo. The crude product was purified by flash column chromatography (cyclohexane/EtOAc) on silica gel to afford the desired product **3a-d**.

### 4.3. Procedure for Step C

To a stirring solution of thioether **3a-d** (1 equiv.) in anhydrous acetonitrile (0.1 M), glacial acetic acid was added (4 equiv.), followed by orthoperiodic acid (2 equiv.) (Sigma Aldrich, Gillingham, Dorset, UK, catalogue number: P7875-100G). The resulting reaction mixture was heated to 60 °C, stirring until the starting material was fully consumed, as determined by TLC (10 min–16 h). The reaction was cooled to room temperature and quenched with saturated aqueous NaHCO_3_ (2 mL) and diluted with dichloromethane (5 mL). The layers were separated, and the aqueous layer was extracted twice with DCM (5 mL). The combined organic layers were dried over MgSO_4_, filtered, and concentrated in vacuo. The crude product was purified by flash column chromatography (cyclohexane/EtOAc) on silica gel to afford the desired product **4a-d**.

### 4.4. Procedure for Step D

Sulfoxide **4a-d** (1 equiv.) was dissolved in anhydrous acetonitrile (0.1 M) under argon and trifluoroacetic acid (1 equiv.) was added. The solution was cooled to −40 °C (acetone/dry ice bath). A solution of trifluoromethanesulfonic anhydride 1M in dichloromethane (1.2 equiv.) (Sigma Aldrich, Gillingham, Dorset, UK, catalogue number: 704083-25 mL) was then added under stirring and allowed to react for 5 to 30 min. The reaction was allowed to reach room temperature, quenched with saturated aqueous NaHCO_3_ (2 mL) and diluted with dichloromethane (5 mL). The layers were separated, and the aqueous layer was extracted twice with DCM (5 mL). The combined organic layers were dried over MgSO_4_, filtered, and concentrated in vacuo. The crude product was purified by trituration with diethyl ether (10 mL, x3) to afford the desired product **5a-d**.

### 4.5. One-Pot Procedure for Steps C & D

The sulfoxides **4a-d** were prepared according to the general procedure for Step C from thioether **3**. Once the starting material was consumed the reaction mixture was cooled to −40 °C (acetone/dry ice bath). Trifluoroacetic acid (1 equiv.) was added, followed by a solution trifluoromethanesulfonic anhydride 1M in dichloromethane (1.2 equiv.) (Sigma Aldrich, catalogue number: 704083-25 mL), and the resulting solution was stirred for 5 to 30 min. The reaction was allowed to reach room temperature, was quenched with saturated aqueous NaHCO_3_ (2 mL) and diluted with dichloromethane (5 mL). The layers were separated and the aqueous layer was extracted twice with dichloromethane (5 mL). The combined organic layers were dried over MgSO_4_, filtered, and concentrated in vacuo. Purification by trituration with diethyl ether (10 mL, ×3) afforded the desired dibenzothiophenium salt **5a-d**.

### 4.6. Procedure for Step E

[^18^F]fluoride in ^18^O-water was trapped on a Sep-Pak^®^ QMA light cartridge (Waters, Wilmslow, UK, catalogue number: WAT023525) and released with a solution Kryptofix 222 (30 mM) and potassium bicarbonate (30 mM) in acetonitrile/water (85%/15% v/v; 0.5 mL). The solvent was removed by heating at 90 °C under a stream of nitrogen. [^18^F]fluoride was dried by azeotropic distillation with acetonitrile (2 × 0.5 mL; 90 °C) and the reaction vial was subsequently capped. The vial was preheated at 90 °C for 1 min. The corresponding sulfonium salt (2 mg) in anhydrous DMSO (0.5 mL) was added to the reaction vial and stirred at 90 °C for 1 min. Then, cold water (1 mL) was immediately added to the reaction. The radiochemical product was purified using semi-preparative HPLC.

More detailed procedures, experimental procedures for the synthesis of ^18^F radiotracers, all characterization data, and original spectra can be found in the Supporting Information.

## 5. Conclusions

We have developed a method for regiospecific incorporation of naked dibenzothiophenium salts as leaving groups for aromatic [^18^F]fluorination. The three-step reaction sequence, involving C-S coupling, S-oxidation, and intramolecular cyclisation, is high-yielding and the target sulfonium salts can be purified using a simple trituration procedure without the need for column chromatography on silica. The method allowed ^18^F-labelling of three clinically relevant PET tracers in good activity yields at batch scale within 1 min under mild conditions and with low precursor load. The robustness of the synthetic route and high labelling efficiency can accelerate the development and clinical translation of complex ^18^F-radiopharmaceuticals.

## Data Availability

The authors declare that all the data supporting the findings of this study are available within this article.

## Supplementary Materials

The supporting information can be downloaded at: https://www.mdpi.com/article/10.3390/ijms232415481/s1.
