# Supplementary material for: Oxidation-Cyclisation of Biphenyl Thioethers to Dibenzothiophenium Salts for Ultrarapid 18F-Labelling of PET Tracers
Source: Int J Mol Sci. 2022 Dec 7;23(24):15481. doi: 10.3390/ijms232415481 (PMC9779140; doi:10.3390/ijms232415481)
Supplement: Supplementary file 1 [file ijms-23-15481-s001.zip › ijms-2028595-supplementary.pdf]

Supporting Information

Oxidation-Cyclisation of Biphenyl Thioethers to Dibenzothiophenium Salts for Ultrarapid ^{18}F-Labelling of PET Tracers

Fatih Sirindil[1,2], Sinead Maher[1,2], Michael Schöll[3,4], Kerstin Sander[1,2] and Erik Årstad[1,2]*

[1] Centre for Radiopharmaceutical Chemistry, University College London, London WC1E 6BS, UK

[2] Department of Chemistry, University College London, London WC1H 0AJ, UK

[3] Wallenberg Centre for Molecular and Translational Medicine and the Department of Psychiatry and Neurochemistry, University of Gothenburg, 405 30 Gothenburg, Sweden

[4] Dementia Research Centre, Queen Square Institute of Neurology, University College London, London WC1N 3BG, UKS

GENERAL INFORMATION 1

PROCEDURE FOR STEP A : SYNTHESIS OF BIARYL THIOACETATE 2 2

GENERAL PROCEDURE FOR STEP B : SYNTHESIS OF ARYL/HETEROARYL THIOETHER 3A-D 3

GENERAL PROCEDURE FOR STEP C : SYNTHESIS OF SULFOXIDE 4A-D 5

OXIDATION REACTION MONITORING BY HPLC 8

GENERAL PROCEDURE FOR STEP D : SYNTHESIS OF DIBENZOTHIOPHENIUM SALT 5A-D 9

SYNTHESIS OF DIBENZOTHIOPHENIUM PRECURSOR: ALDOVIEW, FNDP AND UCBJ 12

[^{18}F] RADIOLABELING OF DIBENZOTHIOPHENIUM SALTS 19

NMR SPECTRA : ^{1}H, ^{13}C AND ^{18}F 33

General Information

Proton (^1H NMR), Carbon (^{13}C NMR) and Fluorine (^{19}F NMR) nuclear magnetic resonance spectra were recorded on 300, 400 or 500 MHz instruments. The chemical shifts are given in part per million (ppm) on the delta scale. The solvent peak was used as reference values. For ^1H NMR: $CDCl_3$ = 7.26 ppm, CD_3CN = 1.94 ppm. For ^{13}C NMR: $CDCl_3$= 77.16 ppm, CD_3CN = 1.32 ppm. Data are presented as followed; chemical shift, multiplicity (s = singlet, d = doublet, t = triplet, q = quartet, quint = quintet, m = multiplet, b = broad), coupling constants (*J* in Hz) and integration and carbons with the same chemical shift as follows: chemical shift (x carbons). Infrared spectra were recorded neat. Wavelengths of maximum absorbance (ν_{max}) are quoted in wave numbers (cm^{-1}). High resolution mass spectra (HRMS) data were recorded on a microTOF spectrometer equipped with an orthogonal electrospray interface (ESI). The parent ions $[M]^+$, $[M+H]^+$, $[M+K]^+$ $[M+Li]^+$ or $[M+Na]^+$ are quoted. Melting points were taken on a Gallenkamp heating block and are uncorrected. Analytical thin layer chromatography (TLC) was carried out on silica gel 60 F_{254} plates with visualization by ultraviolet light and/or chemical solutions. Flash column chromatography was carried out using SiO_2 60 (40−63 µm) and the procedures included the subsequent evaporation of solvents *in vacuo*. All other commercially available reagents were used as received (Sigma-Aldrich, Acros Organics or Fluorochem). All extractive procedures were performed using technical grade solvents, and all aqueous solutions were saturated unless details are given. All air- and moisture-sensitive reactions were carried out in flame-dried glassware under an argon atmosphere.

Procedure for Step A : Synthesis of biaryl thioacetate 2

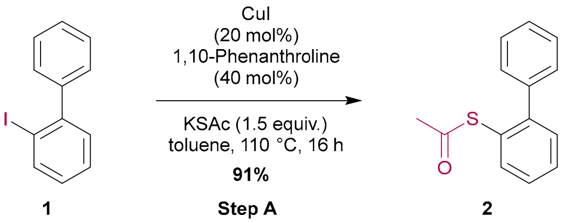

To a solution of 2-iodobiphenyl **1** (5.0 g, 17.8 mmol, 1 equiv.) in dry toluene (0.7 M, 26 mL) under argon, copper (I) iodide (680 mg, 3.6 mmol, 20 mol%), 1,10-phenanthroline (1.3 g, 7.1 mmol, 40 mol%) and potassium thioacetate (3.0 g, 26.8 mmol, 1.5 equiv.) were added sequentially, and the resulting mixture was stirred at 110 °C for 16 hours. The reaction mixture was cooled at room temperature, filtered over a pad of Celite® with EtOAc and concentrated *in vacuo*. The crude product was purified by flash column chromatography (cyclohexane/EtOAc gradient 0% to 5%) on silica gel to afford the desired product **2** as an orange oil in 91% yield (3.7 g).

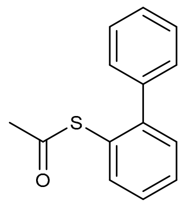

Chemical Formula: $C_{14}H_{12}OS$
Exact Mass: 228.0609
Molecular Weight: 228.3090

S-([1,1'-biphenyl]-2-yl) ethanethioate (2):

Orange oil; **TLC** R_f 0.34 (cyclohexane/EtOAc 4 %);

IR (neat) ν_{max} 420, 441, 470, 520, 555, 611, 682, 698, 734, 748, 773, 846, 871, 916, 947, 1008, 1039, 1074, 1107, 1259, 1352, 1423, 1446, 1463, 1496, 1560, 1587, 1600, 1701;

^{1}H NMR ($CDCl_3$, 500 MHz) ***δ*** 7.56 – 7.53 (m, 1H), 7.50 – 7.46 (m, 1H), 7.43 – 7.35 (m, 5H), 7.31 – 7.27 (m, 2H), 2.26 (s, 3H);

^{13}C NMR ($CDCl_3$, 126 MHz) ***δ*** 193.85, 146.41, 140.85, 136.67, 130.98, 129.99 (x2), 129.36, 128.15, 127.93, 127.56 (x2), 126.53, 30.21;

HR-MS 229.0681 ($C_{14}H_{12}OS+H^+$) calcd 229.0682.

General Procedure for Step B : Synthesis of aryl/heteroaryl thioether 3a-d

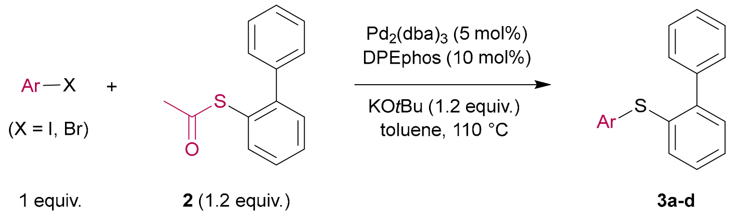

To a flame-dried tube with screw cap (15 mL) under argon, $Pd_2(dba)_3$ (5 mol%), DPEphos (10 mol%), aryl halide (1 equiv.), S-([1,1'-biphenyl]-2-yl) ethanethioate **2** (1.2 equiv.) and dry toluene (0.1 M) were added sequentially. The resulting mixture was degassed by bubbling argon through the mixture for 5 minutes. Then potassium *tert*-butoxide (1.2 equiv.) was added and the tube was sealed. The reaction mixture was then heated at 110 °C and monitored by TLC until completion (1-16 hours). After cooling to room temperature, the reaction mixture was concentrated *in vacuo*. The crude product was purified by flash column chromatography (cyclohexane/EtOAc) on silica gel to afford the desired product **3a-d**.

Caution: In order to avoid over pressure in the sealed tube which potentially could lead to explosion, make sure suitable/compatible tubes are used and the volume of solvent used for the reactions maintained below 1.5 mL. Higher scale reactions can be carried out in a round bottom flask equipped with a condenser.

Note: Using excess potassium *tert*-butoxide can cause formation of side products and lower the yields.

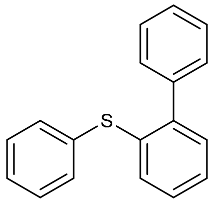

Chemical Formula: $C_{18}H_{14}S$
Exact Mass: 262.0816
Molecular Weight: 262.3700

[1,1'-biphenyl]-2-yl(phenyl)sulfane (3a): Prepared following the general procedure for step B (1 hour reaction time) in 99% yield (635 mg) from 500 mg of iodobenzene. A 100 mL round bottom flask equipped with a condenser was used.

Colorless oil; **TLC** R_f 0.48 (cyclohexane/EtOAc 1 %);

^{1}H NMR ($CDCl_3$, 500 MHz) δ 7.49 – 7.42 (m, 4H), 7.42 – 7.35 (m, 2H), 7.35 – 7.24 (m, 8H);

^{13}C NMR ($CDCl_3$, 126 MHz) δ 143.21, 140.83, 135.81, 135.22, 132.08, 131.41, 130.76, 129.58, 129.35, 128.24, 128.16, 127.64, 127.34, 126.99.

Consistent with literature data.[19]

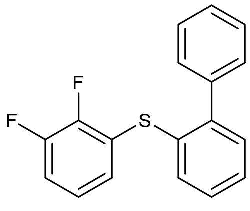

Chemical Formula: $C_{18}H_{12}F_2S$
Exact Mass: 298.0628
Molecular Weight: 298.3508

[1,1'-biphenyl]-2-yl(2,3-difluorophenyl)sulfane (3b): Prepared following the general procedure for step B (4 hours reaction time) in 77% yield (60 mg) from 50 mg of 1-bromo-2,3-difluorobenzene.

Colorless oil; **TLC** *R*f 0.32 (cyclohexane);

IR (neat) $\mathbf{v_{max}}$ 433, 468, 499, 530, 553, 572, 613, 678, 698, 713, 746, 769, 817, 842, 898, 997, 1008, 1037, 1056, 1074, 1128, 1147, 1182, 1222, 1269, 1425, 1456, 1475, 1496, 1585, 1608;

^{1}H NMR ($CDCl_3$, 500 MHz) δ 7.44 – 7.33 (m, 7H), 7.33 – 7.27 (m, 2H), 7.09 – 7.01 (m, 1H), 6.95 (tdd, *J* = 8.1, 4.8, 1.5 Hz, 1H), 6.89 (ddt, *J* = 7.8, 5.9, 1.6 Hz, 1H);

^{13}C NMR ($CDCl_3$, 126 MHz) δ 151.82 (d, *J* = 13.5 Hz), 150.47 (d, *J* = 13.2 Hz), 149.83 (d, *J* = 13.4 Hz), 148.49 (d, *J* = 13.2 Hz), 143.95, 140.43, 132.36 (d, *J* = 1.3 Hz), 132.03, 130.97, 129.38 (x2), 128.35, 128.19 (d, *J* = 3.4 Hz), 128.10 (x2), 127.85, 127.67, 125.83 (d, *J* = 14.4 Hz), 124.22 (dd, *J* = 7.1, 4.9 Hz), 116.45 (d, *J* = 17.5 Hz);

^{19}F NMR ($CDCl_3$, 282 MHz) δ -133.08 (d, *J* = 22.1 Hz), -136.75 (d, *J* = 22.1 Hz);

HR-MS 299.0695 ($C_{18}H_{12}F_2S+H^+$) calcd 299.0701.

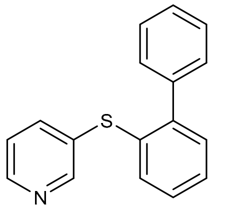

Chemical Formula: $C_{17}H_{13}NS$
Exact Mass: 263.0769
Molecular Weight: 263.3580

3-([1,1'-biphenyl]-2-ylthio)pyridine (3c): Prepared following the general procedure for step B (16 hours reaction time) in 74% yield (48 mg) from 50 mg of 3-iodopyridine.

Orange oil; **TLC** *R*f 0.19 (cyclohexane/EtOAc 10 %);

IR (neat) $\mathbf{v_{max}}$ 420, 445, 511, 538, 553, 615, 678, 698, 746, 771, 794, 844, 873, 914, 1016, 1037, 1089, 1105, 1120, 1159, 1188, 1224, 1259, 1319, 1404, 1446, 1462, 1496, 1558, 1585, 1735;

^{1}H NMR ($CDCl_3$, 500 MHz) δ 8.45 – 8.37 (m, 2H), 7.46 (ddd, *J* = 8.0, 2.4, 1.6 Hz, 1H), 7.42 – 7.24 (m, 9H), 7.13 (ddd, *J* = 8.0, 4.8, 0.9 Hz, 1H);

^{13}C NMR ($CDCl_3$, 126 MHz) δ 151.64, 147.82, 143.99, 140.39, 138.54, 133.51, 133.12, 132.31, 130.99, 129.37 (x2), 128.38, 128.08 (x2), 127.89, 127.66, 123.83;

HR-MS 264.0840 ($C_{17}H_{13}NS+H^+$) calcd 264.0841.

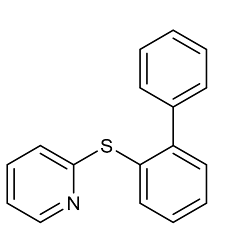

Chemical Formula: $C_{17}H_{13}NS$
Exact Mass: 263.0769
Molecular Weight: 263.3580

2-([1,1'-biphenyl]-2-ylthio)pyridine (3d): Prepared following the general procedure for step B (16 hours reaction time) in 98% yield (166 mg) from 100 mg of 2-bromopyridine.

Yellow oil; **TLC** *R*f 0.26 (cyclohexane/EtOAc 5 %);

IR (neat) $\mathbf{v_{max}}$ 420, 449, 472, 509, 534, 553, 613, 636, 682, 698, 721, 748, 842, 875, 914, 952, 983, 1008, 1039, 1074, 1116, 1143, 1180, 1257, 1278, 1415, 1446, 1463, 1496, 1558, 1573, 1598;

^{1}H NMR ($CDCl_3$, 500 MHz) δ 8.35 (dd, *J* = 5.1, 1.8 Hz, 1H), 7.67 (dd, *J* = 7.7, 1.3 Hz, 1H), 7.50 – 7.42 (m, 2H), 7.41 – 7.25 (m, 7H), 6.93 (dd, *J* = 7.4, 4.9 Hz, 1H), 6.84 (d, *J* = 8.0 Hz, 1H);

^{13}C NMR ($CDCl_3$, 126 MHz) δ 161.26, 149.65, 146.33, 140.81, 136.55, 136.32, 131.28, 130.09, 129.43 (x2), 129.32, 128.49, 127.88 (x2), 127.46, 122.14, 119.92;

HR-MS 264.0833 ($C_{17}H_{13}NS+H^+$) calcd 264.0841.

General Procedure for Step C : Synthesis of sulfoxide 4a-d

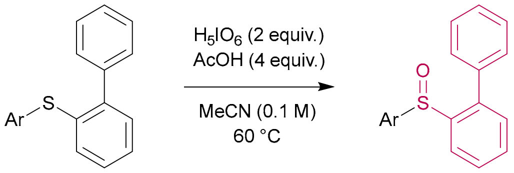

To a stirring solution of thioether **3a-d** (1 equiv.) in anhydrous acetonitrile (0.1 M) was added glacial acetic acid (4 equiv.) followed by orthoperiodic acid (2 equiv.) (Sigma Aldrich, catalogue number : P7875-100G). The resulting reaction mixture was heated to 60 °C with stirring until the starting material was fully consumed as determined by TLC (10 min – 16 hours). The reaction was cooled to room temperature and quenched with saturated aqueous $NaHCO_3$ (2 mL) and diluted with dichloromethane (5 mL). The layers were separated, and the aqueous layer was extracted twice with DCM (5 mL). The combined organic layers were dried over $MgSO_4$, filtered and concentrated *in vacuo*. The crude product was purified by flash column chromatography (cyclohexane/EtOAc) on silica gel to afford the desired product **4a-d**.

Note: It is important to stop the reaction shortly after the starting material has been consumed (monitoring by TLC) to avoid overoxidation of the sulfoxide to the sulfone.

Caution: Periodic acid may cause explosion if heated to dryness. Precaution must be taken to avoid solvent evaporation during the reaction. Solution of periodic acid in dimethyl sulfoxide (DMSO) have been reported to explode.

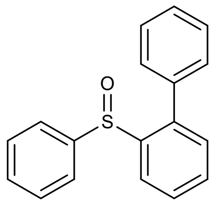

Chemical Formula: $C_{18}H_{14}OS$
Exact Mass: 278.0765
Molecular Weight: 278.3690

2-(phenylsulfinyl)-1,1'-biphenyl (4a): Prepared following the general procedure for step C (10 minutes reaction time) in 99% yield (21 mg) from 20 mg of **3a**.

Colorless oil; **TLC** R_f 0.21 (cyclohexane/EtOAc 30 %);

IR (neat) ν_{max} 423, 482, 516, 546, 581, 613, 687, 703, 747, 762, 777, 843, 856, 888, 922, 965, 996, 1006, 1031, 1041, 1079, 1126, 1161, 1235, 1306, 1326, 1428, 1443, 1460, 1579;

^{1}H NMR ($CDCl_3$, 500 MHz) δ 8.24 – 8.02 (m, 1H), 7.58 (td, J = 7.6, 1.4 Hz, 1H), 7.49 (td, J = 7.4, 1.5 Hz, 1H), 7.44 – 7.36 (m, 3H), 7.35 – 7.15 (m, 6H), 7.11 – 7.04 (m, 2H);

^{13}C NMR ($CDCl_3$, 126 MHz) δ 144.97, 143.63, 140.87, 138.18, 130.94, 130.87, 130.52, 129.74 (x2), 128.93 (x2), 128.69, 128.61 (x2), 128.27, 125.69 (x2), 124.21,

HR-MS 279.0838 ($C_{18}H_{14}OS+H^+$) calcd 279.0838.

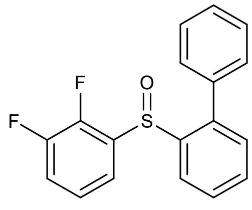

Chemical Formula: $C_{18}H_{12}F_2OS$
Exact Mass: 314.0577
Molecular Weight: 314.3498

2-((2,3-difluorophenyl)sulfinyl)-1,1'-biphenyl (4b): Prepared following the general procedure for step C (10 minutes reaction time) in 92% yield (19.4 mg) from 20 mg of **3b**.

Colorless oil; **TLC** *R*f 0.18 (cyclohexane/EtOAc 20 %);

IR (neat) ν_{max} 414, 470, 507, 540, 549, 576, 626, 698, 709, 754, 773, 785, 812, 881, 916, 1006, 1029, 1076, 1147, 1180, 1219, 1269, 1446, 1456, 1477, 1591;

^{1}H NMR ($CDCl_3$, 500 MHz) *δ* 8.00 – 7.91 (m, 1H), 7.67 – 7.49 (m, 2H), 7.45 – 7.35 (m, 3H), 7.35 – 7.31 (m, 1H), 7.30 – 7.26 (m, 2H), 7.25 – 7.22 (m, 1H), 7.20 – 7.08 (m, 2H);

^{13}C NMR ($CDCl_3$, 126 MHz) *δ* 151.00 (d, *J* = 14.4 Hz), 148.98 (d, *J* = 14.3 Hz), 148.18 (d, *J* = 16.1 Hz), 146.15 (d, *J* = 16.1 Hz), 142.10, 141.84, 137.88, 134.93 (dd, *J* = 8.9, 1.4 Hz), 129.37 (x2), 128.76, 128.57 (x2), 128.35, 125.96, 124.90 (t, *J* = 5.3 Hz), 121.99 (dd, *J* = 2.9, 1.6 Hz), 120.10 (t, *J* = 9.1 Hz);

^{19}F NMR ($CDCl_3$, 282 MHz) *δ* -136.82;

HR-MS 315.0647 ($C_{18}H_{12}F_2OS+H^+$) calcd 315.0650.

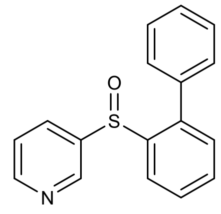

Chemical Formula: $C_{17}H_{13}NOS$
Exact Mass: 279.0718
Molecular Weight: 279.3570

3-([1,1'-biphenyl]-2-ylsulfinyl)pyridine (4c): Prepared following the general procedure for step C (4 hours reaction time) in 94% yield (19.9 mg) from 20 mg of **3c**.

Orange oil; **TLC** *R*f 0.22 (cyclohexane/EtOAc 30 %);

IR (neat) ν_{max} 486, 499, 518, 547, 617, 704, 732, 758, 775, 802, 1006, 1014, 1031, 1049, 1072, 1120, 1159, 1190, 1209, 1222, 1321, 1409, 1462, 1570, 1730;

^{1}H NMR (CD_3CN, 500 MHz) *δ* 8.50 (s, 1H), 8.13 (dd, *J* = 7.9, 1.4 Hz, 1H), 7.99 (s, 1H), 7.67 (td, *J* = 7.7, 1.3 Hz, 1H), 7.59 (td, *J* = 7.5, 1.3 Hz, 1H), 7.42 (qd, *J* = 7.9, 3.9 Hz, 4H), 7.31 (dd, *J* = 7.5, 1.3 Hz, 1H), 7.29 – 7.20 (m, 3H);

^{13}C NMR (CD_3CN, 126 MHz) *δ* 151.52, 146.35, 142.75, 141.43, 140.13, 137.38, 132.60, 130.94, 130.28, 129.07 (x2), 128.44, 128.39 (x2), 128.12, 123.90, 123.33;

HR-MS 280.0791 ($C_{17}H_{13}NOS+H^+$) calcd 280.0791.

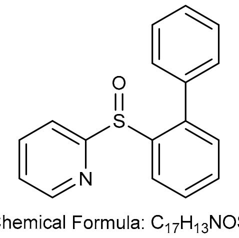

Chemical Formula: $C_{17}H_{13}NOS$
Exact Mass: 279.0718
Molecular Weight: 279.3570

2-([1,1'-biphenyl]-2-ylsulfinyl)pyridine (4d): Prepared following the general procedure for step C (16 hours reaction time) in 72% yield (15.2 mg) from 20 mg of **3d**.

Yellow oil; **TLC** *R*f 0.19 (cyclohexane/EtOAc 30 %);

IR (neat) ν_{max} 435, 466, 489, 524, 538, 555, 613, 677, 707, 729, 738, 758, 773, 871, 900, 933, 991, 1006, 1029, 1083, 1132, 1151, 1217, 1365, 1423, 1448, 1465, 1560, 1573, 1589, 1739;

^{1}H NMR (CD_3CN, 500 MHz) *δ* 8.46 (dd, *J* = 4.6, 1.6 Hz, 1H), 7.94 (tt, *J* = 7.8, 1.3 Hz, 1H), 7.85 (dd, *J* = 7.9, 1.1 Hz, 1H), 7.71 (dd, *J* = 7.9, 1.4 Hz, 1H), 7.64 – 7.58 (m, 2H), 7.58 – 7.49 (m, 2H), 7.48 – 7.43 (m, 3H), 7.43 – 7.39 (m, 1H), 7.39 – 7.31 (m, 1H);

^{13}C NMR (CD_3CN, 126 MHz) *δ* 165.49, 149.48, 143.10, 142.07, 138.02, 137.92, 131.05, 130.13, 129.92 (x2), 128.45, 127.87 (x2), 127.77, 125.87, 124.54, 119.51;

HR-MS 280.0791 ($C_{17}H_{13}NOS+H^+$) calcd 280.0791.

Oxidation Reaction Monitoring by HPLC

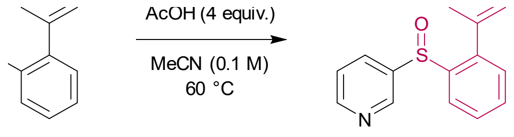

4c

Reaction was performed according to the general procedure C and monitored by HPLC. An aliquot (10µL) was taken at different time point (0.5h-72h) and injected into HPLC (each sample composition = 10µL aliquot + 90µL MeCN + 100µL H_2O). The conversions were determined using the UV (254nm) chromatograms.

Column: Agilent Eclipse Plus C-18 column (5 µm; 150 × 4.6 mm) at room temperature

Methods: Flow rate : 1.80 mL/min

Time (min)	H_2O + 0.1% TFA (%)	MeOH + 0.1% TFA (%)
0.00	90	10
15.00	30	70
17.00	10	90

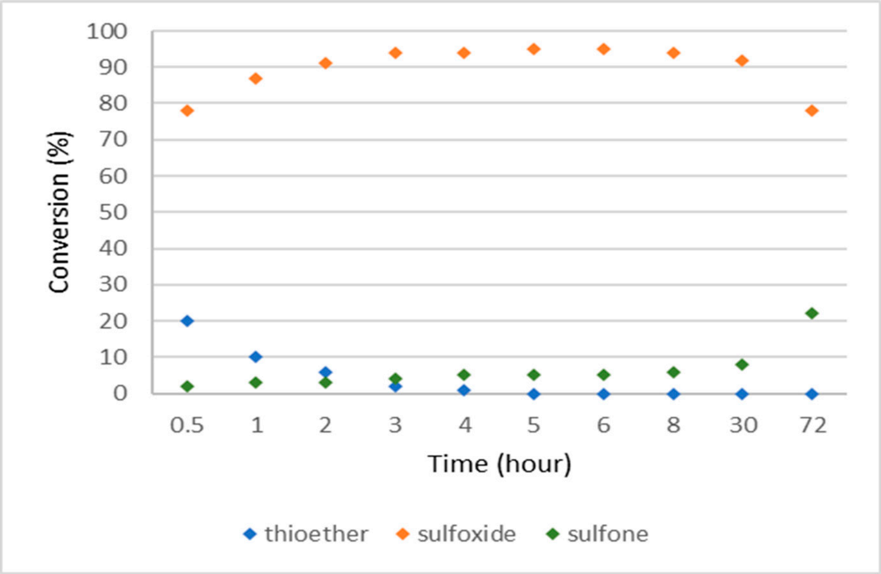

Figure 1 : Oxidation reaction monitoring by HPLC.

General Procedure for Step D : Synthesis of dibenzothiophenium salt 5a-d

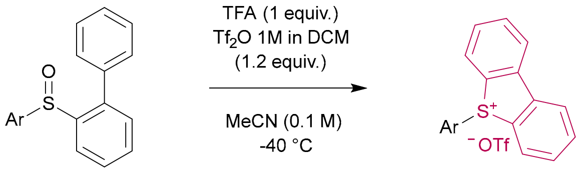

Sulfoxide **4a-d** (1 equiv.) was dissolved in anhydrous acetonitrile (0.1 M) under argon and trifluoroacetic acid (1 equiv.) was added. The solution was cooled to -40 °C (acetone/dry ice bath). A solution of trifluoromethanesulfonic anhydride 1M in dichloromethane (1.2 equiv.) (Sigma Aldrich, catalogue number : 704083-25 mL) was then added under stirring and allowed to react for 5 min. The reaction was allowed to reach room temperature and quenched with saturated aqueous $NaHCO_3$ (2 mL) and diluted with dichloromethane (5 mL). The layers were separated, and the aqueous layer was extracted twice with DCM (5 mL). The combined organic layers were dried over $MgSO_4$, filtered and concentrated *in vacuo*. The crude product was purified by trituration with diethyl ether (10 mL, x3) to afford the desired product **5a-d**.

One-pot synthesis (step C & D) of dibenzothiophenium salt 5a-d from thioether 3a-d:

The sulfoxides **4a-d** were prepared according to the general procedure for **step C**. Once the starting material was consumed (monitored by TLC) the reaction mixture was cooled at -40 °C (acetone/dry ice bath). Trifluoroacetic acid (1 equiv.) was added, followed by a solution of trifluoromethanesulfonic anhydride 1M in dichloromethane (1.2 equiv.) (Sigma Aldrich, catalogue number : 704083-25 mL), and the resulting solution was stirred for 5 minutes. The reaction was allowed to reach room temperature and was quenched with saturated aqueous $NaHCO_3$ (2 mL) and diluted with dichloromethane (5 mL). The layers were separated and the aqueous layer was extracted twice with dichloromethane (5 mL). The combined organic layers were dried over $MgSO_4$, filtered and concentrated *in vacuo*. Purification by trituration with diethyl ether (10 mL, x3) afforded the desired dibenzothiophenium salt **5a-d**.

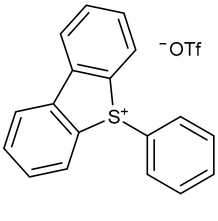

Chemical Formula: $C_{19}H_{13}F_3O_3S_2$
Exact Mass: 261.0732 ($C_{18}H_{13}S^+$)
Molecular Weight: 410.4252

5-phenyl-5H-dibenzo[b,d]thiophen-5-ium trifluoromethanesulfonate (5a): Prepared following the one-pot procedure in 89% yield (27.9 mg) from 20 mg of **3a**.

White solid; **mp** 186-188 °C; **TLC** *R*f 0.21 (dichloromethane/MeOH 5 %);

IR (neat) ν_{max} 420, 433, 487, 499, 516, 526, 553, 570, 613, 632, 680, 707, 732, 750, 763, 960, 974, 995, 1028, 1049, 1064, 1095, 1151, 1170, 1219, 1259, 1294, 1446, 1452, 1475;

^{1}H NMR (CD_3CN, 500 MHz) *δ* 8.39 – 8.32 (m, 2H), 8.08 (dt, *J* = 8.2, 0.8 Hz, 2H), 7.95 (td, *J* = 7.7, 1.0 Hz, 2H), 7.78 – 7.69 (m, 3H), 7.62 – 7.56 (m, 4H);

^{13}C NMR (CD_3CN, 126 MHz) *δ* 139.19, 134.89, 134.22 (x2), 131.64, 131.42 (x2), 131.35 (x2), 130.17 (x2), 127.66 (x2), 126.57, 124.31 (x2), 122.19, 119.63;

^{19}F NMR ($CDCl_3$, 282 MHz) *δ* -79.33;

HR-MS 261.0732 ($C_{18}H_{13}S^+$) calcd 261.0732.

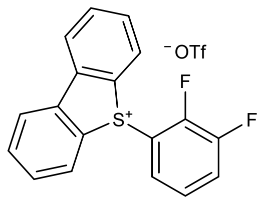

Chemical Formula: $C_{19}H_{11}F_5O_3S_2$
Exact Mass: 297.0544 ($C_{18}H_{11}F_2S^+$)
Molecular Weight: 446.4060

5-(2,3-difluorophenyl)-5H-dibenzo[b,d]thiophen-5-ium trifluoromethanesulfonate (5b): Prepared following the one-pot procedure in 89% yield (40.1 mg) from 30 mg of **3b**.

White solid; **mp** 169-170 °C; **TLC** *R*f 0.24 (dichloromethane/MeOH 5 %);

IR (neat) ν_{max} 422, 451, 464, 486, 514, 559, 570, 613, 636, 702, 738, 759, 777, 817, 887, 960, 999, 1031, 1145, 1186, 1224, 1257, 1311, 1365, 1452, 1496, 1598, 1735;

^{1}H NMR (CD_3CN, 500 MHz) *δ* 8.39 (dd, *J* = 7.8, 1.2 Hz, 2H), 8.19 (d, *J* = 8.1 Hz, 2H), 8.01 (td, *J* = 7.7, 1.0 Hz, 2H), 7.79 (td, *J* = 7.9, 1.2 Hz, 2H), 7.72 (dtd, *J* = 9.8, 8.2, 1.5 Hz, 1H), 7.39 (tdd, *J* = 8.4, 4.5, 1.7 Hz, 1H), 7.24 (ddt, *J* = 8.5, 5.6, 1.6 Hz, 1H);

^{13}C NMR (CD_3CN, 126 MHz) *δ* 152.22 (d, *J* = 13.3 Hz), 151.73 (d, *J* = 18.5 Hz), 149.68 (d, *J* = 13.3 Hz), 149.13 (d, *J* = 18.5 Hz), 139.92, 134.94 (x2), 131.83 (x2), 129.51, 128.41 (x2), 127.44 (dd, *J* = 6.9, 4.6 Hz), 126.82 (dd, *J* = 3.7, 1.6 Hz), 125.13 (dd, *J* = 16.9, 1.8 Hz), 124.76 (x2), 122.75, 119.56, 115.65 (d, *J* = 10.9 Hz);

^{19}F NMR ($CDCl_3$, 282 MHz) *δ* -79.25, -133.75 (d, *J* = 19.8 Hz), -133.96 (d, *J* = 19.9 Hz);

HR-MS 297.0544 ($C_{18}H_{11}F_2S^+$) calcd 297.0544.

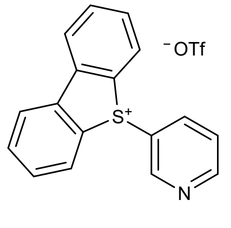

Chemical Formula: $C_{18}H_{12}F_3NO_3S_2$
Exact Mass: 262.0685 ($C_{17}H_{12}NS^+$)
Molecular Weight: 411.4132

5-(pyridin-3-yl)-5H-dibenzo[b,d]thiophen-5-ium trifluoromethanesulfonate (5c): Prepared following the one-pot procedure in 49% yield (18.9 mg) from 25 mg of **3c**. 3-4 drops of dichloromethane was added to diethyl ether for trituration.

Orange solid; **mp** 175-177 °C (decomposes); **TLC** *R*f 0.17 (dichloromethane/MeOH 5 %);

IR (neat) ν_{max} 424, 489, 514, 528, 572, 599, 615, 634, 696, 705, 756, 773, 800, 902, 1010, 1026, 1103, 1157, 1224, 1257, 1365, 1419, 1452, 1529, 1573, 1602, 1658, 1739;

^{1}H NMR (CD_3CN, 500 MHz) *δ* 8.99 (d, *J* = 2.4 Hz, 1H), 8.88 (dd, *J* = 4.7, 1.4 Hz, 1H), 8.39 (dd, *J* = 7.9, 1.2 Hz, 2H), 8.23 – 8.09 (m, 2H), 8.00 (td, *J* = 7.7, 1.0 Hz, 2H), 7.77 (ddd, *J* = 8.5, 7.6, 1.2 Hz, 2H), 7.71 (ddd, *J* = 8.4, 2.5, 1.4 Hz, 1H), 7.52 (dd, *J* = 8.4, 4.7 Hz, 1H);

^{13}C NMR (CD_3CN, 126 MHz) *δ* 155.42, 151.35, 139.68, 137.55, 134.73 (x2), 131.85 (x2), 131.27, 128.21 (x2), 126.34, 125.44, 124.77 (x2), 122.76, 119.57;

^{19}F NMR ($CDCl_3$, 282 MHz) *δ* -79.27;

HR-MS 262.0681 ($C_{17}H_{12}NS^+$) calcd 262.0685.

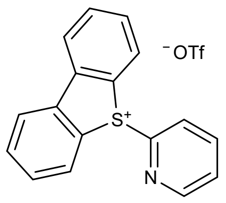

Chemical Formula: $C_{18}H_{12}F_3NO_3S_2$
Exact Mass: 262.0685 ($C_{17}H_{12}NS^+$)
Molecular Weight: 411.4132

5-(pyridin-2-yl)-5H-dibenzo[b,d]thiophen-5-ium trifluoromethanesulfonate (5d): Prepared following the one-pot procedure in 69% yield (33.4 mg) from 33 mg of **3d**. 3 equivalents of Tf_2O and TFA were used to reach full conversion in 30 minutes. 3-4 drops of dichloromethane was added to diethyl ether for trituration.

White solid; **mp** 139-143 °C; **TLC** R_f 0.18 (DCM/MeOH 5 %);

IR (neat) ν_{max} 417, 427, 454, 485, 514, 529, 571, 610, 631, 707, 728, 765, 792, 891, 967, 989, 1025, 1076, 1145, 1219, 1253, 1285, 1338, 1423, 1447, 1464, 1481, 1557, 1577, 1654;

^{1}H NMR (CD_3CN, 500 MHz) *δ* 8.55 – 8.48 (m, 1H), 8.34 (d, *J* = 7.8 Hz, 2H), 8.23 (d, *J* = 8.1 Hz, 2H), 8.16 – 8.04 (m, 2H), 7.96 (t, *J* = 7.7 Hz, 2H), 7.76 (t, *J* = 7.8 Hz, 2H), 7.67 (ddd, *J* = 6.7, 4.7, 1.9 Hz, 1H);

^{13}C NMR (CD_3CN, 126 MHz) *δ* 152.95, 149.48, 141.22, 140.69, 134.84 (x2), 131.75 (x2), 130.13, 129.15, 128.88 (x2), 127.72, 124.73 (x2), 123.06, 119.87;

^{19}F NMR ($CDCl_3$, 282 MHz) *δ* -79.24;

HR-MS 262.0685 ($C_{17}H_{12}NS^+$) calcd 262.0685.

Synthesis of Dibenzothiophenium Precursor: AldoView, FNDP and UCBJ

1) Synthesis of AldoView labelling precursors 8

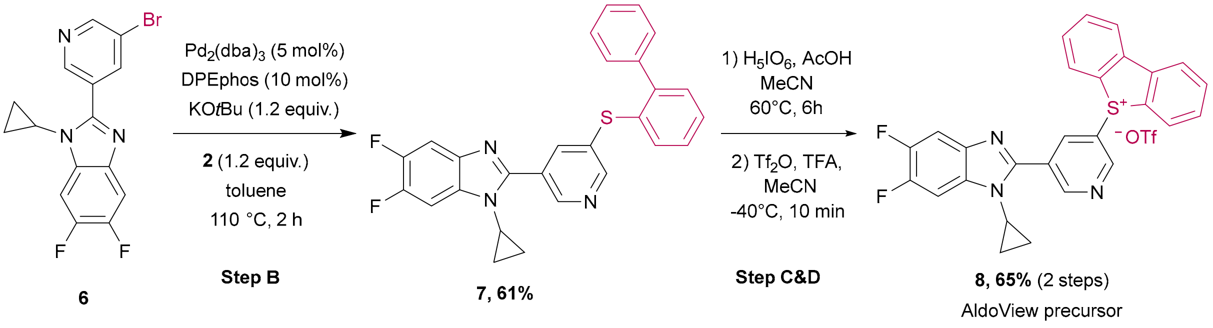

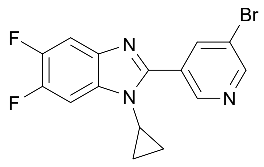

Chemical Formula: $C_{15}H_{10}BrF_2N_3$
Exact Mass: 349.0026
Molecular Weight: 350.1668

2-(5-bromopyridin-3-yl)-1-cyclopropyl-5,6-difluoro-1H-benzo[d]imidazole (6): Compound **6** was synthesized according a previously reported procedure.[39]

^{1}H NMR (CDCl₃, 500 MHz) ***δ*** 9.13 (d, *J* = 1.9 Hz, 1H), 8.80 (d, *J* = 2.2 Hz, 1H), 8.45 (t, *J* = 2.1 Hz, 1H), 7.57 (dd, *J* = 10.2, 7.3 Hz, 1H), 7.41 (dd, *J* = 9.7, 7.0 Hz, 1H), 3.57 (tt, *J* = 6.9, 3.8 Hz, 1H), 1.29 – 1.21 (m, 2H), 0.85 – 0.78 (m, 2H);

^{13}C NMR (CDCl₃, 126 MHz) ***δ*** 151.8, 151.0, 150.1-149.5 (1C), 147.9-147.6 (1C), 147.5, 138.9, 138.1-137.9 (1C), 132.7-132.7 (1C), 128.0, 120.8, 107.8-107.6 (1C), 99.3-99.1 (1C), 26.6, 9.2 (2C);

^{19}F NMR (CDCl₃, 282 MHz) ***δ*** -139.4 & -139.5 (1F), -142.2 & -142.3 (1F);

HR-MS 350.0109 ($C_{15}H_{10}BrF_2N_3+H^+$) calcd 350.0104.

Consistent with literature data.[39]

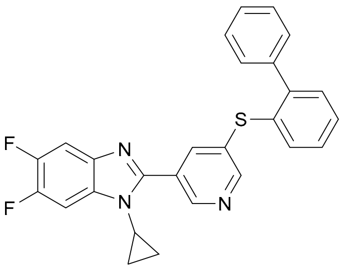

Chemical Formula: $C_{27}H_{19}F_2N_3S$
Exact Mass: 455.1268
Molecular Weight: 455.5268

2-(5-([1,1'-biphenyl]-2-ylthio)pyridin-3-yl)-1-cyclopropyl-5,6-difluoro-1H-benzo[d]imidazole (7): Prepared following the general procedure for step B (2 hours reaction time) in 61% yield (158 mg) from 157 mg of **6**.

Yellow solid; **mp** 94 °C; **TLC** *R*f 0.19 (CHCl₃ 100 %);

IR (neat) ν_{max} 433, 447, 491, 505, 543, 578, 621, 653, 698, 717, 748, 794, 813, 839, 875, 900, 914, 925, 964, 1008, 1037, 1072, 1097, 1126, 1159, 1193, 1222, 1278, 1311, 1332, 1355, 1392, 1408, 1442, 1463, 1500, 1550, 1585, 1602, 1687, 3061;

^{1}H NMR (CDCl₃, 500 MHz) ***δ*** 8.93 (d, *J* = 2.0 Hz, 1H), 8.38 (d, *J* = 2.1 Hz, 1H), 7.96 (t, *J* = 2.1 Hz, 1H), 7.53 (dd, *J* = 10.3, 7.3 Hz, 1H), 7.47 (dd, *J* = 7.7, 1.3 Hz, 1H), 7.42 – 7.28 (m, 9H), 3.38 (tt, *J* = 7.1, 3.8 Hz, 1H), 1.08 (td, *J* = 7.2, 5.7 Hz, 2H), 0.73 – 0.66 (m, 2H);

^{13}C NMR (CDCl$_3$, 126 MHz) *δ* 151.84 (d, *J* = 3.4 Hz), 151.35, 149.44 (dd, *J* = 33.1, 15.3 Hz), 147.51 (dd, *J* = 30.6, 15.2 Hz), 147.35, 144.78, 140.30, 137.76 (d, *J* = 10.5 Hz), 137.65, 134.18, 133.58, 132.55 (d, *J* = 10.6 Hz), 131.89, 131.29, 129.39 (x2), 128.69, 128.56, 128.12 (x2), 127.74, 126.57, 107.48 (d, *J* = 19.5 Hz), 99.05 (d, *J* = 23.3 Hz), 26.45, 8.98 (x2);

^{19}F NMR (CDCl$_3$, 282 MHz) *δ* -139.26 (d, *J* = 20.6 Hz), -141.96 (d, *J* = 20.9 Hz);

HR-MS 456.1340 ($C_{27}H_{19}F_2N_3S+H^+$) calcd 456.1341.

Procedure for the synthesis of AldoView precursor 8 from 7

Periodic acid (40 mg, 0.17 mmol, 2 equiv.) was added to a stirring solution of **7** (40 mg, 0.087 mmol, 1 equiv.) and AcOH (20 µL, 0.35 mmol, 4 equiv.) in anhydrous acetonitrile (1.1 mL, 0.1M). The resulting reaction mixture was stirred at 60 °C for 6 hours (monitored by TLC). The reaction was quenched with saturated aqueous $NaHCO_3$ (2 mL) and diluted with dichloromethane (5 mL). The layers were separated and the aqueous layer was extracted twice with dichloromethane (5 mL). The combined organic layers were dried over $MgSO_4$, filtered and concentrated *in vacuo*. The resulting crude product was dissolved with anhydrous acetonitrile (1.1 mL, 0.1M) and trifluoroacetic acid (14 µL, 0.17 mmol, 2 equiv.) was added. The reaction mixture was cooled at -40 °C (acetone/dry ice bath) and a solution of trifluoromethanesulfonic anhydride 1M in dichloromethane (110 µL, 0.10 mmol, 1.2 equiv.) was added and the resulting solution was stirred for 5 minutes. The reaction was allowed to reach room temperature and was quenched with saturated aqueous $NaHCO_3$ (2 mL) and diluted with dichloromethane (10 mL). The layers were separated and the aqueous layer was extracted twice with dichloromethane (10 mL). The combined organic layers were dried over $MgSO_4$, filtered and concentrated *in vacuo*. The crude product was purified by trituration with diethyl ether (10 mL, x3, 3-4 drops of dichloromethane was added to diethyl ether for each trituration) to afford the desired product **8** as an white solid with yield of 65% (34.7 mg) and 97% purity assessed by HPLC.

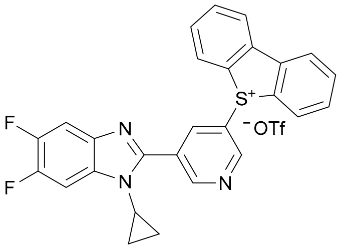

Chemical Formula: $C_{28}H_{18}F_5N_3O_3S_2$
Exact Mass: 454.1184 ($C_{27}H_{18}F_2N_3S^+$)
Molecular Weight: 603.5820

5-(5-(1-cyclopropyl-5,6-difluoro-1H-benzo[d]imidazol-2-yl)pyridin-3-yl)-5H-dibenzo[b,d]thiophen-5-ium trifluoromethanesulfonate (8):

White solid; **mp** 190 °C (decomposes); **TLC** R_f 0.19 (DCM/MeOH 6 %);

IR (neat) ν_{max} 416, 426, 478, 514, 542, 570, 634, 655, 694, 717, 744, 761, 810, 837, 856, 898, 956, 1018, 1041, 1066, 1128, 1157, 1192, 1222, 1263, 1317, 1338, 1373, 1394, 1415, 1440, 1469, 1558, 1598, 1631, 1660, 2166, 2850, 2922, 3037;

^{1}H NMR (CD$_3$CN, 500 MHz) *δ* 9.39 (d, *J* = 1.8 Hz, 1H), 9.13 (d, *J* = 2.3 Hz, 1H), 8.37 (d, *J* = 7.9 Hz, 2H), 8.22 (d, *J* = 8.1 Hz, 2H), 8.06 (t, *J* = 2.1 Hz, 1H), 7.97 (t, *J* = 7.6 Hz, 2H), 7.76 (t, *J* = 7.8 Hz, 2H), 7.52 (ddd, *J* = 10.8, 7.3, 5.5 Hz, 2H), 3.29 (tt, *J* = 7.1, 3.8 Hz, 1H), 0.80 (td, *J* = 7.2, 5.2 Hz, 2H), 0.60 – 0.51 (m, 2H);

^{13}C NMR (CD$_3$CN, 126 MHz) *δ* 155.92, 152.58, 151.10, 150.12 (dd, *J* = 41.9, 15.4 Hz), 148.21 (dd, *J* = 39.1, 15.5 Hz), 140.73, 138.62 (d, *J* = 11.1 Hz), 137.66, 135.91 (x2), 133.99 (d, *J* = 11.2 Hz), 132.94 (x2), 132.11, 129.86, 129.48 (x2), 126.26, 125.89 (x2), 123.34, 120.79, 107.92 (d, *J* = 19.9 Hz), 100.68 (d, *J* = 23.2 Hz), 26.99, 9.00 (x2);

^{19}F NMR (CD$_3$CN, 282 MHz) *δ* -79.37, -142.01 (d, *J* = 20.1 Hz), -144.93 (d, *J* = 20.0 Hz);

HR-MS 454.1181 ($C_{27}H_{18}F_2N_3S^+$) calcd 454.1184

2) Synthesis of FNDP labelling precursors 11

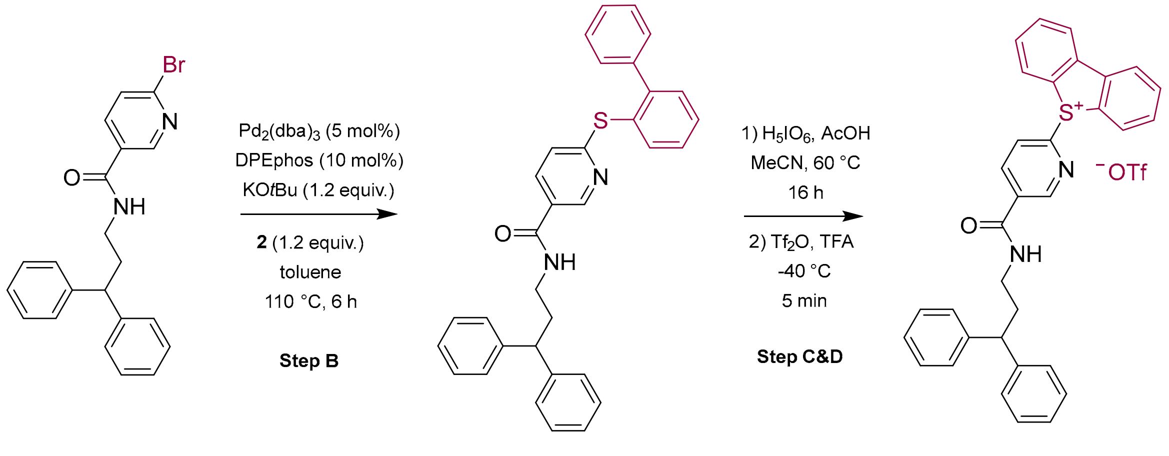

9 **10, 72%** **11, 62%** (2 steps) FNDP precursor

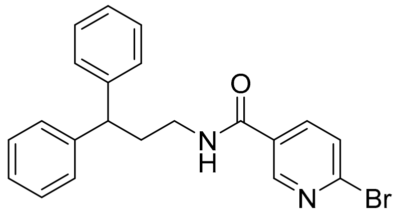

Chemical Formula: $C_{21}H_{19}BrN_2O$
Exact Mass: 394.0681
Molecular Weight: 395.3000

6-bromo-*N*-(3,3-diphenylpropyl)nicotinamide (9): Compound **9** was synthesized according a previously reported procedure.[42]

^{1}H NMR (CDCl₃, 400 MHz,) ***δ*** 8.39 (d, *J* = 2.5 Hz, 1H), 7.73 (dt, *J* = 8.2, 2.6 Hz, 1H), 7.50 (dd, *J* = 8.3, 2.3 Hz, 1H), 7.35 – 7.11 (m, 8H), 5.91 (s, 1H), 3.99 (td, *J* = 7.7, 2.3 Hz, 1H), 3.50 (qd, *J* = 6.7, 2.2 Hz, 2H), 2.41 (qd, *J* = 7.0, 2.3 Hz, 2H);

^{13}C NMR (CDCl₃, 126 MHz) ***δ*** 164.36, 148.10, 145.15, 144.31 (x2), 137.52, 129.41, 128.94 (x4), 128.20, 127.80 (x4), 126.78 (x2), 50.14, 39.76, 35.09;

HR-MS 395.0750 ($C_{21}H_{19}BrN_2O+H^+$) calcd 395.0753.

Consistent with literature data.[42]

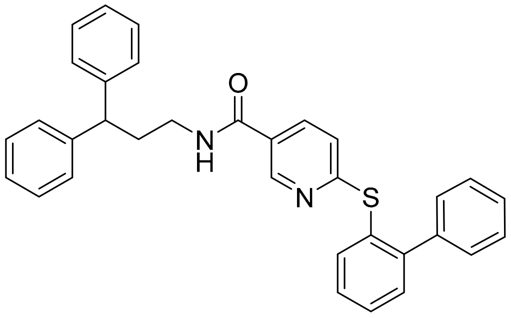

Chemical Formula: $C_{33}H_{28}N_2OS$
Exact Mass: 500.1922
Molecular Weight: 500.6600

6-([1,1'-biphenyl]-2-ylthio)-*N*-(3,3-diphenylpropyl)nicotinamide (10): Prepared following the general procedure for step B (20 hours reaction time) in 72% yield (36.3 mg) from 40 mg of **9**.

Yellow oil. **TLC** *R*f 0.38 (cyclohexane/EtOAc 10 %);

IR (neat) $\mathbf{\nu_{max}}$ 438, 469, 696, 749, 901, 1029, 1104, 1237,1261, 1312, 1356, 1448, 1491, 1536, 1580, 1631, 2848, 2918, 3021, 3053, 3307;

^{1}H NMR (CDCl₃, 500 MHz) ***δ*** 8.44 (d, *J* = 2.2 Hz, 1H), 7.67 (ddd, *J* = 10.4, 8.1, 1.8 Hz, 2H), 7.56 – 7.37 (m, 3H), 7.35 – 7.22 (m, 13H), 7.17 (ddt, *J* = 7.4, 6.1, 1.9 Hz, 2H), 6.77 (d, *J* = 8.3 Hz, 1H),

6.01 (d, *J* = 5.9 Hz, 1H), 3.99 (t, *J* = 7.7 Hz, 1H), 3.44 (q, *J* = 6.5 Hz, 2H), 2.38 (q, *J* = 7.2 Hz, 2H);

^{13}C NMR ($CDCl_3$, 126 MHz) *δ* 165.26, 165.11, 147.17, 146.97, 144.31 (x2), 140.49, 136.99, 135.72, 131.54, 130.17, 129.32 (x2), 128.84 (x4), 128.75, 128.37, 127.97 (x2), 127.82 (x4), 127.64, 126.65 (x2), 126.11, 120.75, 49.78, 39.41, 35.16;

HR-MS 501.1988 ($C_{33}H_{28}N_2OS+H^+$) calcd 501.1995.

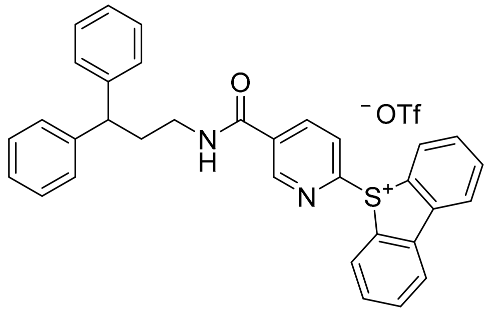

Chemical Formula: $C_{34}H_{27}F_3N_2O_4S_2$
Exact Mass: 499.1839 ($C_{33}H_{27}N_2OS^+$)
Molecular Weight: 648.7152

5-(5-((3,3-diphenylpropyl)carbamoyl)pyridin-2-yl)-5H-dibenzo[b,d]thiophen-5-ium trifluoromethanesulfonate (11): Prepared following the procedure for the synthesis of **8**. 5 hours reaction time for the oxidation step. Obtained in 62% yield (14 mg) from 17 mg of **10**. 2 equivalents of Tf_2O and 3 equivalents of TFA were used.

White solid; **mp** 190-200 °C; **TLC** R_f 0.19 (DCM/MeOH 5 %);

IR (neat) ν_{max} 514, 571, 636, 700, 754, 1028, 1142, 1255, 1447, 1545, 1579, 1647, 1737, 2849, 2917, 3022, 3055, 3084, 3314;

^{1}H NMR (CD_3CN, 400 MHz) *δ* 8.78 – 8.69 (m, 1H), 8.35 (d, *J* = 7.8 Hz, 2H), 8.28 (dd, *J* = 8.1, 2.2 Hz, 1H), 8.22 (d, *J* = 8.1 Hz, 2H), 8.04 (d, *J* = 8.2 Hz, 1H), 7.99 (t, *J* = 7.7 Hz, 2H), 7.78 (t, *J* = 7.8 Hz, 2H), 7.29 (dd, *J* = 12.9, 5.6 Hz, 8H), 7.20 – 7.12 (m, 2H), 4.05 (t, *J* = 7.8 Hz, 1H), 3.31 (q, *J* = 6.6 Hz, 2H), 2.36 (q, *J* = 7.3 Hz, 2H);

^{13}C NMR (CD_3CN, 126 MHz) *δ* 163.01, 150.99, 150.78, 144.86 (x3), 140.45 (x2), 139.36, 134.72, 134.71 (x2), 131.54 (x2), 129.46, 128.64 (x2), 128.55 (x4), 127.62 (x4), 126.67, 126.27 (x2), 124.52 (x2), 48.62, 38.65, 34.10.

^{19}F NMR (CD_3CN, 282 MHz) *δ* -79.32;

HR-MS 499.1835 ($C_{33}H_{27}N_2OS^+$) calcd 499.1839.

3) Synthesis of UCB-J labelling precursors 8

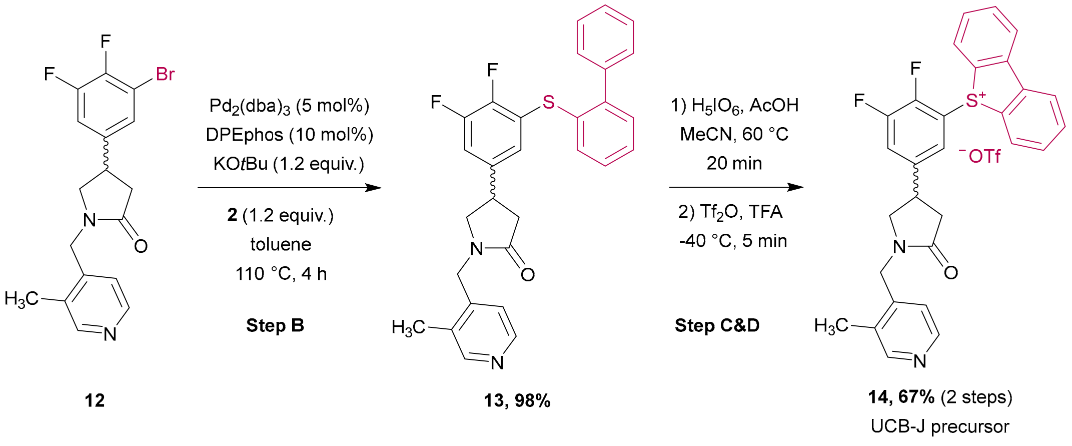

Compound **12** was obtained by custom synthesis from Pharmasynth.

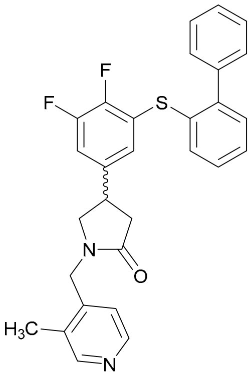

Chemical Formula: $C_{29}H_{24}F_2N_2OS$
Exact Mass: 486.1577
Molecular Weight: 486.5808

4-(3-([1,1'-biphenyl]-2-ylthio)-4,5-difluorophenyl)-1-((3-methylpyridin-4-yl)methyl)pyrrolidine-2-one (13): Prepared following the general procedure for step B (4 hours reaction time) in 98% yield (62 mg) from 49 mg of **12**.

Orange oil; **TLC** *R*f 0.17 (dichloromethane/MeOH 2 %);

IR (neat) ν_{max} 428, 503, 545, 584, 615, 638, 678, 700, 731, 750, 808, 839, 864, 914, 939, 1006, 1072, 1103, 1120, 1132, 1159, 1184, 1220, 1259, 1355, 1435, 1462, 1485, 1566, 1595, 1687, 2924, 3057;

^{1}H NMR (CDCl$_3$, 500 MHz) *δ* 8.44 (s, 2H), 7.45 – 7.26 (m, 9H), 7.07 (d, *J* = 4.9 Hz, 1H), 6.82 (ddd, *J* = 10.5, 6.7, 2.3 Hz, 1H), 6.59 (dt, *J* = 5.6, 1.9 Hz, 1H), 4.61 (d, *J* = 15.6 Hz, 1H), 4.35 (d, *J* = 15.7 Hz, 1H), 3.53 (dd, *J* = 9.6, 8.2 Hz, 1H), 3.42 (p, *J* = 8.3 Hz, 1H), 3.11 (dd, *J* = 9.6, 7.1 Hz, 1H), 2.81 (dd, *J* = 17.0, 8.9 Hz, 1H), 2.44 (dd, *J* = 17.0, 8.5 Hz, 1H), 2.31 (s, 3H);

^{13}C NMR (CDCl$_3$, 126 MHz) *δ* 173.27, 151.65 (d, *J* = 13.5 Hz), 150.19, 149.65 (d, *J* = 13.4 Hz), 149.39 (d, *J* = 13.2 Hz), 147.42 (d, *J* = 13.1 Hz), 146.98, 144.28, 144.13, 140.33, 138.16 (dd, *J* = 4.8, 1.2 Hz), 132.44, 131.65 (d, *J* = 1.0 Hz), 131.14, 129.34 (x2), 128.40, 128.33, 128.10 (x2), 127.69, 126.43 (d, *J* = 14.5 Hz), 125.95 (d, *J* = 2.9 Hz), 114.65 (d, *J* = 18.1 Hz), 53.69, 43.76, 38.23, 36.64, 16.13;

^{19}F NMR (CDCl$_3$, 282 MHz) *δ* -134.38 (d, *J* = 22.1 Hz), -135.23 (d, *J* = 22.1 Hz);

HR-MS 487.1650 ($C_{29}H_{24}F_2N_2OS+H^+$) calcd 487.1650.

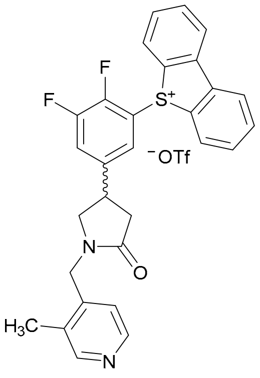

Chemical Formula: $C_{30}H_{23}F_5N_2O_4S_2$
Exact Mass: 485.1494 ($C_{29}H_{23}F_2N_2OS^+$)
Molecular Weight: 634.6360

5-(2,3-difluoro-5-(1-((3-methylpyridin-4-yl)methyl)-5-oxopyrrolidin-3-yl)phenyl)-5H-dibenzo[b,d]thiophen-5-ium trifluoromethanesulfonate (14): Prepared following the procedure for the synthesis of **8**. 20 minutes reaction time for the oxidation step. Obtained in 67% yield (35 mg) from 40 mg of **13**. 1.2 equivalents of Tf_2O and 1 equiv. of TFA were used.

White solid; **mp** 101-102 °C; **TLC** R_f 0.18 (DCM/MeOH 8 %);

IR (neat) ν_{max} 403, 420, 449, 487, 514, 542, 555, 572, 613, 634, 705, 758, 839, 867, 906, 941, 1028, 1066, 1149, 1222, 1253, 1357, 1448, 1496, 1597, 1681, 2852, 2924;

^{1}H NMR (CD_3CN, 500 MHz) *δ* 8.39 – 8.32 (m, 4H), 8.12 (t, *J* = 8.7 Hz, 2H), 7.98 (td, *J* = 7.6, 2.9 Hz, 2H), 7.79 – 7.72 (m, 2H), 7.62 (ddd, *J* = 11.0, 7.4, 2.1 Hz, 1H), 7.10 (dt, *J* = 4.2, 1.9 Hz, 1H), 7.04 (d, *J* = 5.0 Hz, 1H), 4.53 (d, *J* = 15.8 Hz, 1H), 4.27 (d, *J* = 15.8 Hz, 1H), 3.59 (p, *J* = 8.2 Hz, 1H), 3.52 (dd, *J* = 9.6, 8.2 Hz, 1H), 3.11 (dd, *J* = 9.6, 7.0 Hz, 1H), 2.83 – 2.65 (m, 1H), 2.39 (dd, *J* = 16.7, 8.3 Hz, 1H), 2.25 (s, 3H);

^{13}C NMR (CD_3CN, 126 MHz) *δ* 174.04, 153.13 (d, *J* = 11.4 Hz), 152.26, 151.48, 151.39 (d, *J* = 5.8 Hz), 149.71 (d, *J* = 15.5 Hz), 149.07, 145.13, 144.52 (t, *J* = 5.1 Hz), 141.55 (x2), 136.54 (x2), 133.41 (x2), 130.78 (d, *J* = 5.6 Hz), 129.92 (d, *J* = 2.3 Hz), 127.31, 126.33 (x2), 125.46 (d, *J* = 17.7 Hz), 123.78, 121.65, 116.62 (d, *J* = 11.1 Hz), 54.42, 44.62, 39.13, 37.97, 16.63;

^{19}F NMR (CD_3CN, 282 MHz) *δ* -79.38, -132.96 (d, *J* = 20.3 Hz), -136.28 (d, *J* = 20.3 Hz);

HR-MS 485.1490 ($C_{29}H_{23}F_2N_2OS^+$) calcd 485.1494.

[^{18}F] Radiolabeling of Dibenzothiophenium Salts

1) General considerations and description

All labeling reactions were performed manually using [^{18}F]fluoride (150-400 MBq) in [^{18}O]H_2O. Radio-HPLC were performed with an Agilent 1200 HPLC system equipped with a 1200 Series Diode Array Detector and a GABI Star NaI(Tl) scintillation detector (energy window 400-700 keV). The system was used for purification as well as characterization of radiotracers. Columns and conditions used for purification and quality controls (QC) are indicated for each tracer in corresponding section.

- **Radiochemical Conversion (RCC):**

Radiochemical conversion (RCC) were determined using the semi-preparative radio-HPLC chromatograms of the quenched crude labelling mixture and refer to the area under the curve (AUC) of the radioactive peak of interest divided by the summed AUC of all other radioactive peaks ([^{18}F]fluoride and potential side-products).

- **Activity yield (AY)**

Activity yield (AY) refer to the activity of the pure tracer isolated after HPLC divided by the initial activity of [^{18}F]fluoride in [^{18}O]H_2O used for the labeling (not corrected for decay, excluding losses during QMA trapping and release, typical QMA elution efficiency is above 90%).

- **Radiochemical Yield (RCY):**

Radiochemical yield (RCY) refer to the activity of the pure tracer isolated after HPLC divided by the initial activity of [^{18}F]fluoride in [^{18}O]H_2O used for the labeling decay corrected to the same point in time (excluding losses during QMA trapping and release, typical QMA elution efficiency is above 90%).

2) General procedure E: [^{18}F]radiolabeling of dibenzothiophenium salts

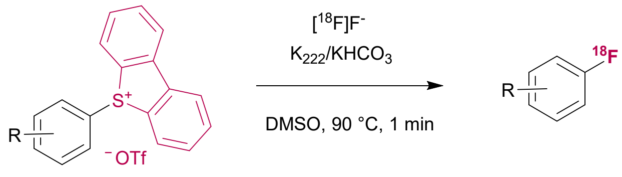

- **QMA cartridge conditioning:**

Sep-Pak Accell Plus QMA Plus Light Cartridge (130 mg, Waters Cat. No. WAT023525) was flushed successively with aqueous sodium hydroxide (1M, 5 mL), HPLC water (10 mL), aqueous potassium carbonate (1M, 1 mL), HPLC water (10 mL) and air-dried (10 mL).

- **[^{18}F] Radiolabeling procedure**

[^{18}F]fluoride in ^{18}O-water was trapped on a Sep-Pak® QMA cartridge and released with a solution Kryptofix 222 (30 mM) and potassium bicarbonate (30 mM) in acetonitrile/water (85%/15% v/v; 0.5 mL). The solvent was removed by heating at 90 °C under a stream of nitrogen. [^{18}F]fluoride was dried by azeotropic distillation with acetonitrile (2× 0.5 mL; 90 °C) and the reaction vial was subsequently capped. The vial was preheated at 90 °C for 1 min. The corresponding sulfonium salt (2 mg) in anhydrous DMSO (0.5 mL) was added to the reaction vial and stirred at 90 °C for 1 min. Then, cold water (1 mL) was immediately added to the reaction and purified using semi-preparative HPLC.

3) Radiolabeling of [^{18}F]AldoView

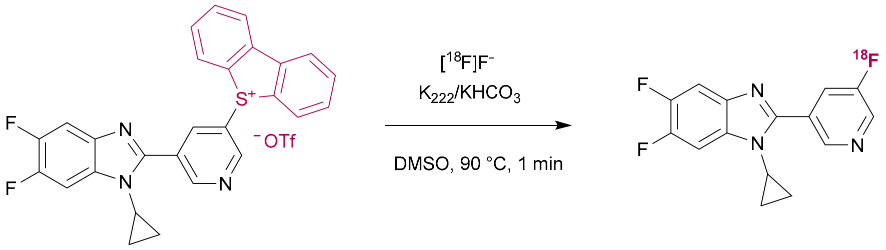

8, 2 mg
AldoView precursor

[^{18}F]AldoView
76 ± 5% RCC (*n* = 3)
51 ± 4% RCY (*n* = 3)
39 ± 3% AY (*n* = 3)
4.1 ± 0.4 GBq/µmol starting from ≈300MBq (*n* = 2)

[^{18}F]AldoView was labelled according to general procedure E using 2 mg of dibenzothiophenium precursor **8**. [^{18}F]AldoView was obtained with 76 ± 5% RCC (*n* = 3), 39 ± 3% AY (*n* = 3), 51% ± 4% RCY (*n* = 3), with a radiochemical purity >99% and with a molar activity of 4.1 ± 0.4 GBq/µmol (*n* = 2) when starting from 300MBq of [^{18}F]fluoride.

- **Semi-preparative HPLC**

Column: Phenomenex Luna C-18(2) column (5 µm; 250 × 10 mm) at room temperature

Retention time: radio-HPLC = 20.13 min, UV (254nm) = 20.04 min.

Methods: Flow rate : 4.00 mL/min

Time (min)	H_2O + 0.5% TFA (%)	MeOH + 0.5% TFA (%)
0.00	55	45
30.00	55	45
30.01	5	95
40.00	5	95

Chromatogram:

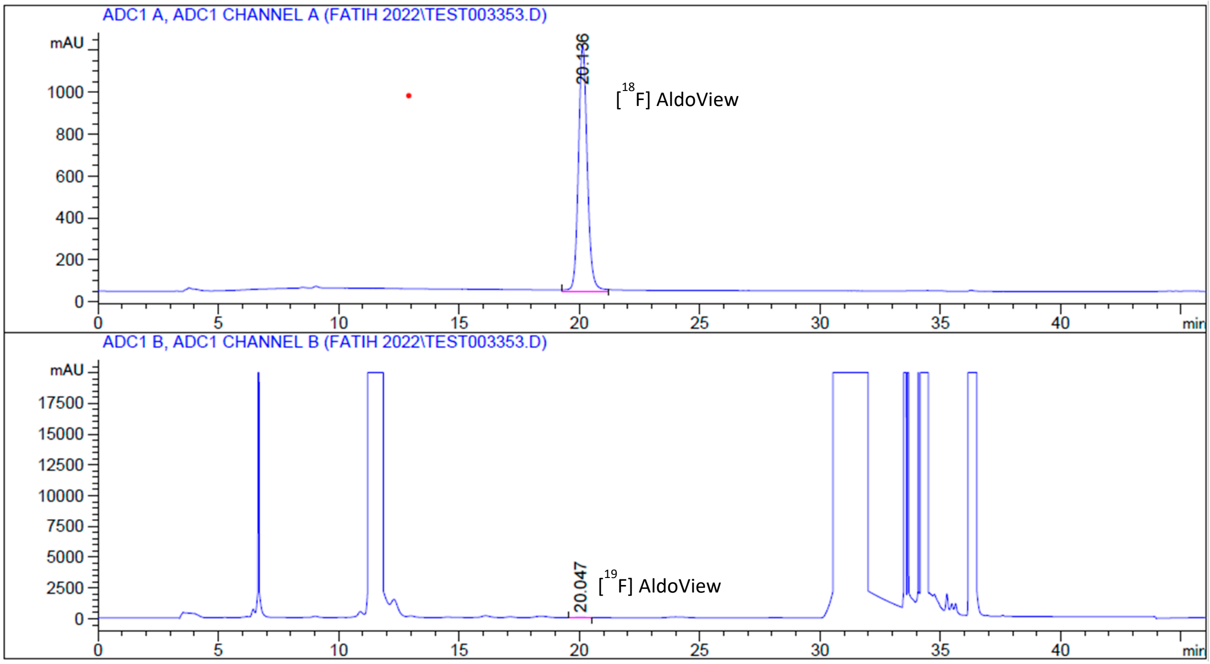

Figure 2 : semi-preparative HPLC chromatogram of [^{18}F]AldoView labeling. Radio-HPLC (top), UV = 254nm (bottom).

- **Quality control HPLC**

Column: Agilent Eclipse Plus C-18 column (5 µm; 150 × 4.6 mm) at room temperature

Retention time: radio-HPLC = 8.99 min, UV (254nm) = 8.69 min.

Methods: Flow rate : 1.80 mL/min

Time (min)	H_2O + 0.1% TFA (%)	MeOH + 0.1% TFA (%)
0.00	70	30
2.00	70	30
12.00	30	70
12.01	5	95
14.00	5	95
15.00	70	30

Chromatograms:

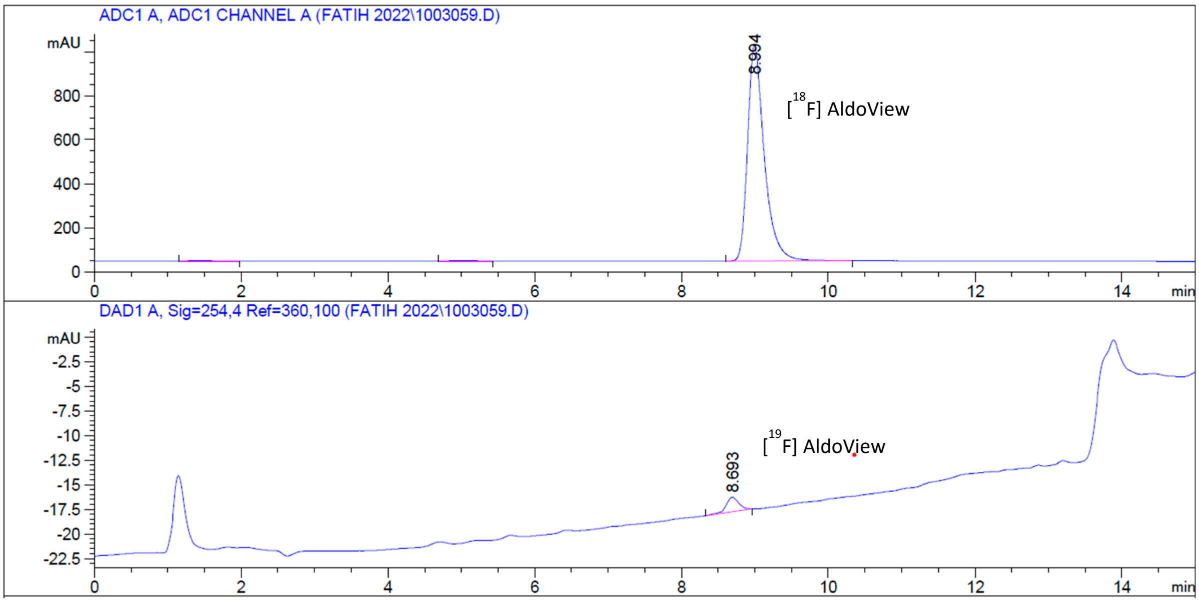

Figure 3 : Analytical HPLC chromatogram of the isolated product [^{18}F]AldoView. Radio-HPLC (top), UV = 254nm (bottom).

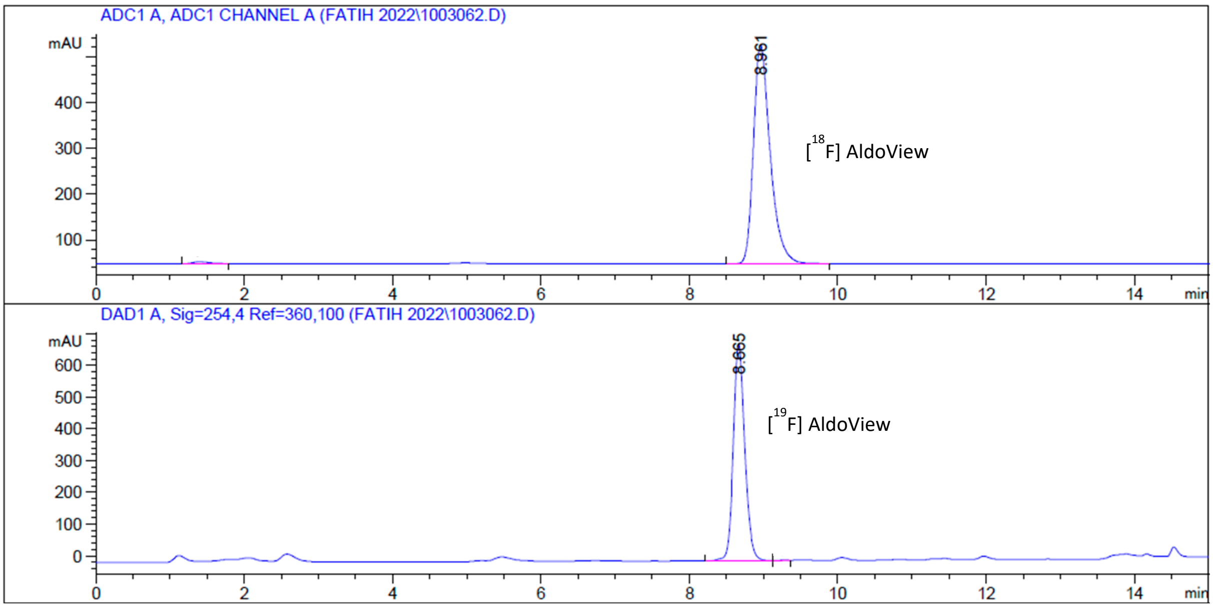

Figure 4 : Analytical HPLC chromatogram of the isolated product [^{18}F]AldoView co-injected with the non-radioactive AldoView reference compound. Radio-HPLC (top), UV = 254nm (bottom).

- **Calibration curve**

The same HPLC column and method as the Quality control HPLC was used to prepare the calibration curve. AldoView reference compound was used and samples were diluted in MeOH/H_2O (30%/70% v/v). Calibration curve was plotted as mAU=f(µmol) and used to determine the molar activity.

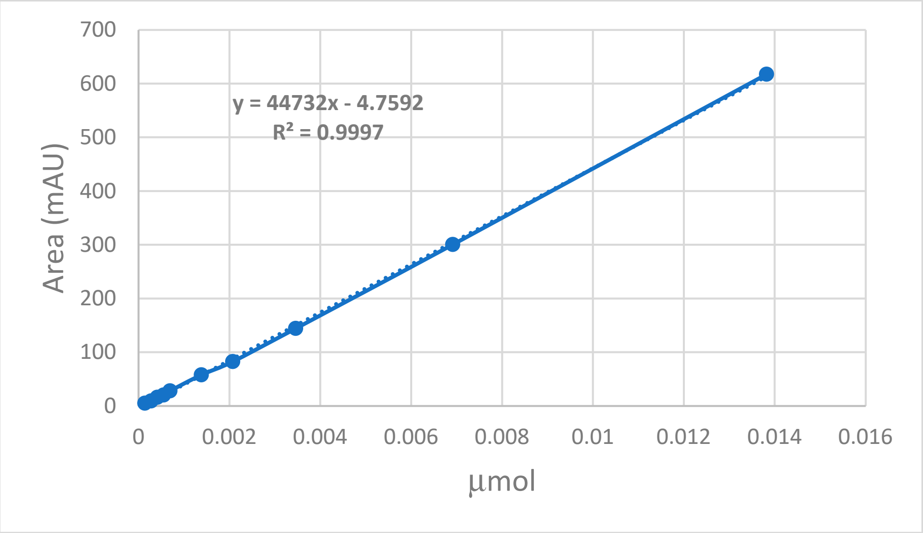

Figure 5 : Calibration curve of AldoView reference compound: mAU=f(µmol)

4) Radiolabeling of [^{18}F]FNDP

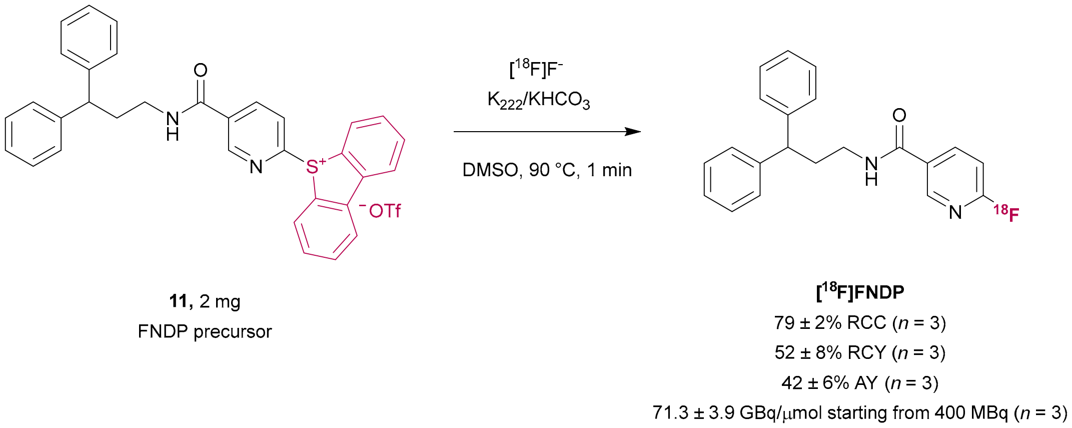

[^{18}F]FNDP was labelled according to general procedure E using 2 mg of dibenzothiophenium precursor **11**. [^{18}F]FNDP was obtained with 79 ± 2% RCC (*n* = 3), 42 ± 6% AY (*n* = 3), 52% ± 8% RCY (*n* = 3), with a radiochemical purity >99% and with a molar activity of 71.3 ± 3.9 GBq/µmol (*n* = 3) when starting from 421 MBq of [^{18}F]fluoride.

- **Semi-preparative HPLC**

Column: Phenomenex Luna C-18(2) column (5 µm; 250 × 10 mm) at room temperature

Retention time: radio-HPLC = 10.24 min, UV (254nm) = 9.15 min.

Methods: Flow rate: 4.00 mL/min

**Time (min)**	**H_2O + 0.5% TFA (%)**	**MeOH + 0.5% TFA (%)**
0.00	30	70
20.00	30	70
20.01	5	95
28.00	5	95

Chromatogram:

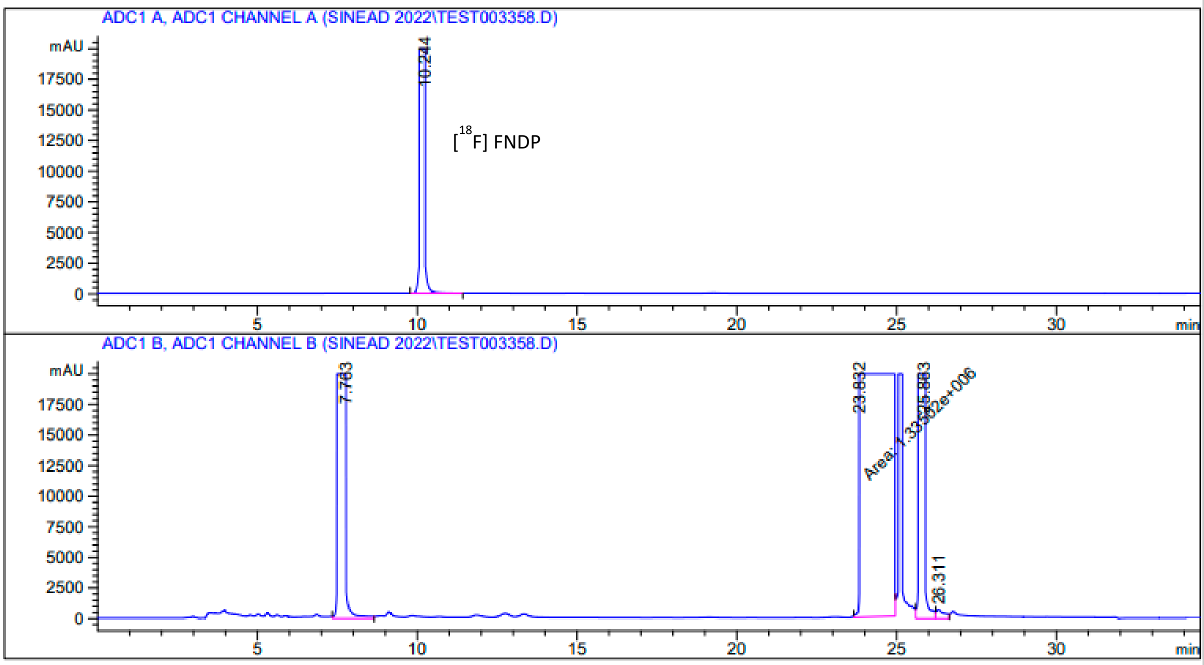

Figure 6: semi-preparative HPLC chromatogram of [^{18}F]FNDP labeling. Radio-HPLC (top), UV = 254nm (bottom).

- **Quality control HPLC**

Column: Chromolith Performance C-18 column (5 µm; 150 × 4.6 mm) at room temperature

Retention time: radio-HPLC = 5.55 min, UV (254nm) = 5.38 min.

Methods: Flow rate : 3 mL/min

**Time (min)**	**H_2O + 0.1% TFA (%)**	**MeOH + 0.1% TFA (%)**
0.00	80	30
8.00	30	70
10.00	80	20

Chromatograms:

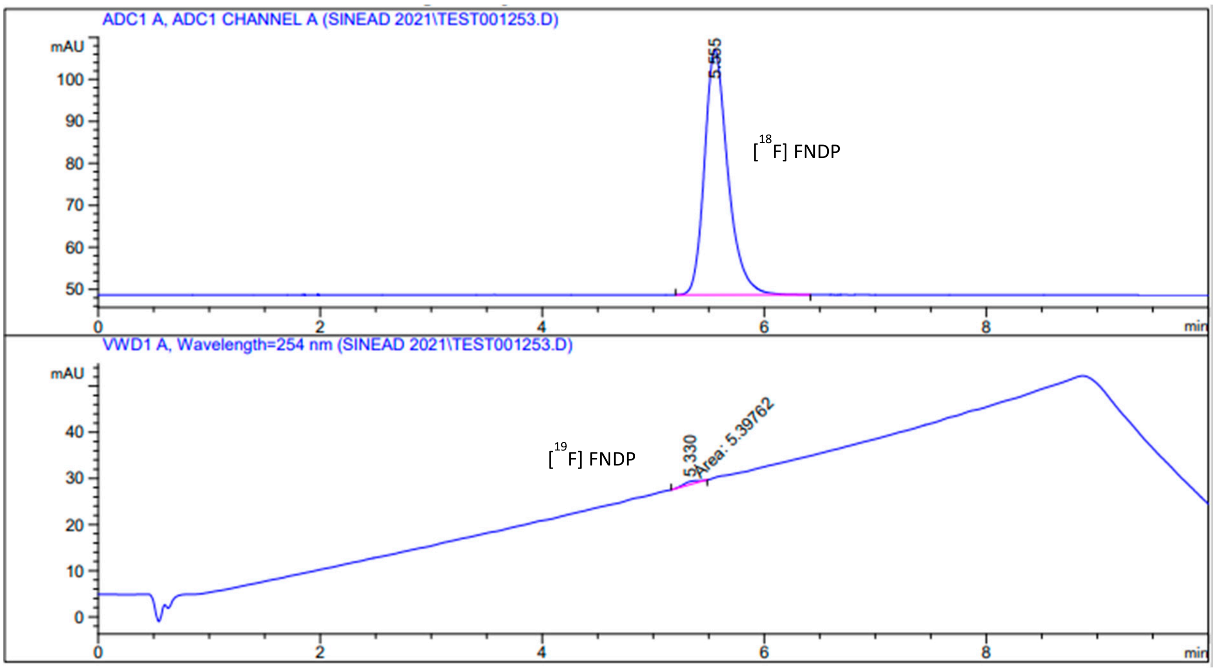

Figure 7: Analytical HPLC chromatogram of the isolated product [^{18}F]FNDP, radio-HPLC (top), UV = 254nm (bottom).

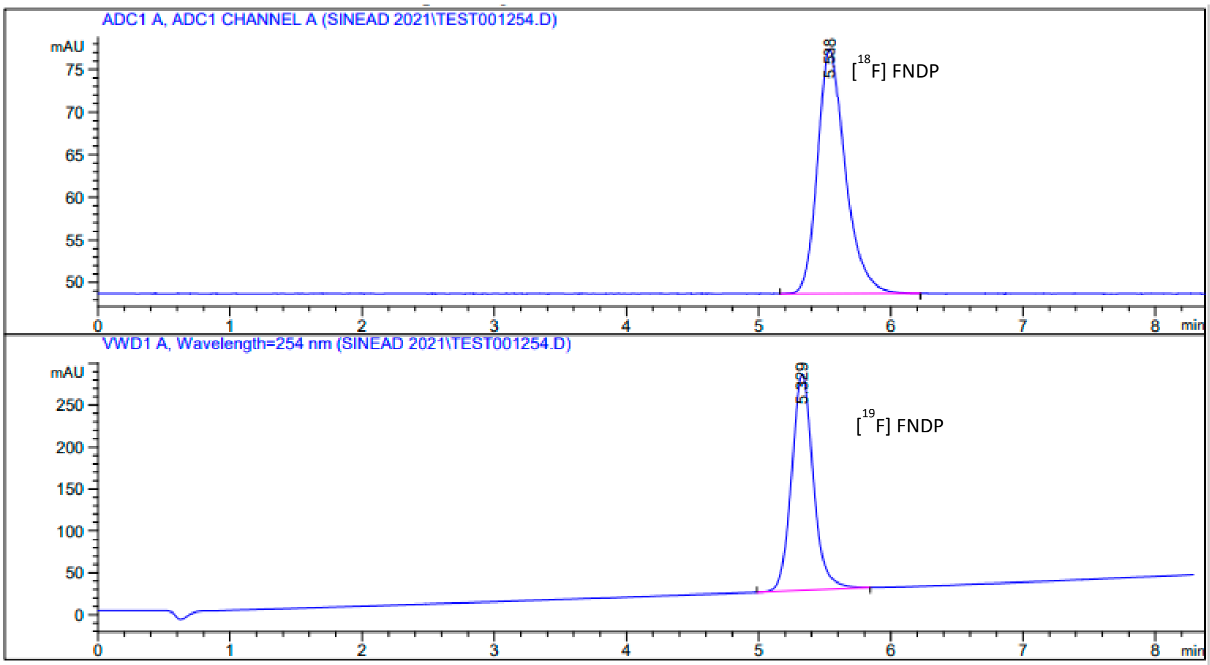

Figure 8: Analytical HPLC chromatogram of the isolated product [^{18}F]FNDP co-injected with the non-radioactive FNDP reference compound. Radio-HPLC (top), UV = 254nm (bottom).

- **Calibration curve**

The same HPLC column and method as the Quality control HPLC was used to prepare the calibration curve. FNDP reference compound was used and samples were diluted in MeOH/H_2O (30%/70% v/v). Calibration curve was plotted as mAU=f(µmol) and used to determine the molar activity.

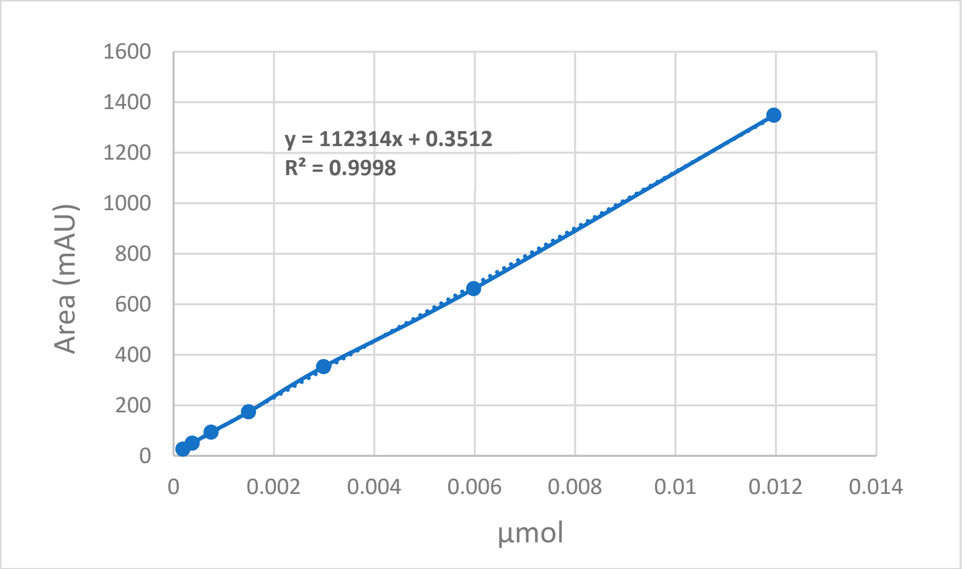

Figure 9: Calibration curve of FNDP reference compound: mAU=f(mmol)

5) Radiolabeling of [^{18}F]UCB-J

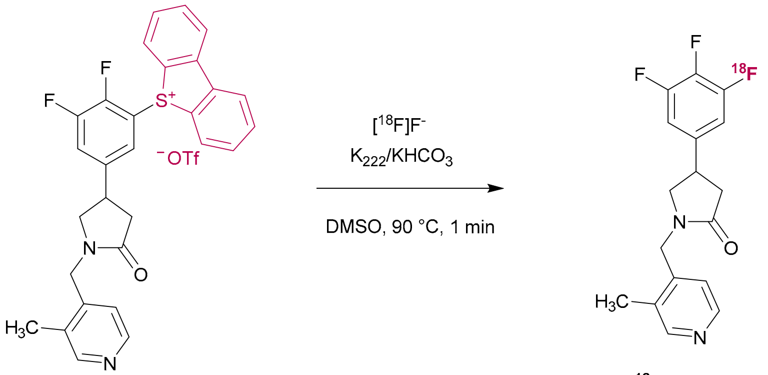

14, 2 mg
UCB-J precursor

[^{18}F]UCB-J
50 ± 5% RCC (*n* = 4)
24 ± 2% RCY (*n* = 4)
19 ± 1% AY (*n* = 4)
66 ± 6.7 µg/mL total no-radioactive reference (*n* = 3)

[^{18}F]UCB-J was labelled according to general procedure E using 2 mg of dibenzothiophenium precursor. [^{18}F]UCB-J was obtained with 50 ± 5% RCC (*n* = 4), 19 ± 1% AY (*n* = 4), 24 ± 2% RCY (*n* = 4), with a radiochemical purity >99% and a total amount of non-radioactive reference of 66 ± 6.7 µg (*n* = 3).

- **Semi-preparative HPLC**

Column: Phenomenex Luna C-18(2) column (5 µm; 250 × 10 mm) at room temperature

Retention time: radio-HPLC = 14.36 min, UV (254nm) = 14.28 min.

Methods: Flow rate : 3.00 mL/min

**Time (min)**	**H_2O + 0.05% HCl (%)**	**EtOH + 0.05% HCl (%)**
0.00	75	25
3.00	75	25
3.01	70	30
28.00	70	30
28.01	5	95
33.00	5	95

Chromatogram:

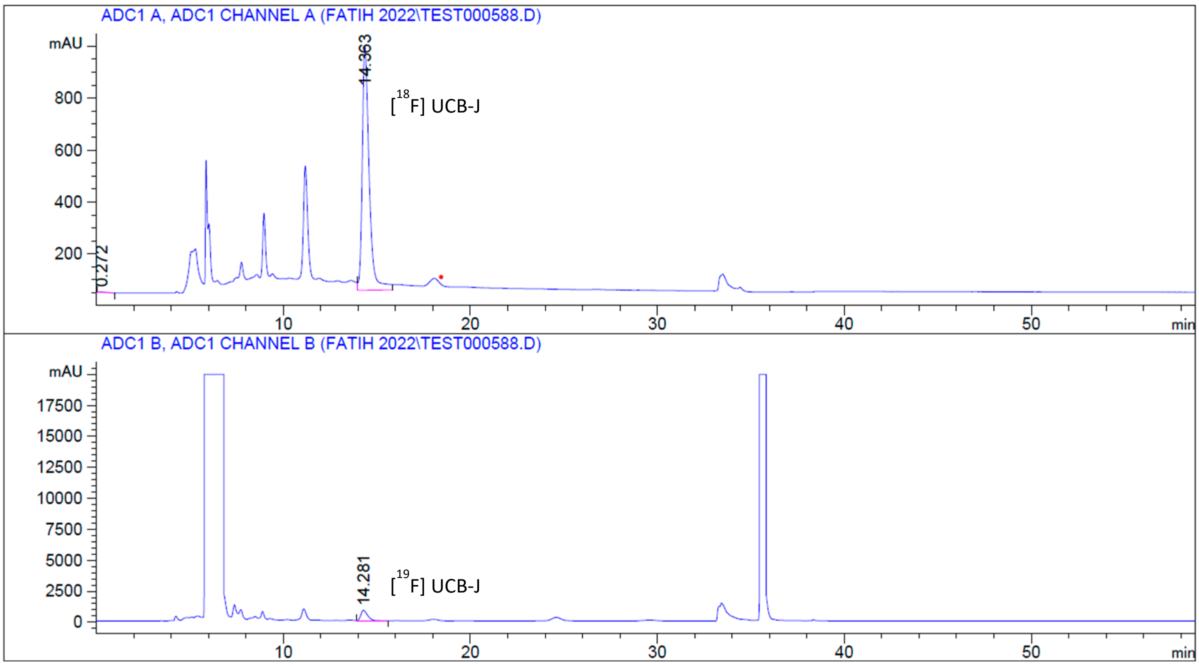

Figure 10 : semi-preparative HPLC chromatogram of [^{18}F]UCB-J labeling. Radio-HPLC (top), UV = 254nm (bottom).

- **Quality control HPLC**

Column: Agilent Eclipse Plus C-18 column (5 µm; 150 × 4.6 mm) at room temperature

Retention time: radio-HPLC = 9.60 min, UV (254nm) = 9.29 min.

Methods: Flow rate : 1.80 mL/min

**Time (min)**	**H_2O + 0.5% AcOH / 0.1M ammonium formate (%)**	**MeCN + 0.5% AcOH (%)**
0.00	85	15
3.00	85	15
12.00	45	55
12.01	5	95
14.00	5	95
14.01	85	15
16.00	85	15

Chromatograms:

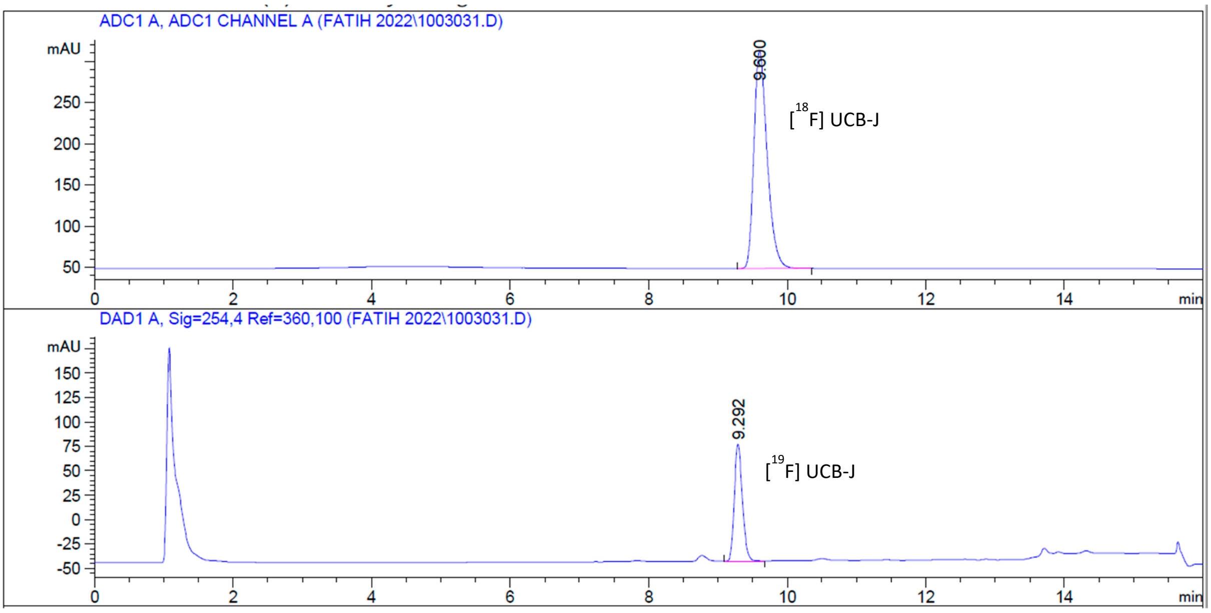

Figure 11 : Analytical HPLC chromatogram of the isolated product [^{18}F]UCB-J. Radio-HPLC (top), UV = 254nm (bottom).

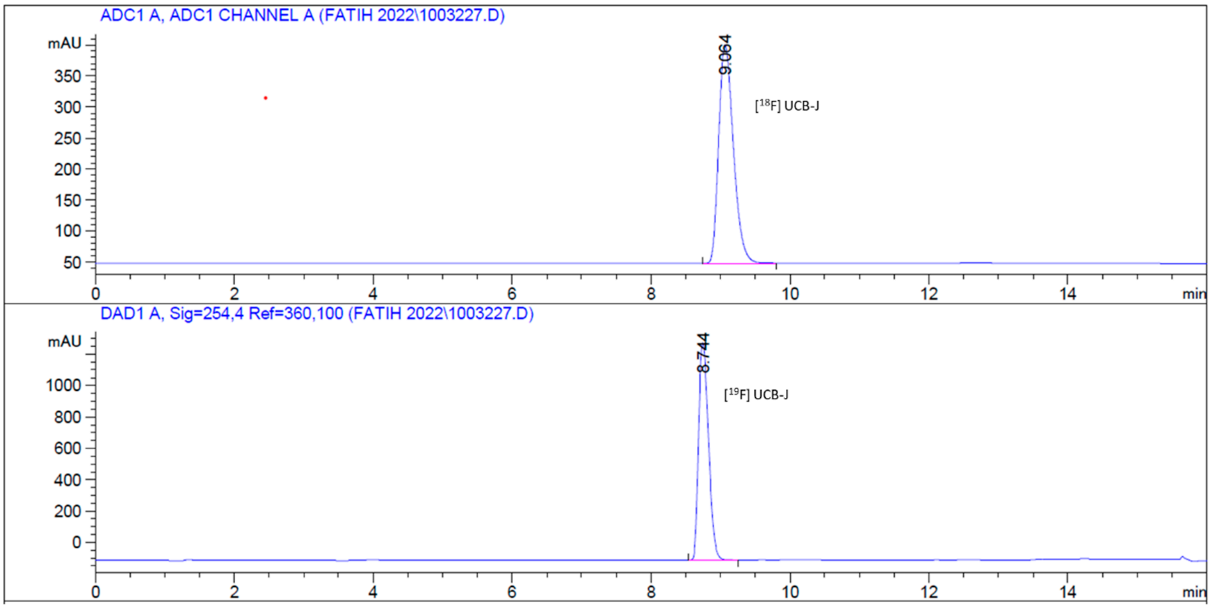

Figure 12 : Analytical HPLC chromatogram of the isolated product [^{18}F]UCB-J co-injected with the non-radioactive UCB-J reference compound. Radio-HPLC (top), UV = 254nm (bottom).

- **Calibration curve**

The same HPLC column and method as the Quality control HPLC was used to prepare the calibration curves. UCB-J reference compound was used. Samples were diluted in EtOH + 0.05% HCl / H_2O + 0.05 HCl (30%/70% v/v), to respect the same composition of the isolated fraction after semi-preparative HPLC. Two calibration curves were plotted: mAU=f(µg) was used to determine the total amount of cold in the isolated product; mAU=f(µmol) was used to determine the molar activity.

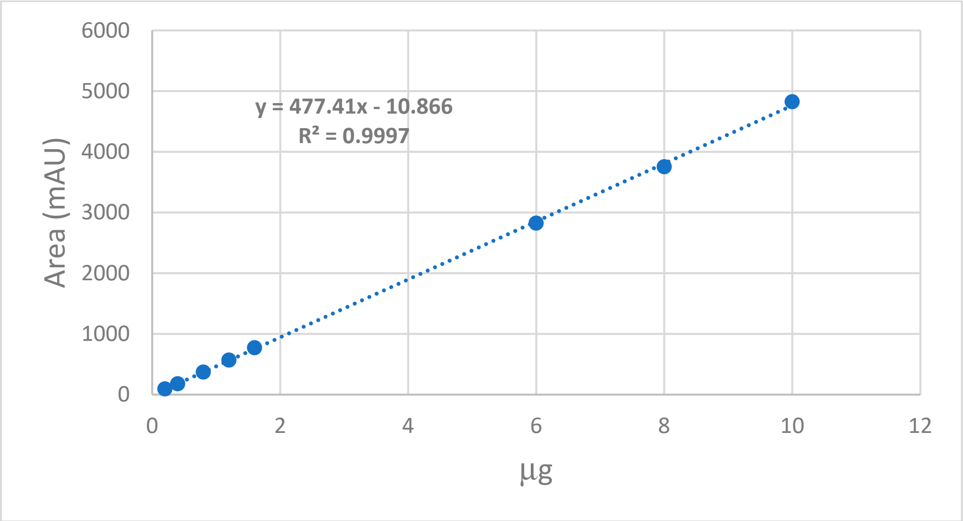

Figure 13 : Calibration curve of UCB-J reference compound: mAU=f(µg)

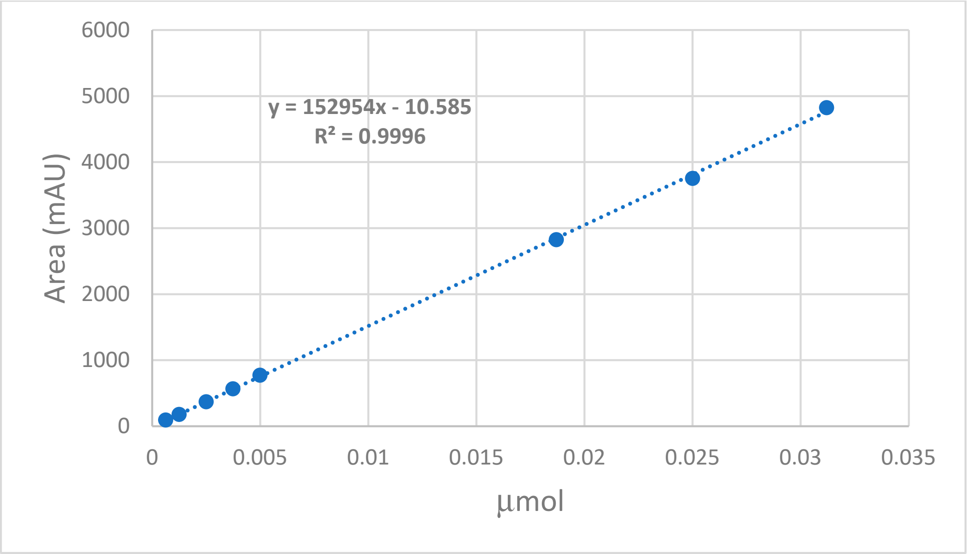

Figure 14 : Calibration curve of UCB-J reference compound: mAU=f(µmol)

NMR Spectra: ^{1}H, ^{13}C and ^{18}F

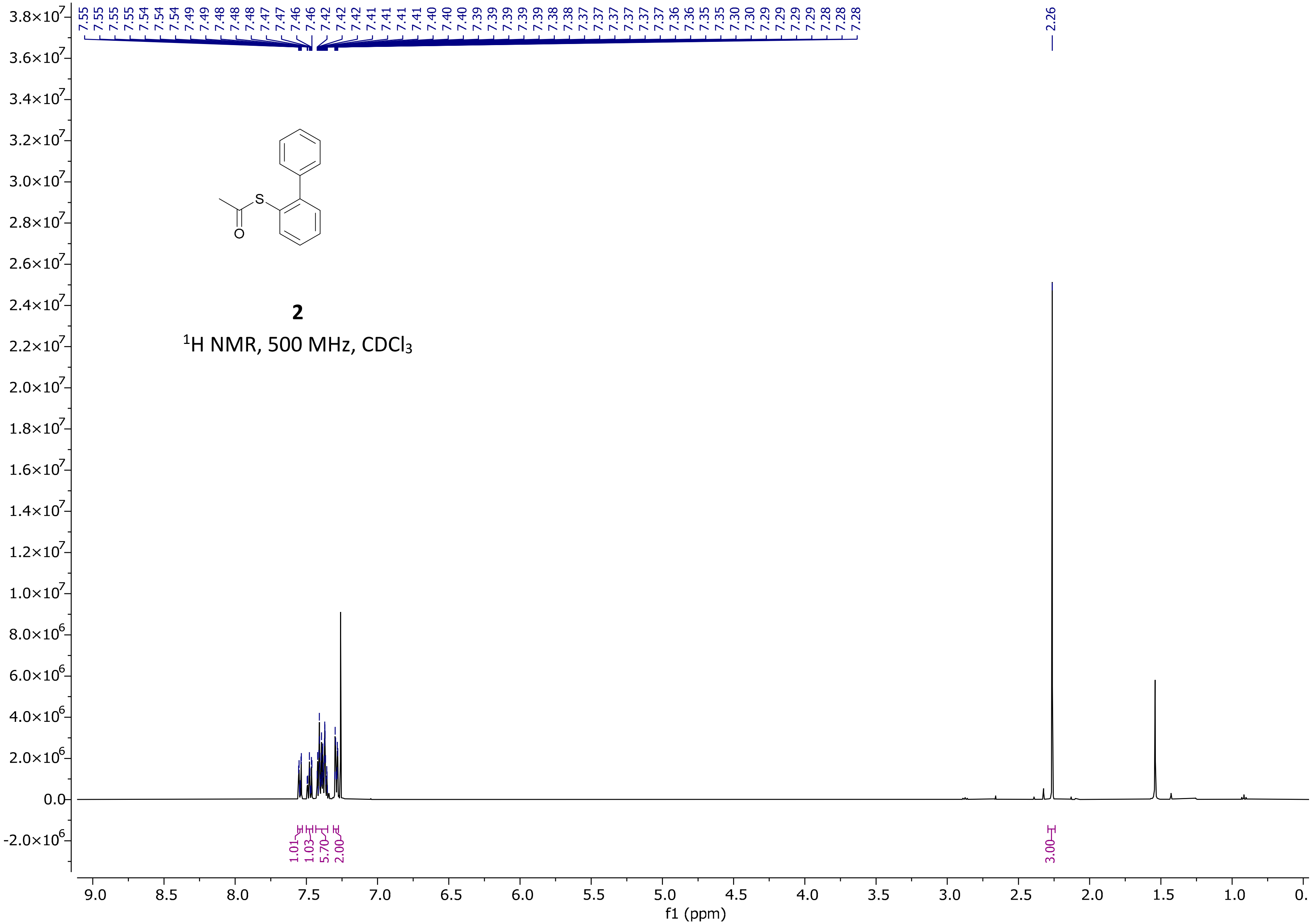
7.55 7.55 7.55 7.55 7.54 7.54 7.49 7.49 7.48 7.48 7.48 7.47 7.47 7.46 7.46 7.42 7.42 7.42 7.41 7.41 7.41 7.40 7.40 7.40 7.39 7.39 7.39 7.39 7.38 7.38 7.37 7.37 7.37 7.37 7.37 7.37 7.36 7.36 7.35 7.35 7.30 7.29 7.29 7.29 7.29 7.28 7.28 7.28
2.26
S
O
2
1H NMR, 500 MHz, CDCl3
1.01
1.03
5.70
2.00
3.00
f1 (ppm)

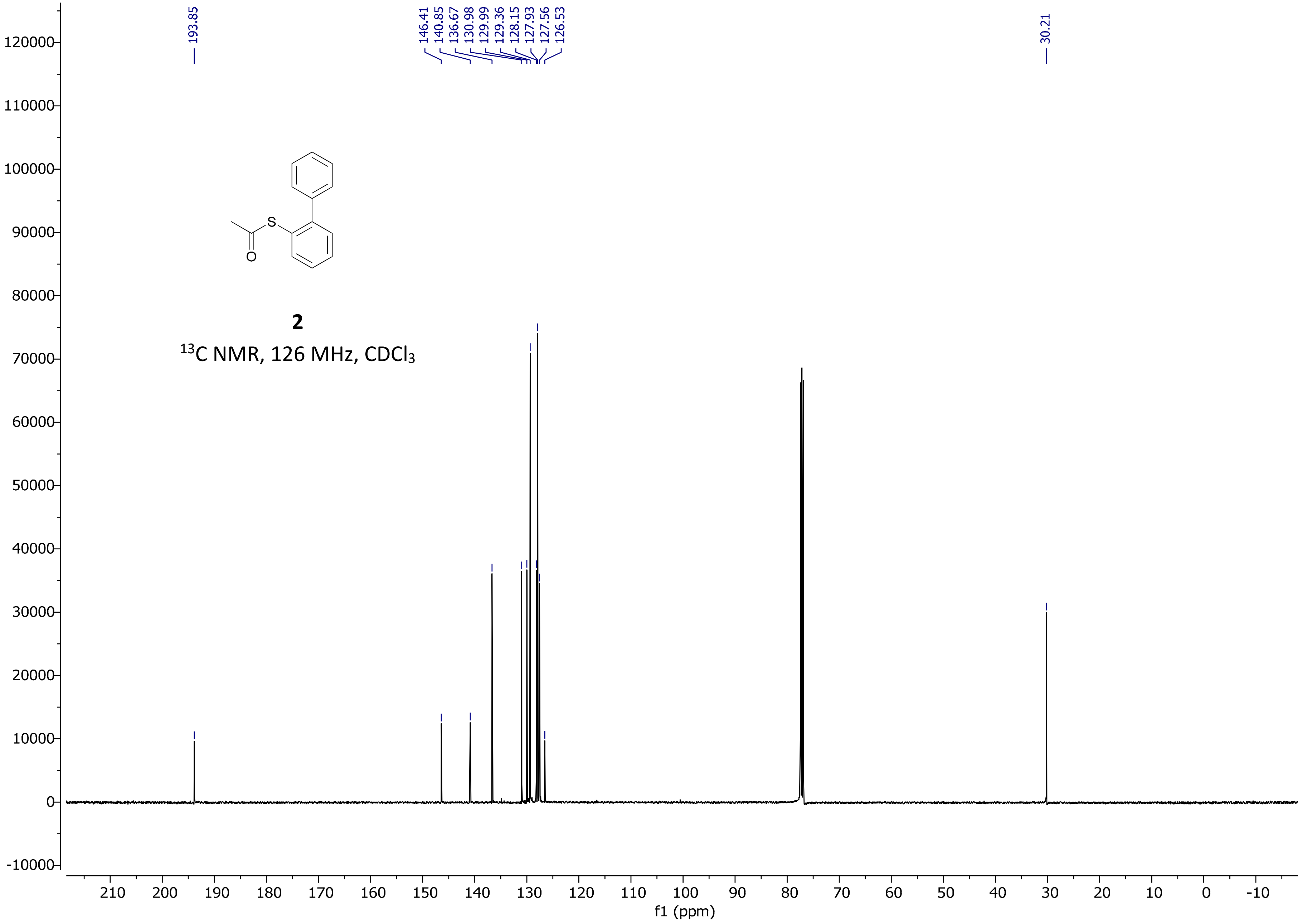
193.85
146.41
140.85
136.67
130.98
129.99
129.36
128.15
127.93
127.56
126.53
30.21
S
O
2
13C NMR, 126 MHz, CDCl3
f1 (ppm)

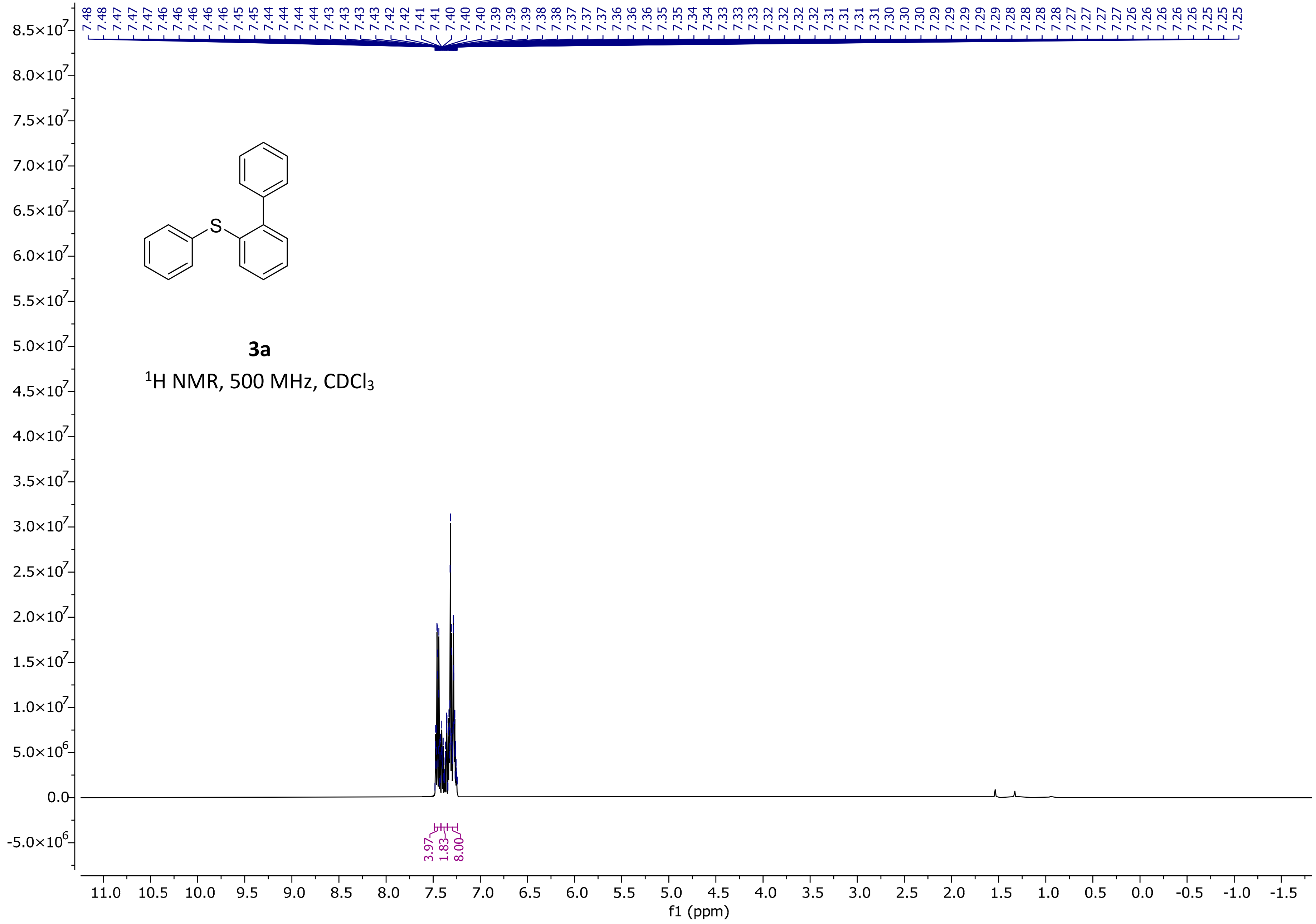

3a

^{1}H NMR, 500 MHz, $CDCl_3$

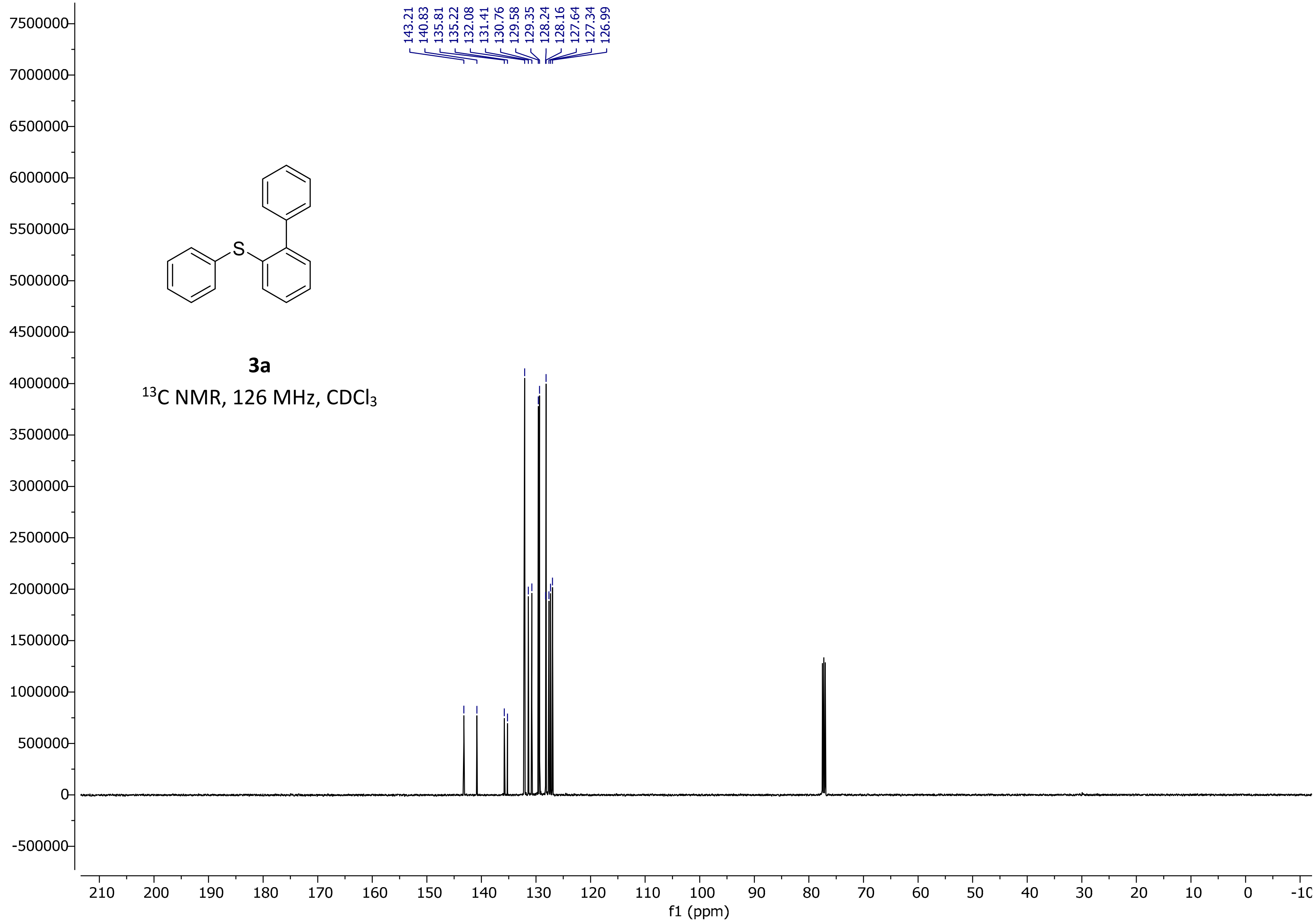
143.21
140.83
135.81
135.22
132.08
131.41
130.76
129.58
129.35
128.24
128.16
127.64
127.34
126.99
S
3a
^{13}C NMR, 126 MHz, $CDCl_3$
f1 (ppm)

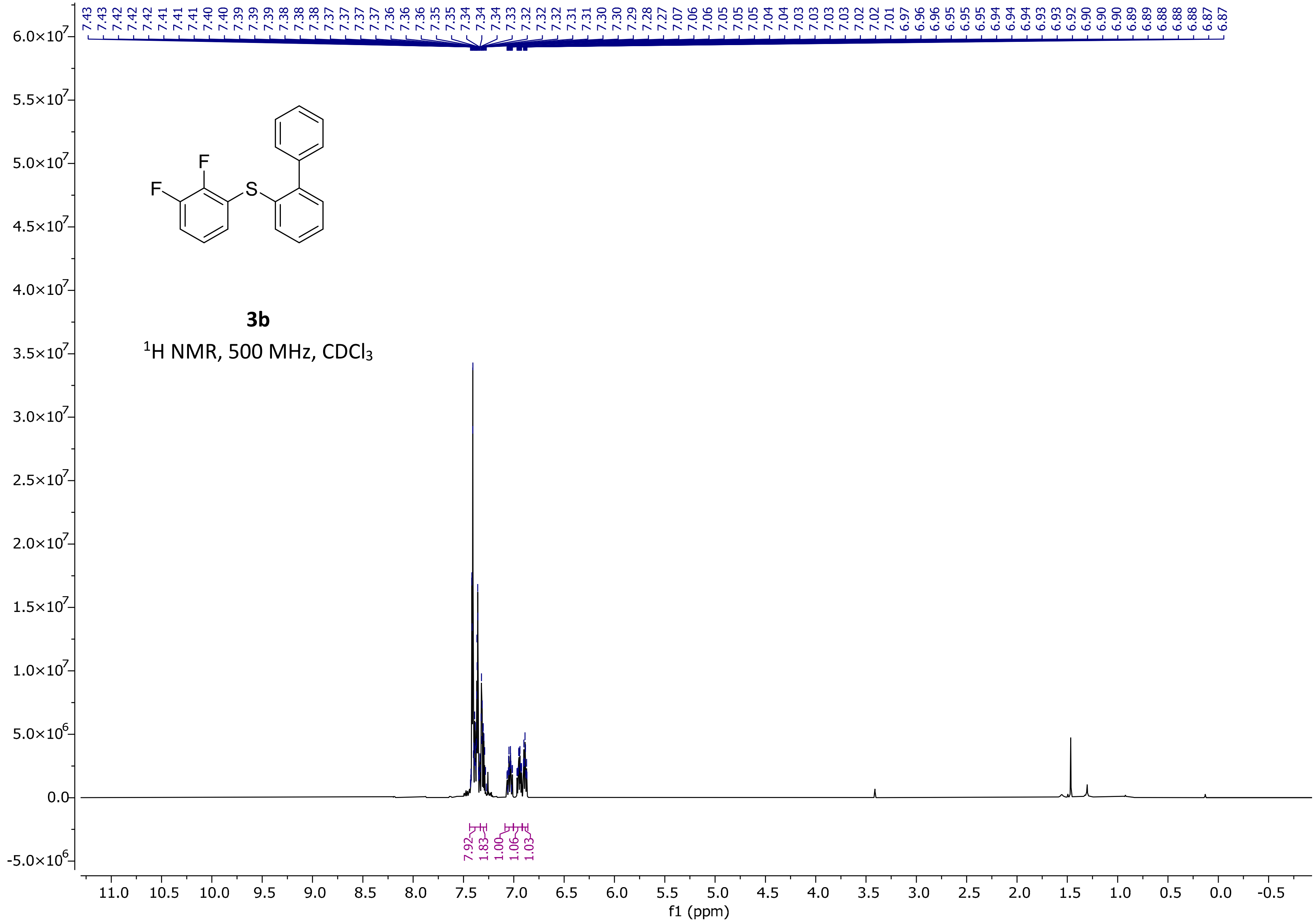

3b

^{1}H NMR, 500 MHz, $CDCl_3$

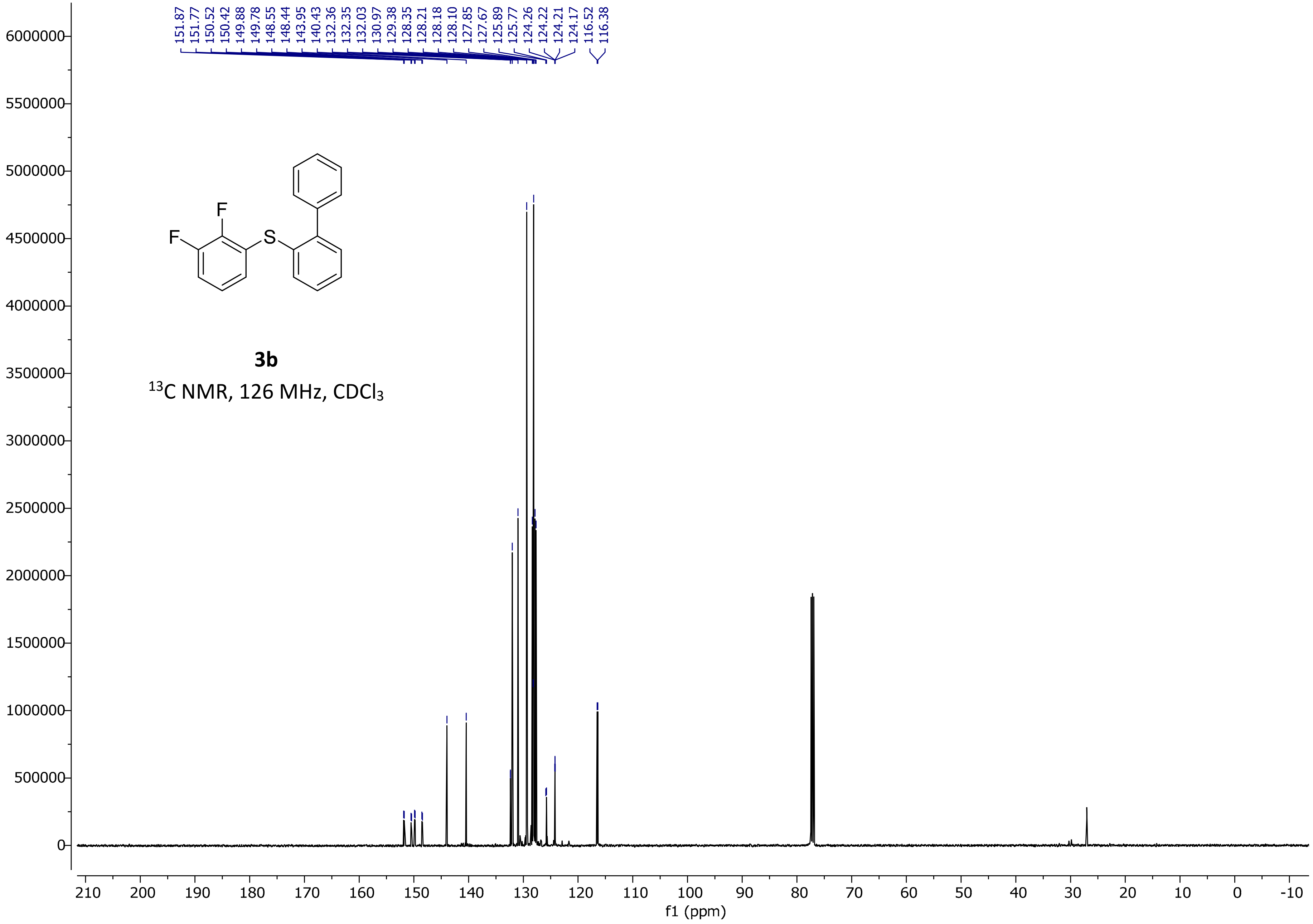
151.87
151.77
150.52
150.42
149.88
149.78
148.55
148.44
143.95
140.43
132.36
132.35
132.03
130.97
129.38
128.35
128.21
128.18
128.10
127.85
127.67
125.89
125.77
124.26
124.22
124.21
124.17
116.52
116.38
F
F
S
3b
^{13}C NMR, 126 MHz, $CDCl_3$
f1 (ppm)

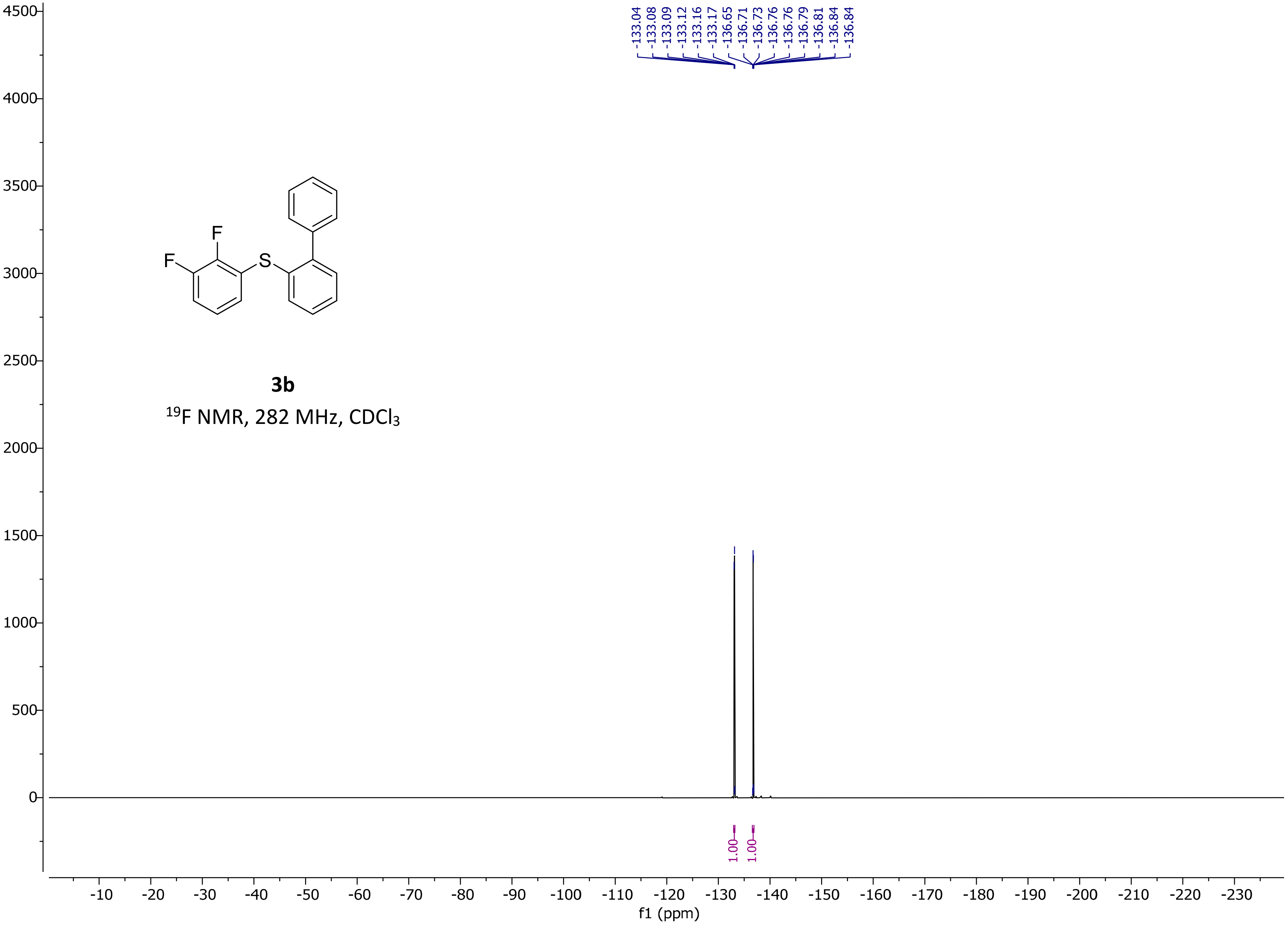
-133.04
-133.08
-133.09
-133.12
-133.16
-133.17
-136.65
-136.71
-136.73
-136.76
-136.76
-136.79
-136.81
-136.84
-136.84
F
F
S
3b
19F NMR, 282 MHz, CDCl3
4500
4000
3500
3000
2500
2000
1500
1000
500
0
1.00
1.00
-10 -20 -30 -40 -50 -60 -70 -80 -90 -100 -110 -120 -130 -140 -150 -160 -170 -180 -190 -200 -210 -220 -230
f1 (ppm)

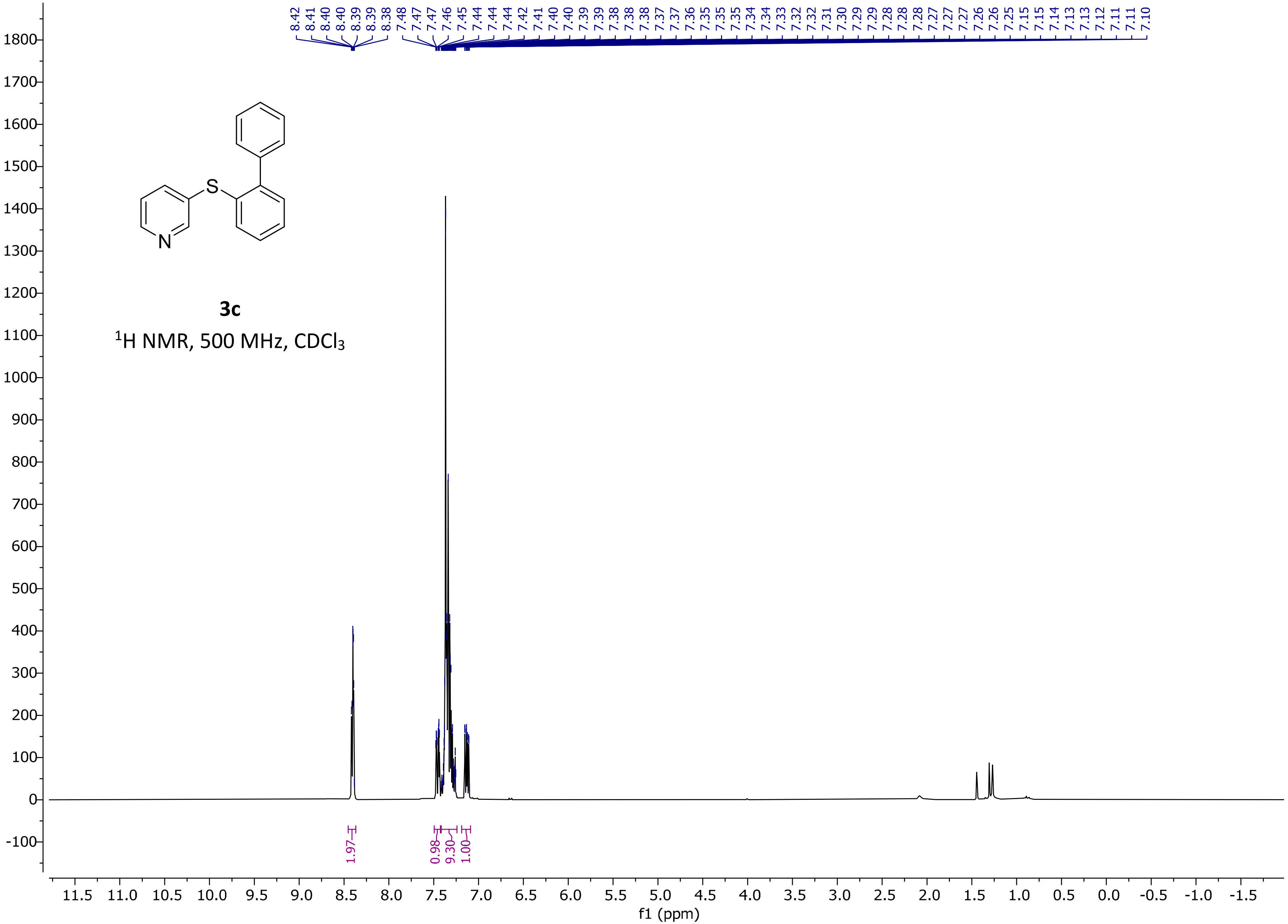
8.42 8.41 8.40 8.40 8.39 8.39 8.38
7.48 7.47 7.47 7.46 7.45 7.44 7.44 7.44 7.42 7.41 7.40 7.40 7.39 7.39 7.38 7.38 7.38 7.37 7.37 7.36 7.35 7.35 7.35 7.34 7.34 7.33 7.32 7.32 7.31 7.30 7.29 7.29 7.28 7.28 7.28 7.27 7.27 7.27 7.26 7.26 7.25 7.15 7.15 7.14 7.13 7.13 7.12 7.11 7.11 7.10
S
N
3c
1H NMR, 500 MHz, CDCl3
1.97
0.98
9.30
1.00
f1 (ppm)

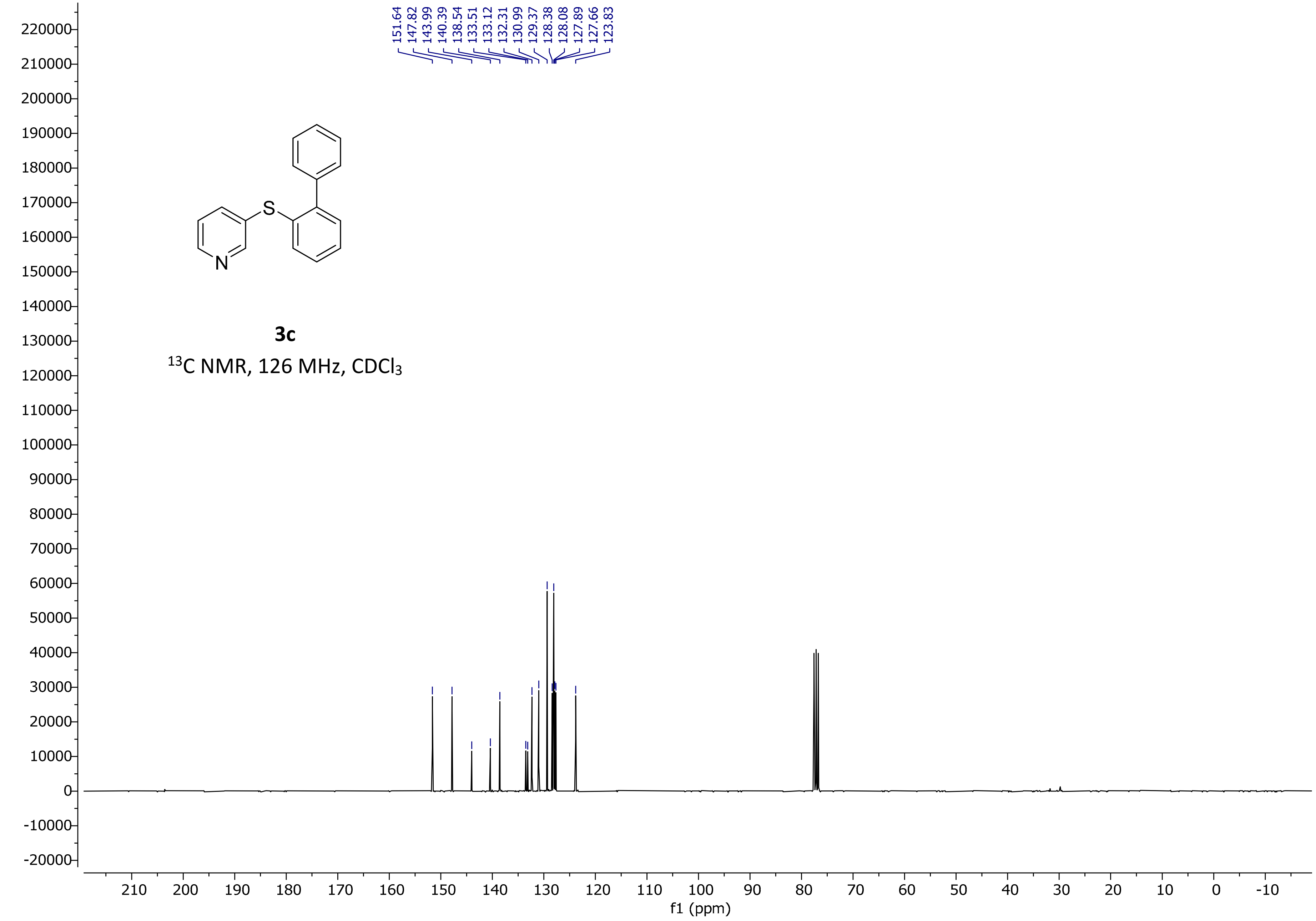

3c

^{13}C NMR, 126 MHz, $CDCl_3$

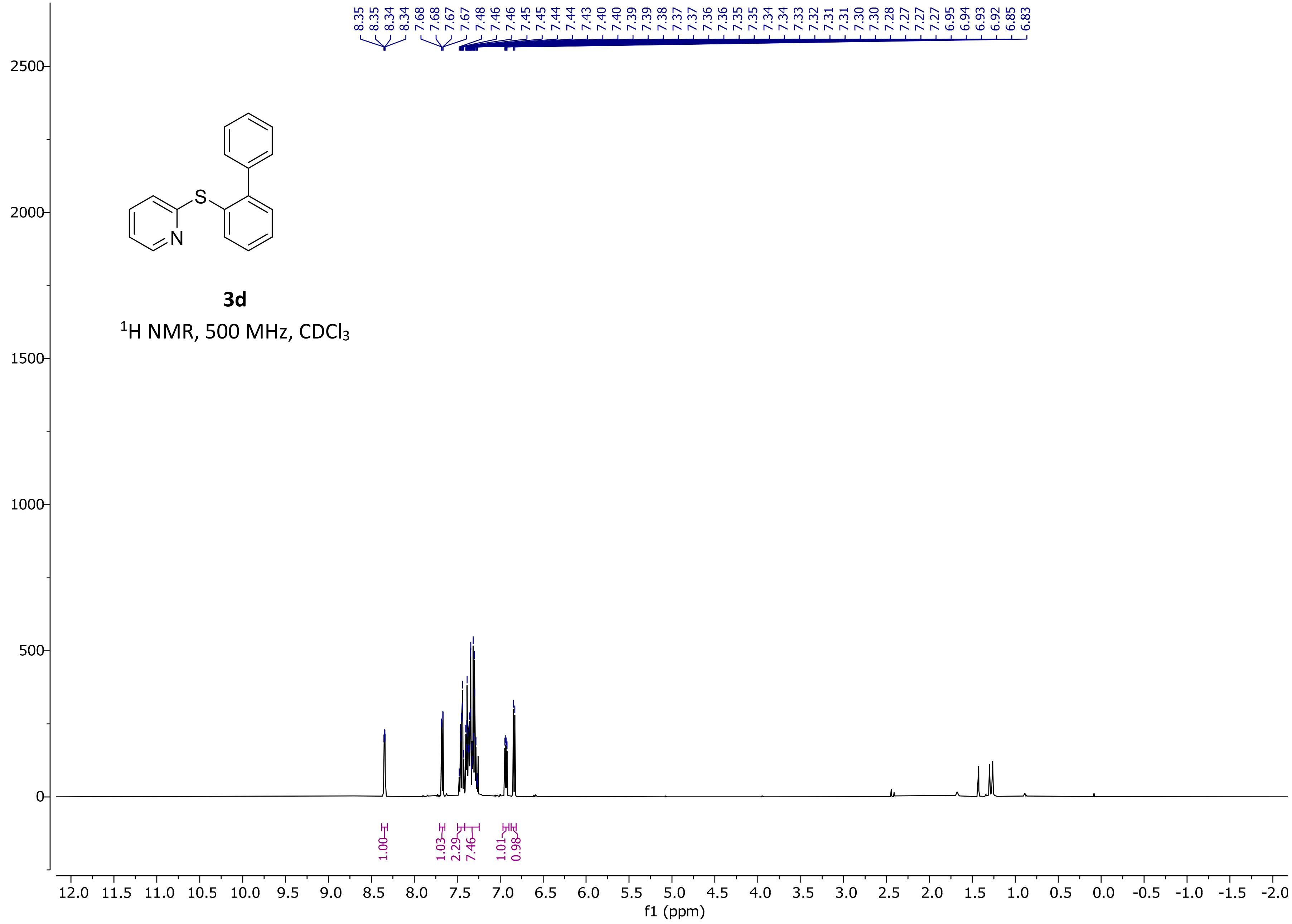
3d
^{1}H NMR, 500 MHz, $CDCl_3$
8.35 8.35 8.34 8.34 7.68 7.68 7.67 7.67 7.48 7.46 7.46 7.45 7.45 7.44 7.44 7.43 7.40 7.40 7.39 7.39 7.38 7.37 7.37 7.36 7.36 7.35 7.35 7.34 7.34 7.33 7.32 7.31 7.31 7.30 7.30 7.28 7.27 7.27 7.27 6.95 6.94 6.93 6.92 6.85 6.83
1.00 1.03 2.29 7.46 1.01 0.98
f1 (ppm)

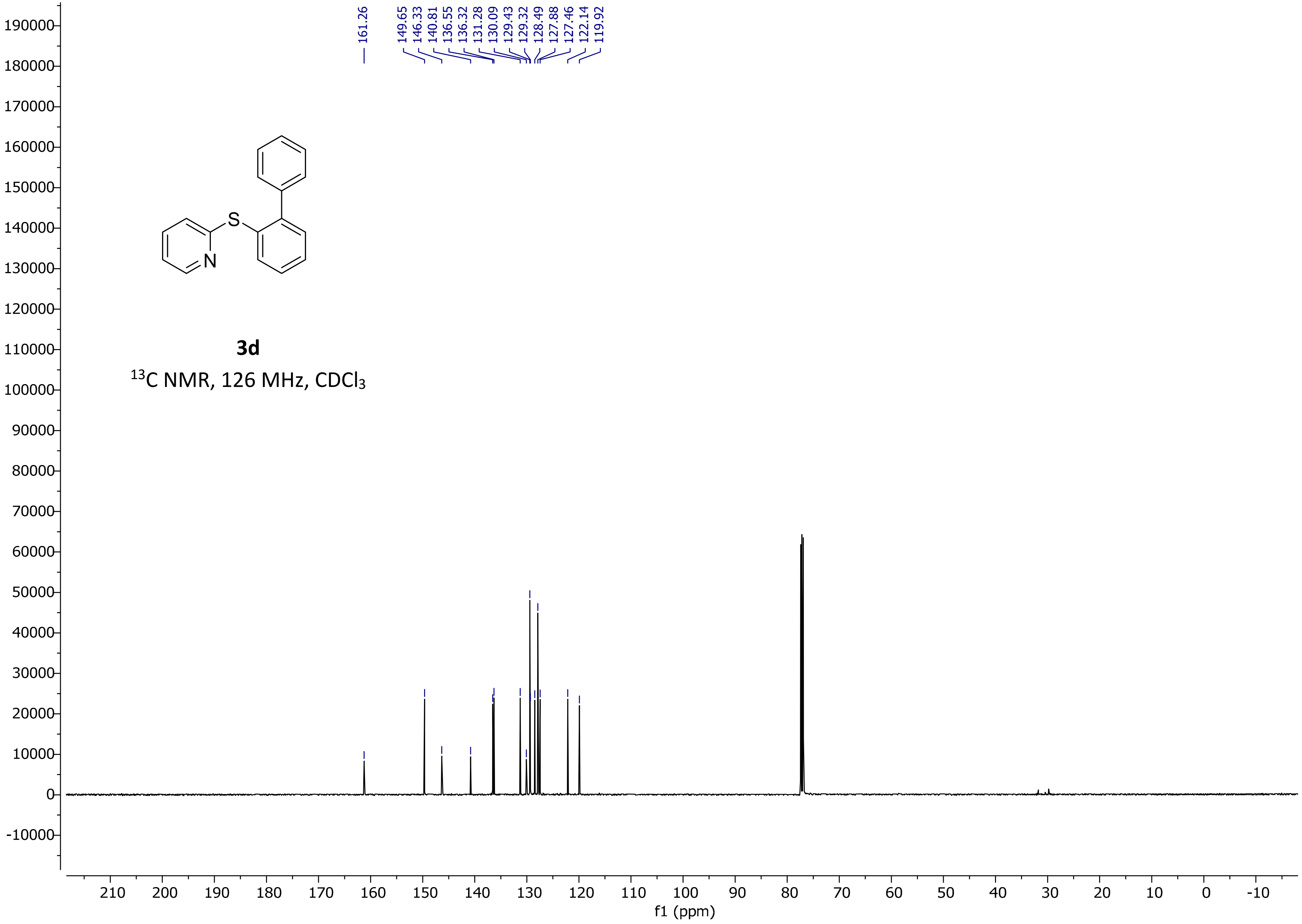

3d

^{13}C NMR, 126 MHz, $CDCl_3$

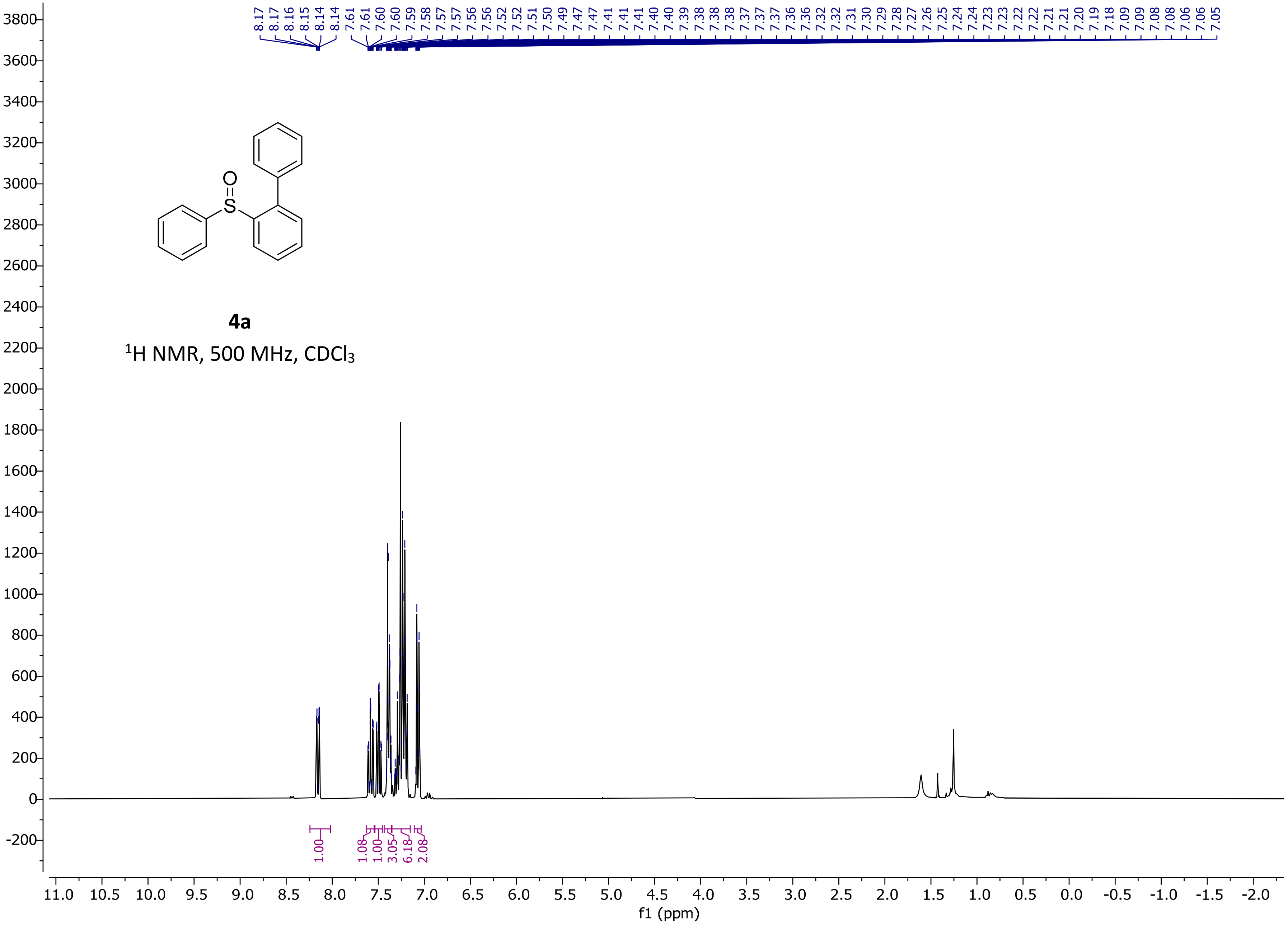
4a
¹H NMR, 500 MHz, CDCl₃
8.17 8.17 8.16 8.15 8.14 8.14 7.61 7.60 7.60 7.59 7.58 7.57 7.57 7.56 7.56 7.52 7.52 7.51 7.50 7.49 7.47 7.47 7.41 7.41 7.41 7.40 7.40 7.39 7.38 7.38 7.38 7.37 7.37 7.36 7.36 7.32 7.32 7.31 7.30 7.29 7.28 7.27 7.26 7.25 7.24 7.24 7.23 7.23 7.22 7.22 7.21 7.21 7.20 7.19 7.18 7.09 7.09 7.08 7.08 7.06 7.06 7.05
1.00
1.08
1.00
3.05
6.18
2.08
f1 (ppm)

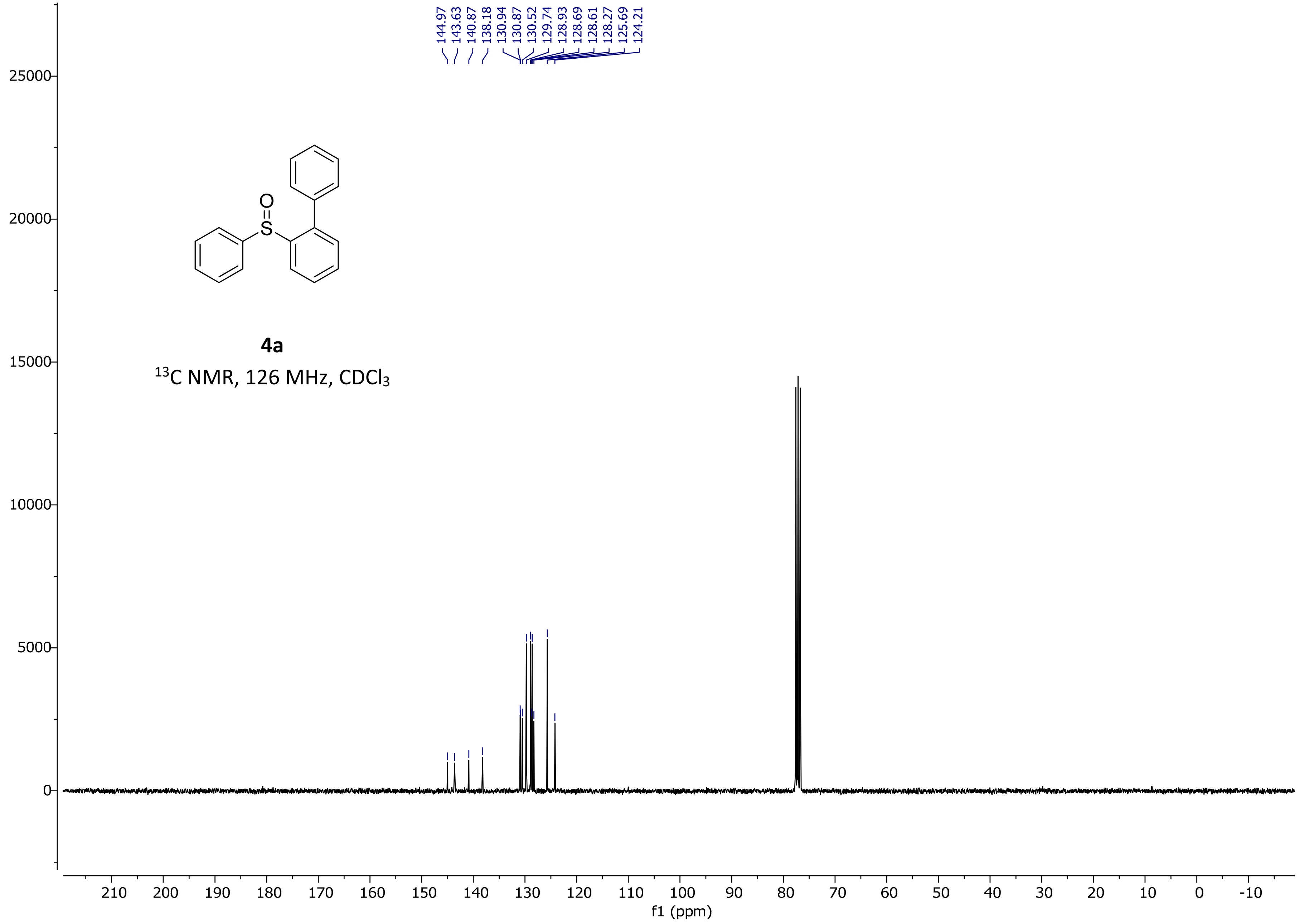
144.97
143.63
140.87
138.18
130.94
130.87
130.52
129.74
128.93
128.69
128.61
128.27
125.69
124.21
O
S
4a
^{13}C NMR, 126 MHz, $CDCl_3$
25000
20000
15000
10000
5000
0
210 200 190 180 170 160 150 140 130 120 110 100 90 80 70 60 50 40 30 20 10 0 -10
f1 (ppm)

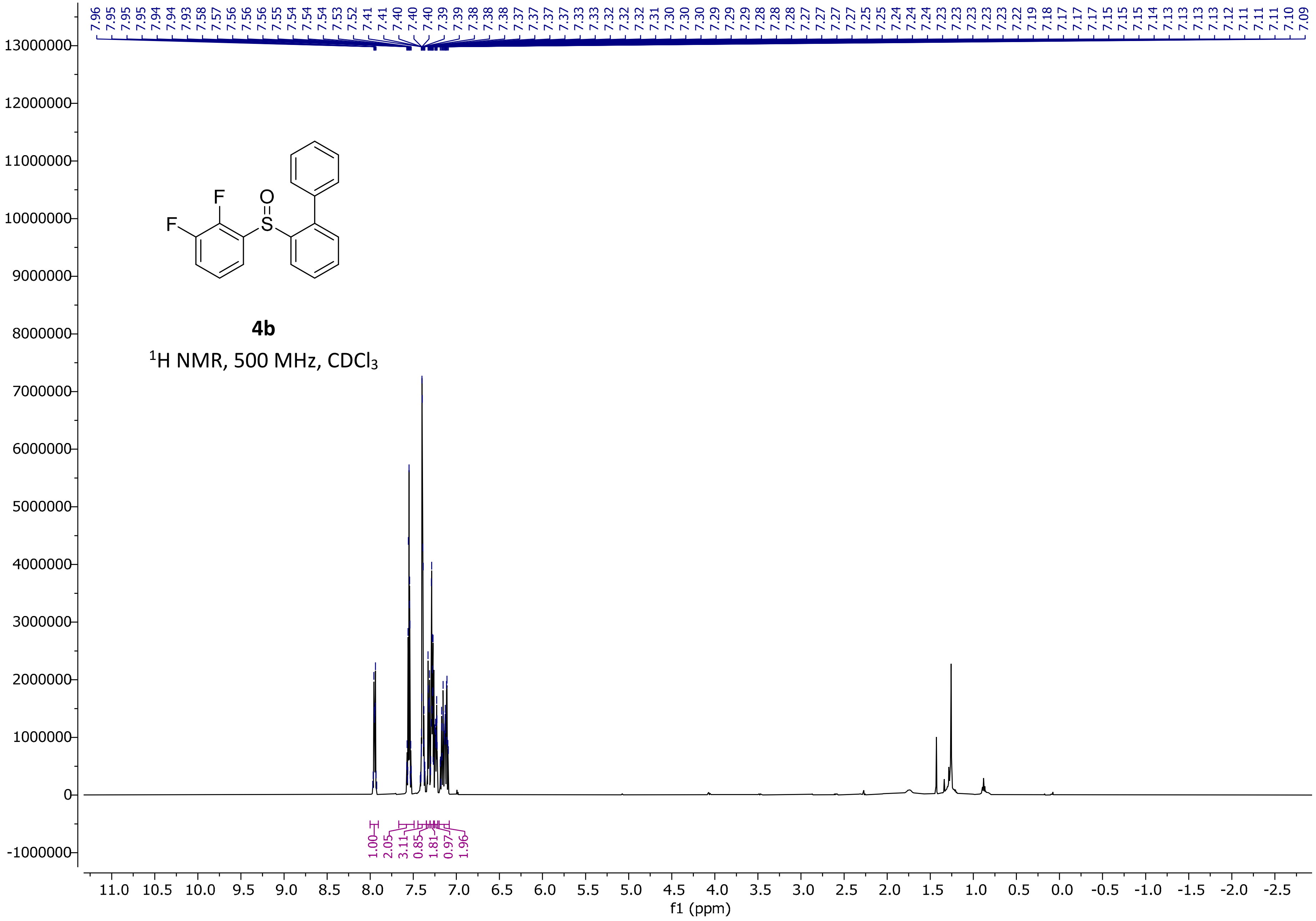

4b
¹H NMR, 500 MHz, CDCl3
f1 (ppm)

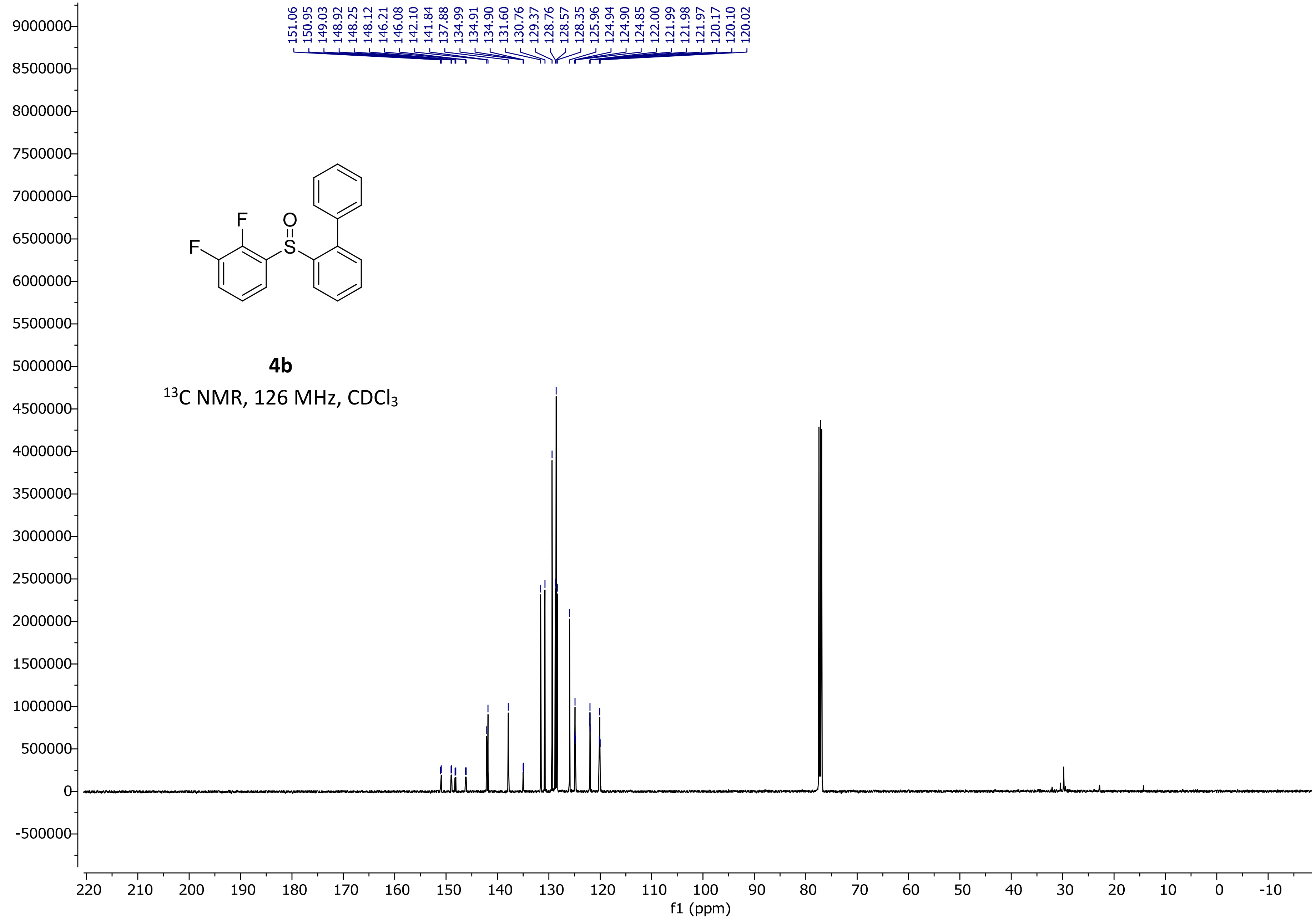
151.06
150.95
149.03
148.92
148.25
148.12
146.21
146.08
142.10
141.84
137.88
134.99
134.91
134.90
131.60
130.76
129.37
128.76
128.57
128.35
125.96
124.94
124.90
124.85
122.00
121.99
121.98
121.97
120.17
120.10
120.02
F
F
O
S
4b
^{13}C NMR, 126 MHz, CDCl$_3$
f1 (ppm)

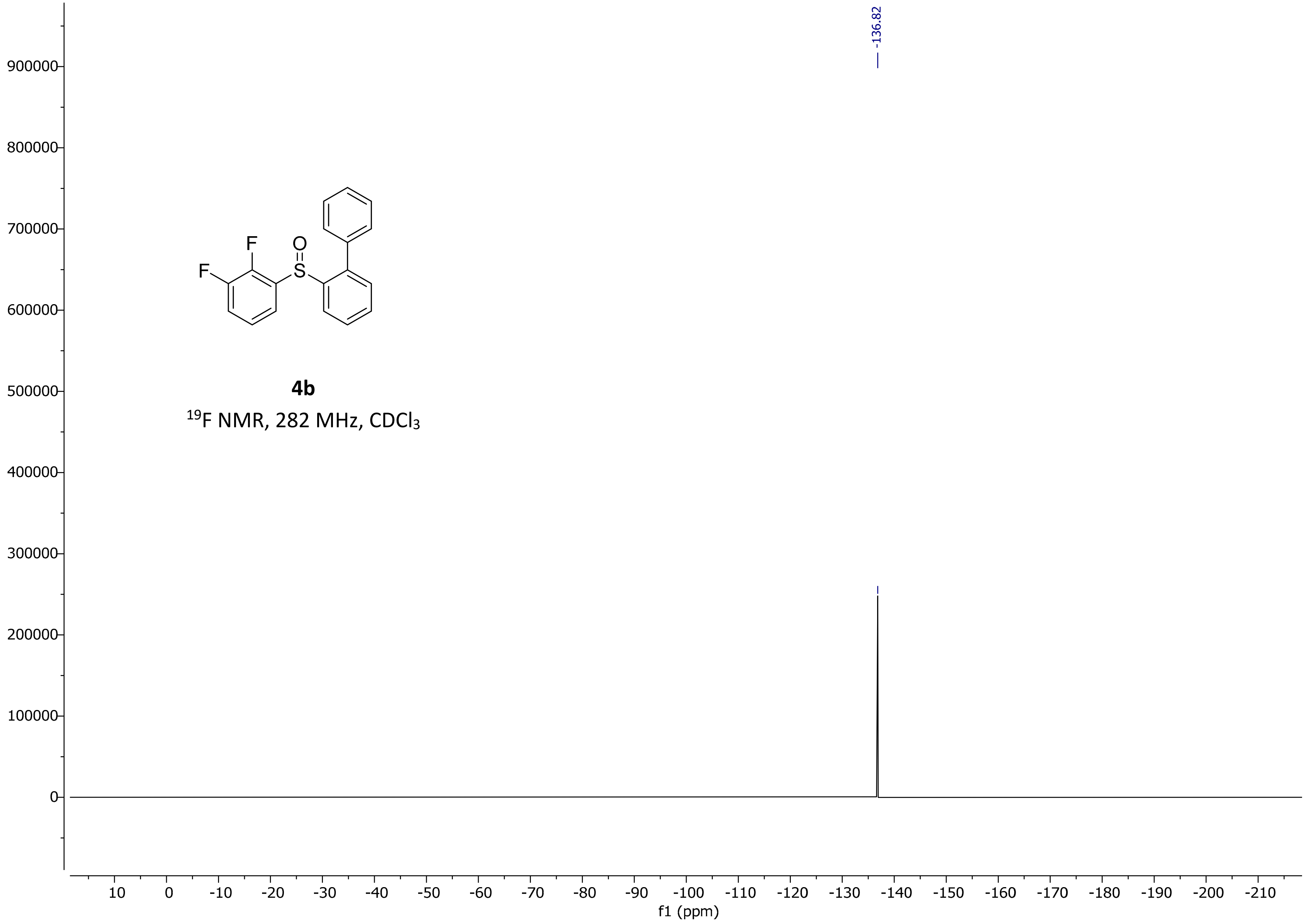
-136.82
F
F
O
S
4b
^{19}F NMR, 282 MHz, $CDCl_3$
900000
800000
700000
600000
500000
400000
300000
200000
100000
0
10 0 -10 -20 -30 -40 -50 -60 -70 -80 -90 -100 -110 -120 -130 -140 -150 -160 -170 -180 -190 -200 -210
f1 (ppm)

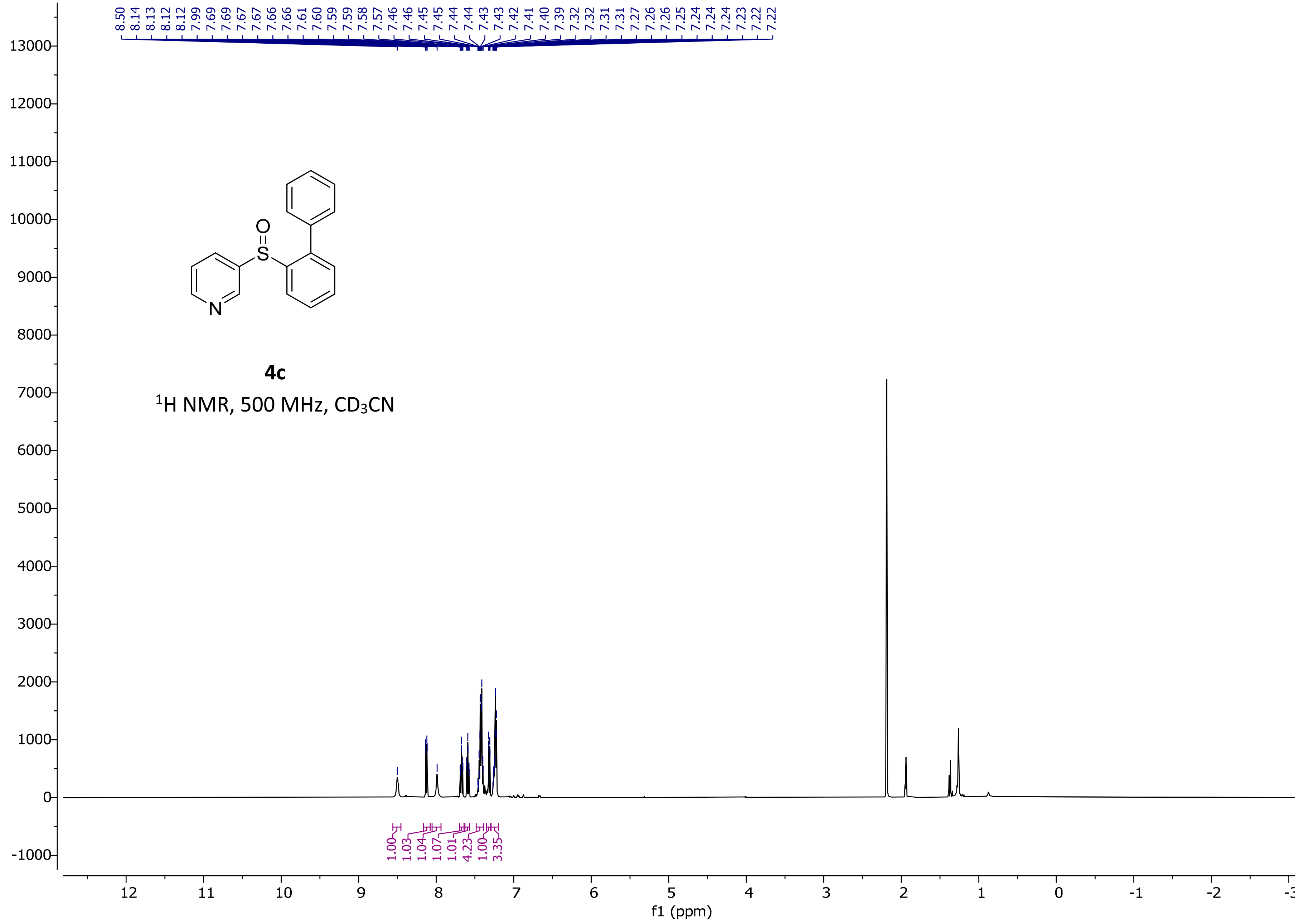
8.50 8.14 8.13 8.12 8.12 7.99 7.69 7.69 7.67 7.67 7.66 7.66 7.61 7.60 7.59 7.59 7.58 7.57 7.46 7.46 7.45 7.45 7.44 7.44 7.43 7.43 7.42 7.41 7.40 7.39 7.32 7.32 7.31 7.31 7.27 7.26 7.26 7.25 7.24 7.24 7.24 7.23 7.22 7.22
O
S
N
4c
1H NMR, 500 MHz, CD3CN
1.00 1.03 1.04 1.07 1.01 4.23 1.00 3.35
f1 (ppm)

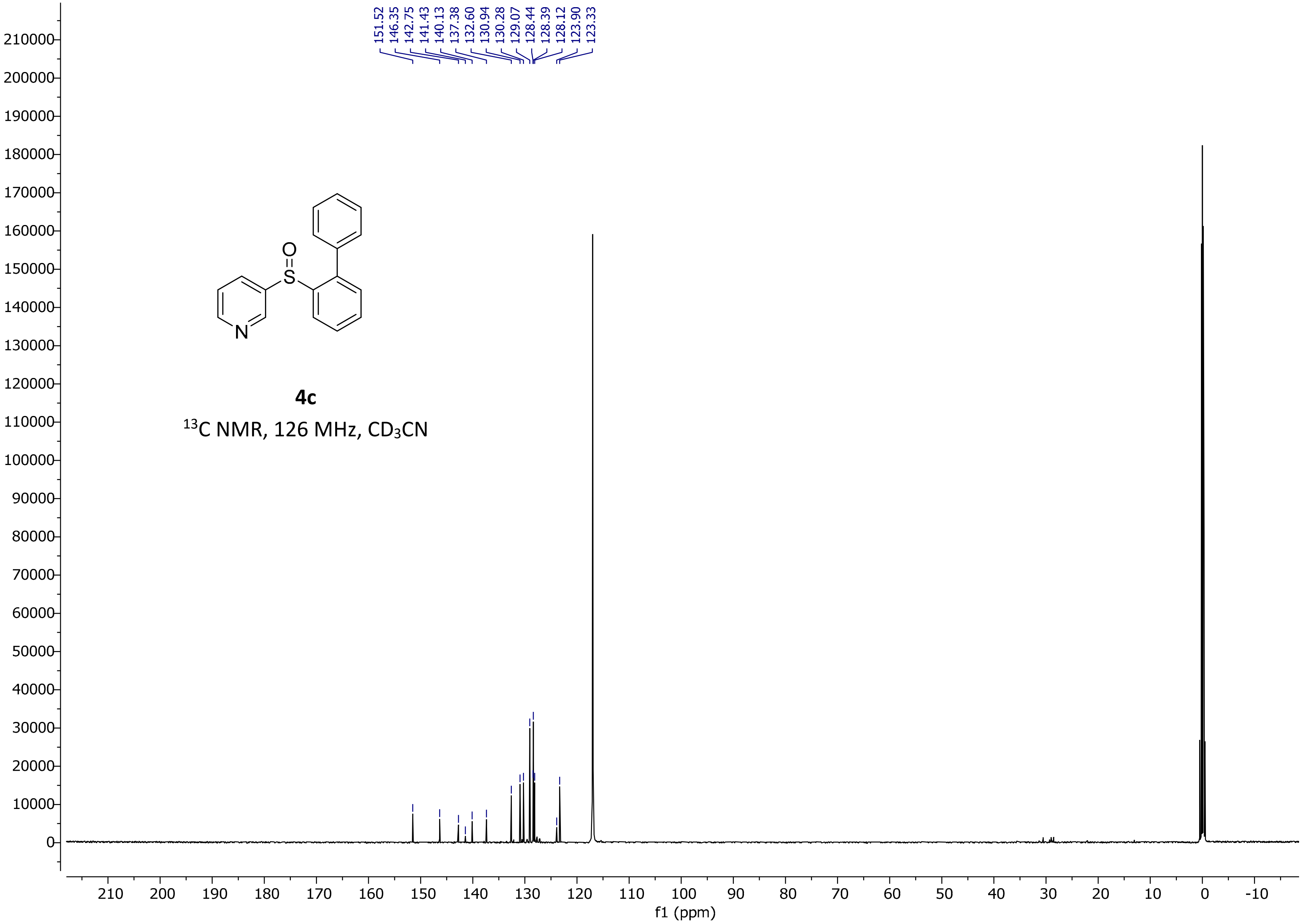
151.52
146.35
142.75
141.43
140.13
137.38
132.60
130.94
130.28
129.07
128.44
128.39
128.12
123.90
123.33
O
S
N
4c
^{13}C NMR, 126 MHz, CD_3CN
f1 (ppm)

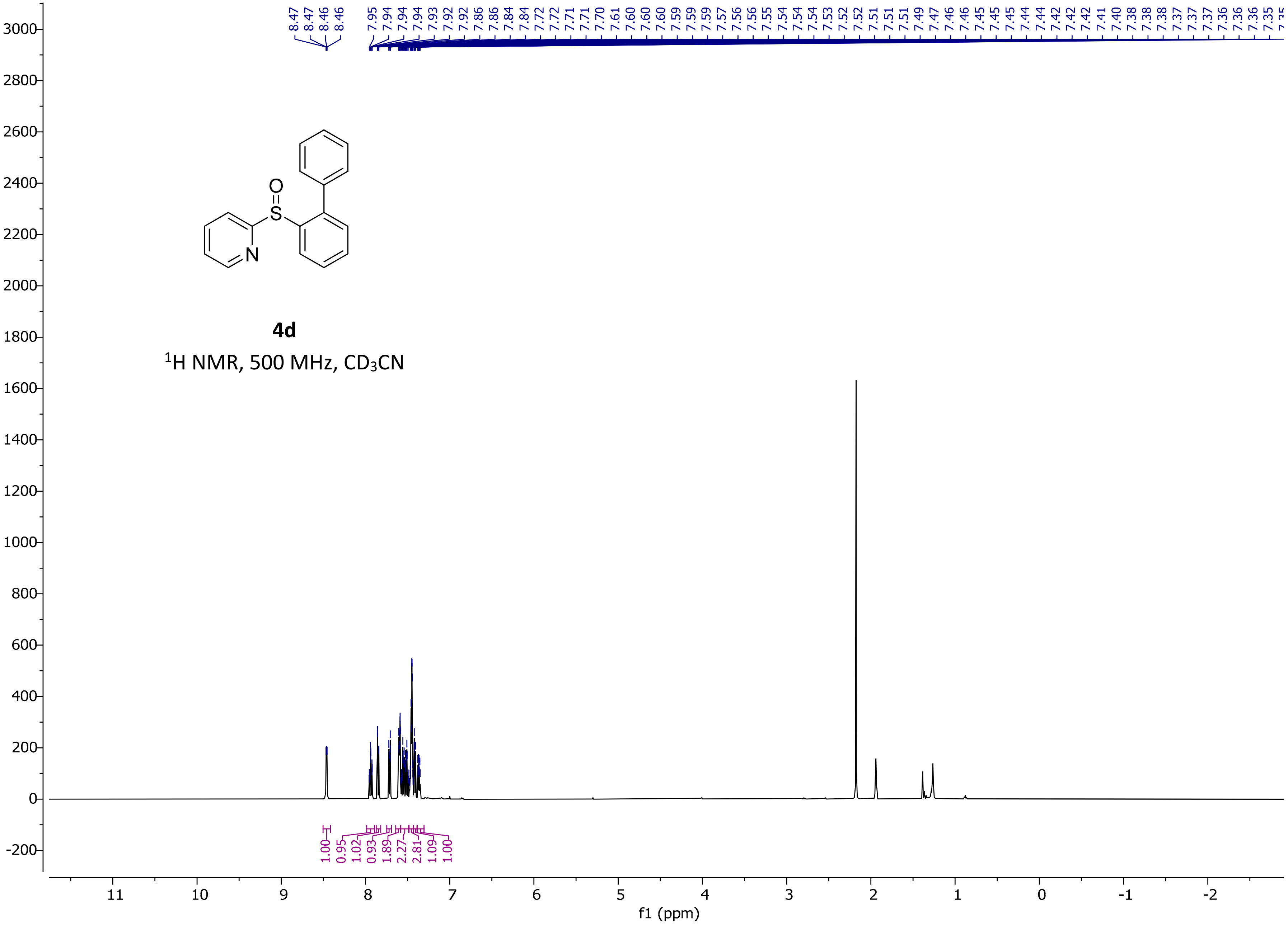

4d

^1H NMR, 500 MHz, CD_3CN

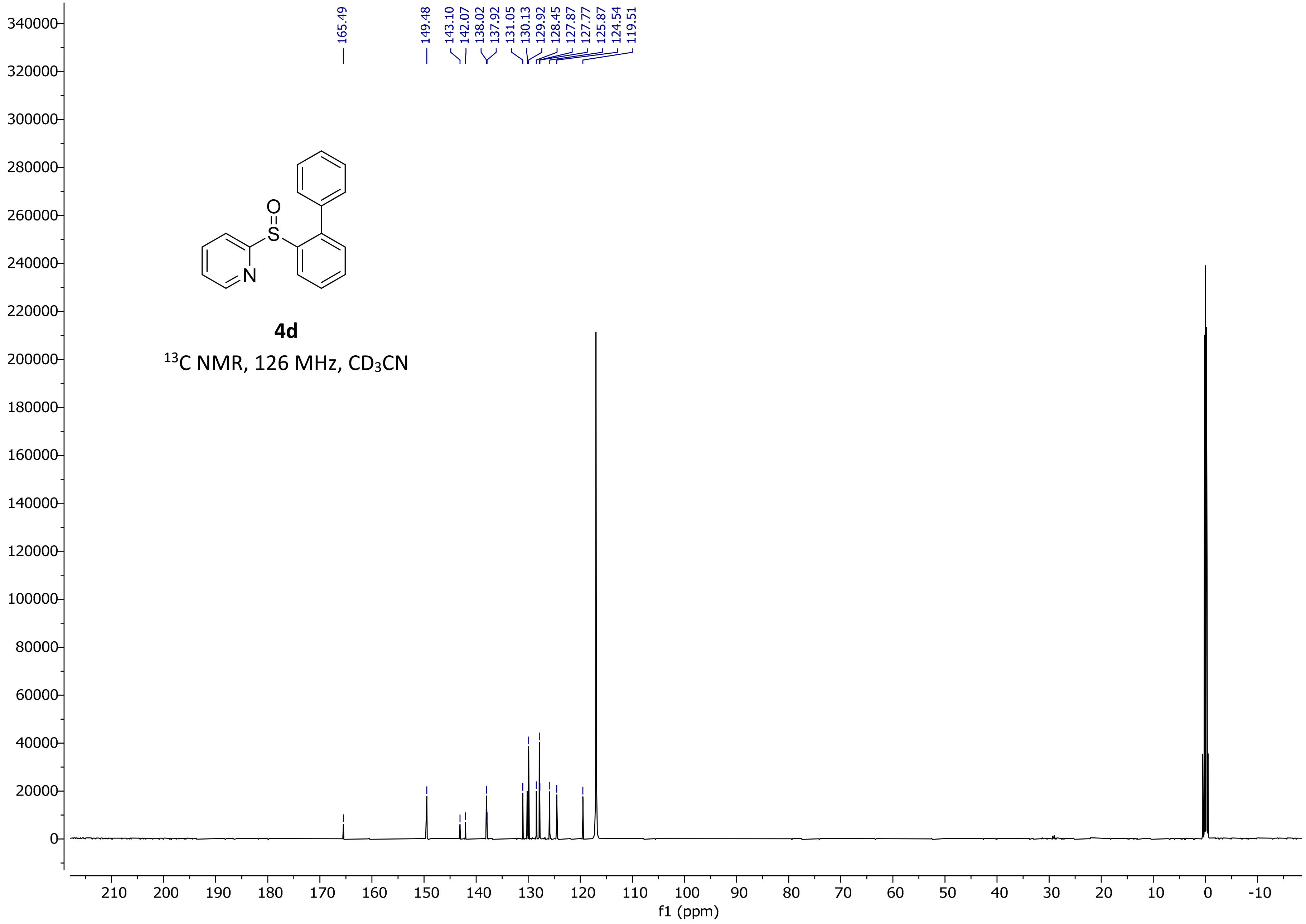
165.49
149.48
143.10
142.07
138.02
137.92
131.05
130.13
129.92
128.45
127.87
127.77
125.87
124.54
119.51
O
S
N
4d
^{13}C NMR, 126 MHz, CD_3CN
f1 (ppm)

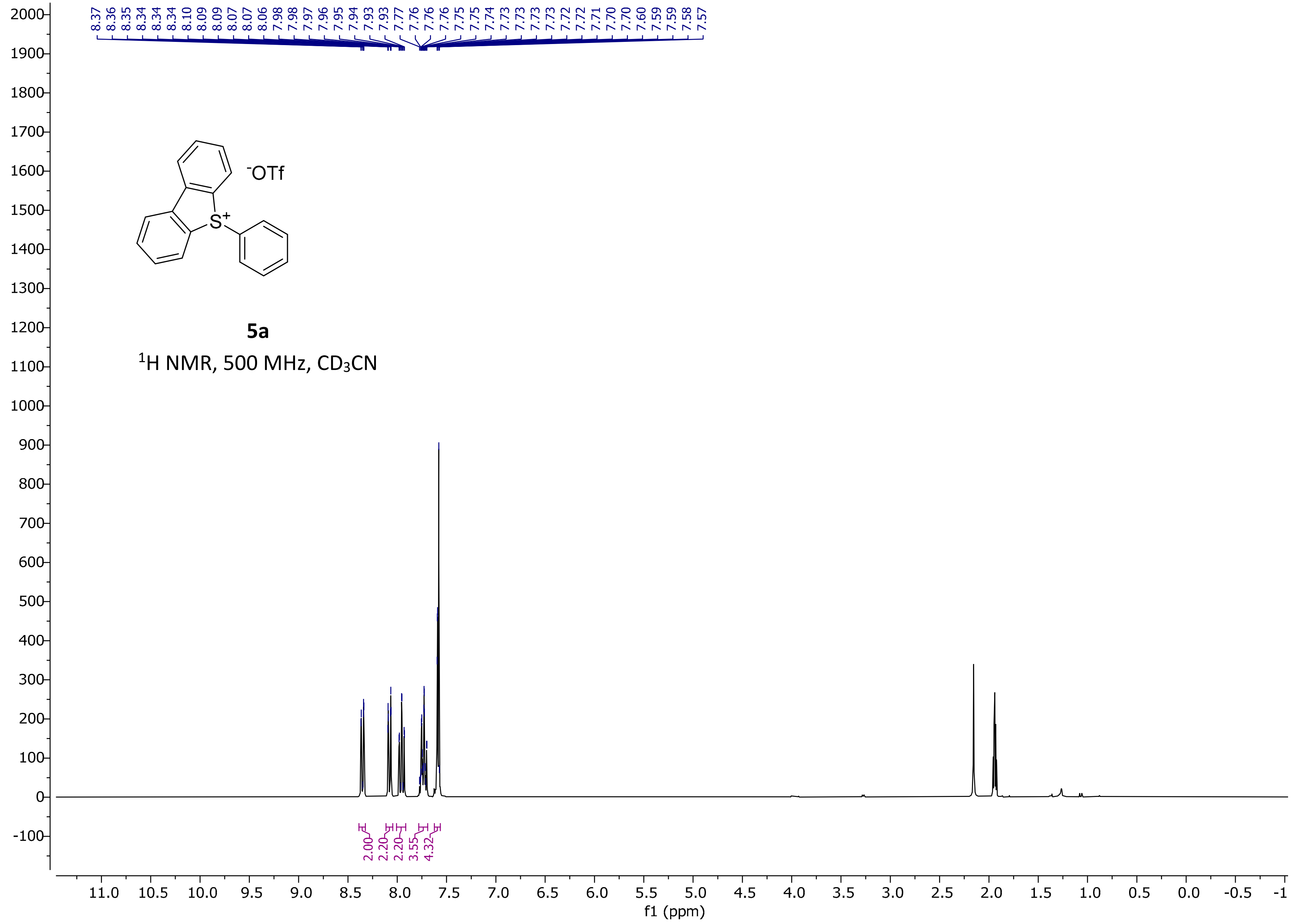
8.37 8.36 8.35 8.34 8.34 8.10 8.09 8.09 8.07 8.07 8.06 7.98 7.97 7.96 7.95 7.94 7.93 7.93 7.77 7.76 7.76 7.76 7.75 7.75 7.74 7.73 7.73 7.73 7.73 7.72 7.72 7.71 7.70 7.70 7.60 7.59 7.59 7.58 7.57
-OTf
S+
5a
^{1}H NMR, 500 MHz, CD_3CN
2.00 2.20 2.20 3.55 4.32
f1 (ppm)

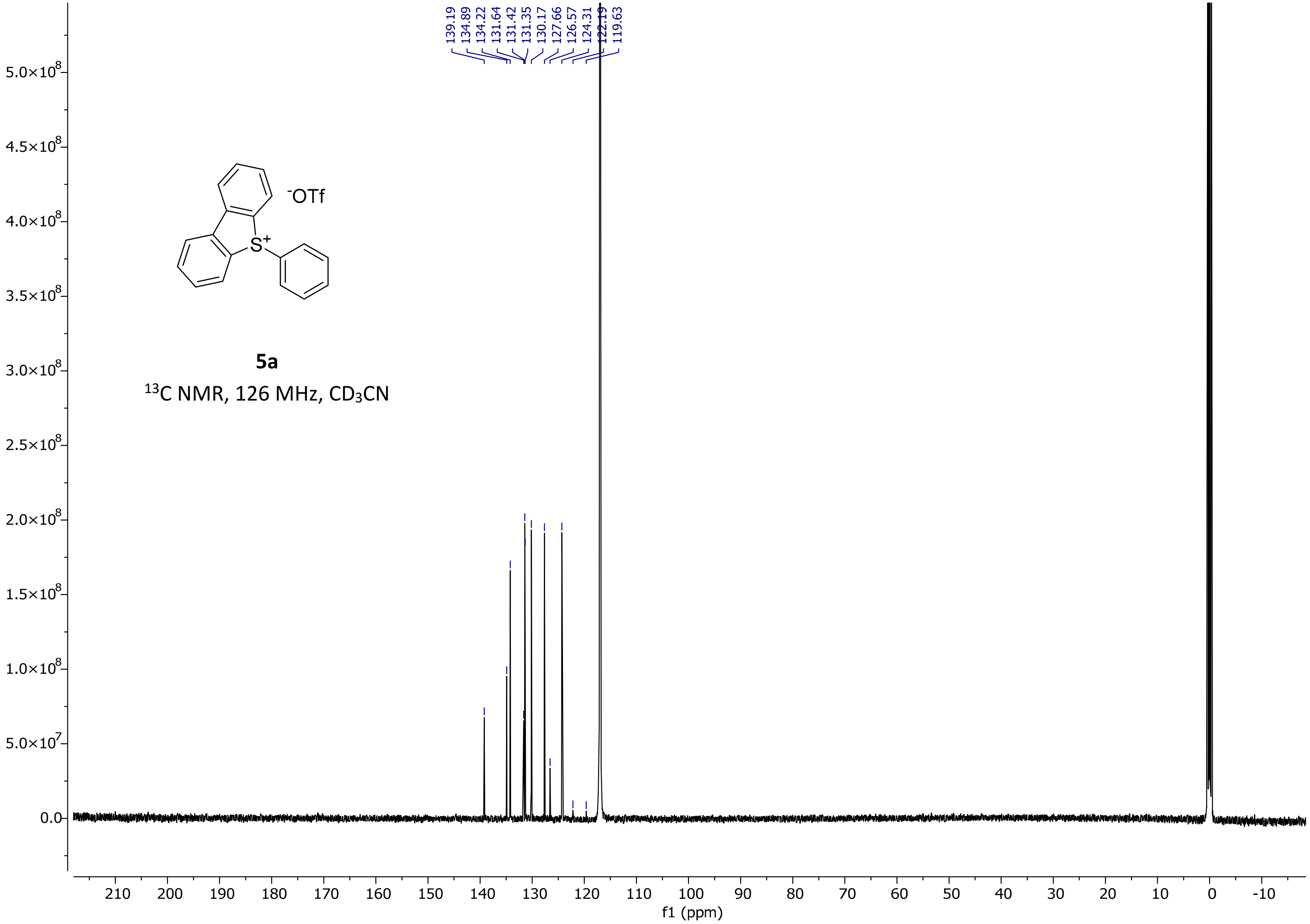

5a

^{13}C NMR, 126 MHz, CD_3CN

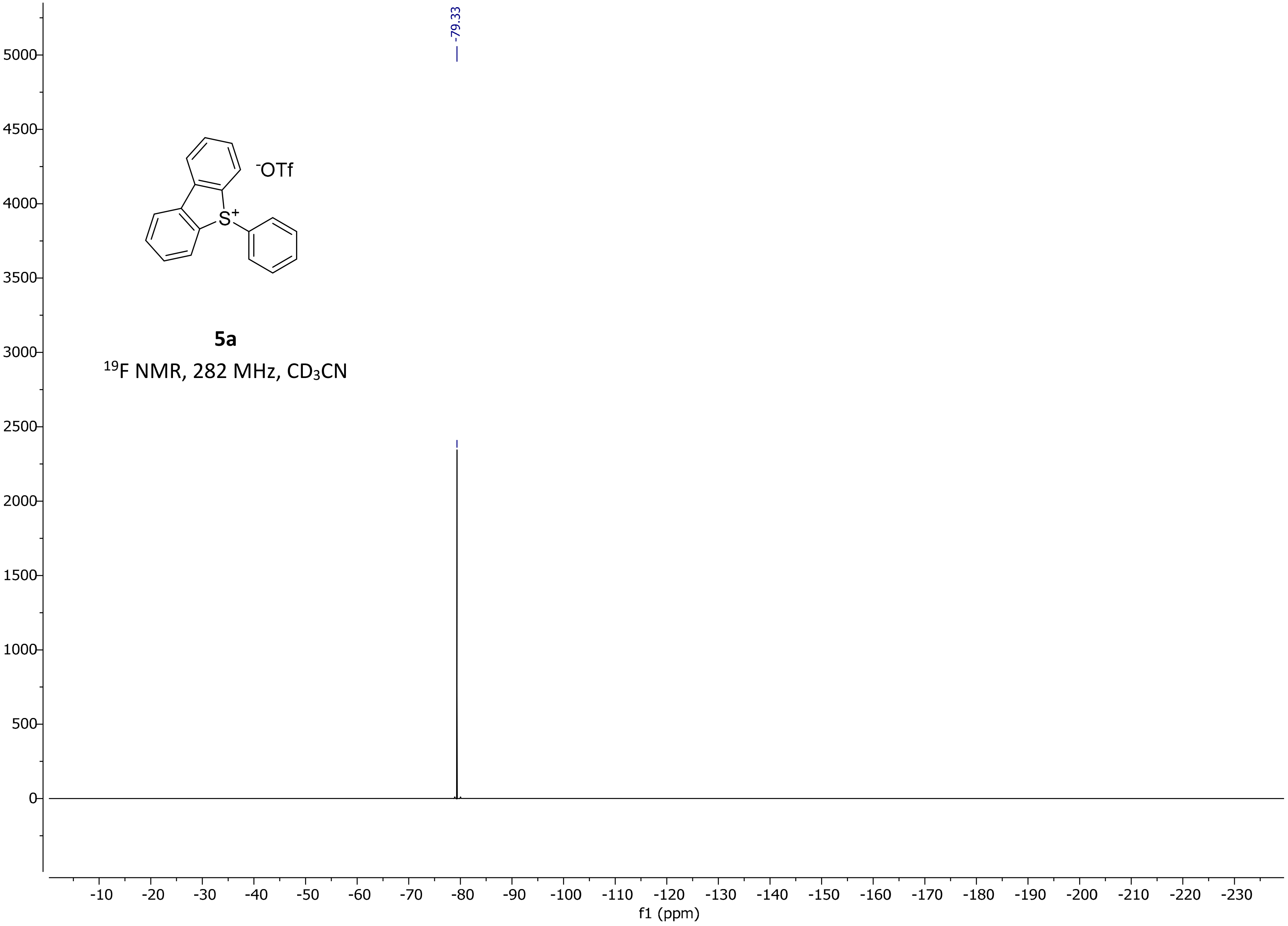

5a

^{19}F NMR, 282 MHz, CD_3CN

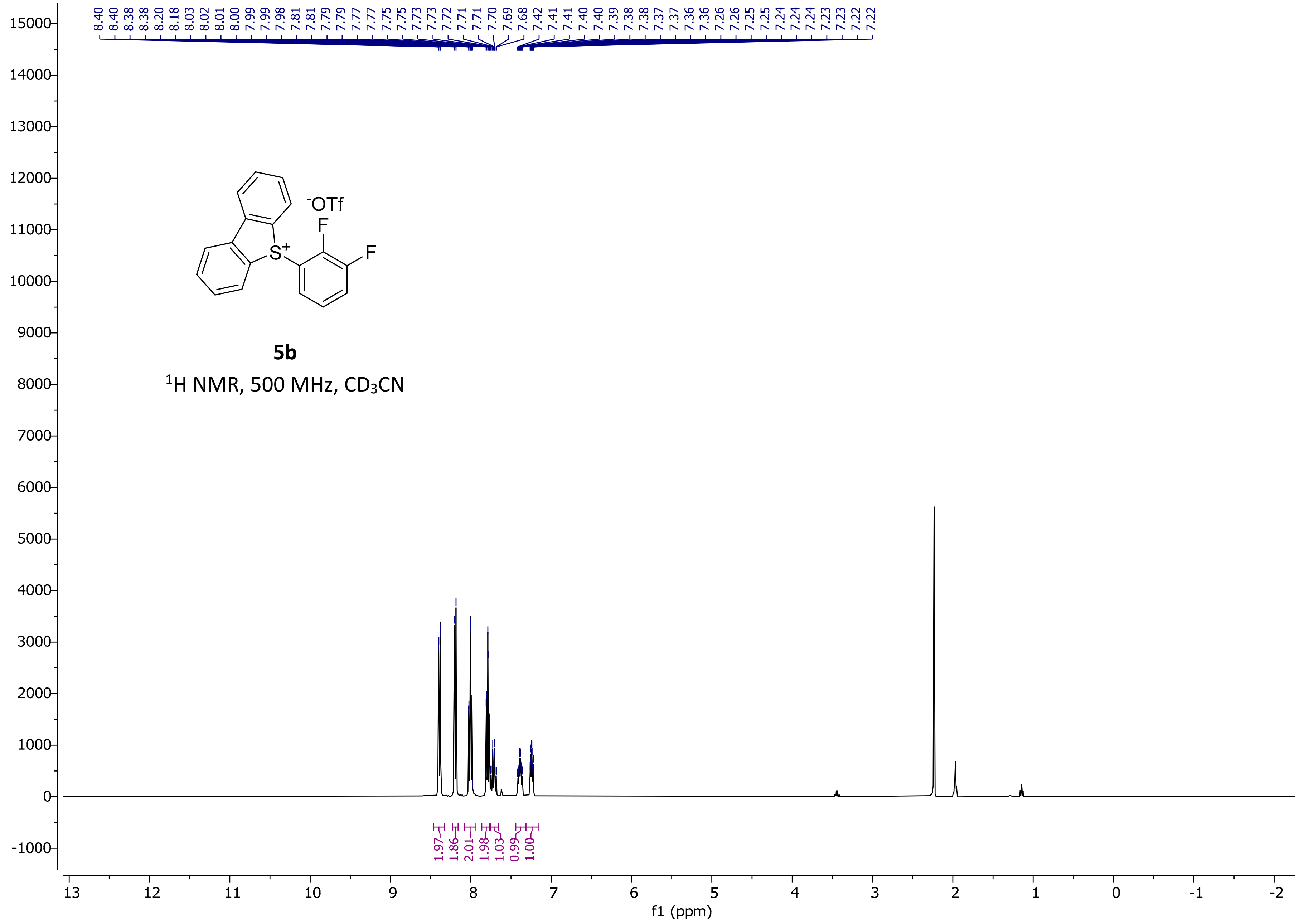
-OTf
F
F
S+
5b
1H NMR, 500 MHz, CD3CN
8.40 8.40 8.38 8.38 8.20 8.18 8.03 8.02 8.01 8.00 7.99 7.99 7.98 7.81 7.81 7.79 7.79 7.77 7.77 7.75 7.75 7.73 7.73 7.72 7.71 7.71 7.70 7.69 7.68 7.42 7.41 7.41 7.40 7.40 7.39 7.38 7.38 7.37 7.37 7.36 7.36 7.26 7.26 7.25 7.25 7.24 7.24 7.24 7.23 7.23 7.22 7.22
1.97 1.86 2.01 1.98 1.03 0.99 1.00
f1 (ppm)

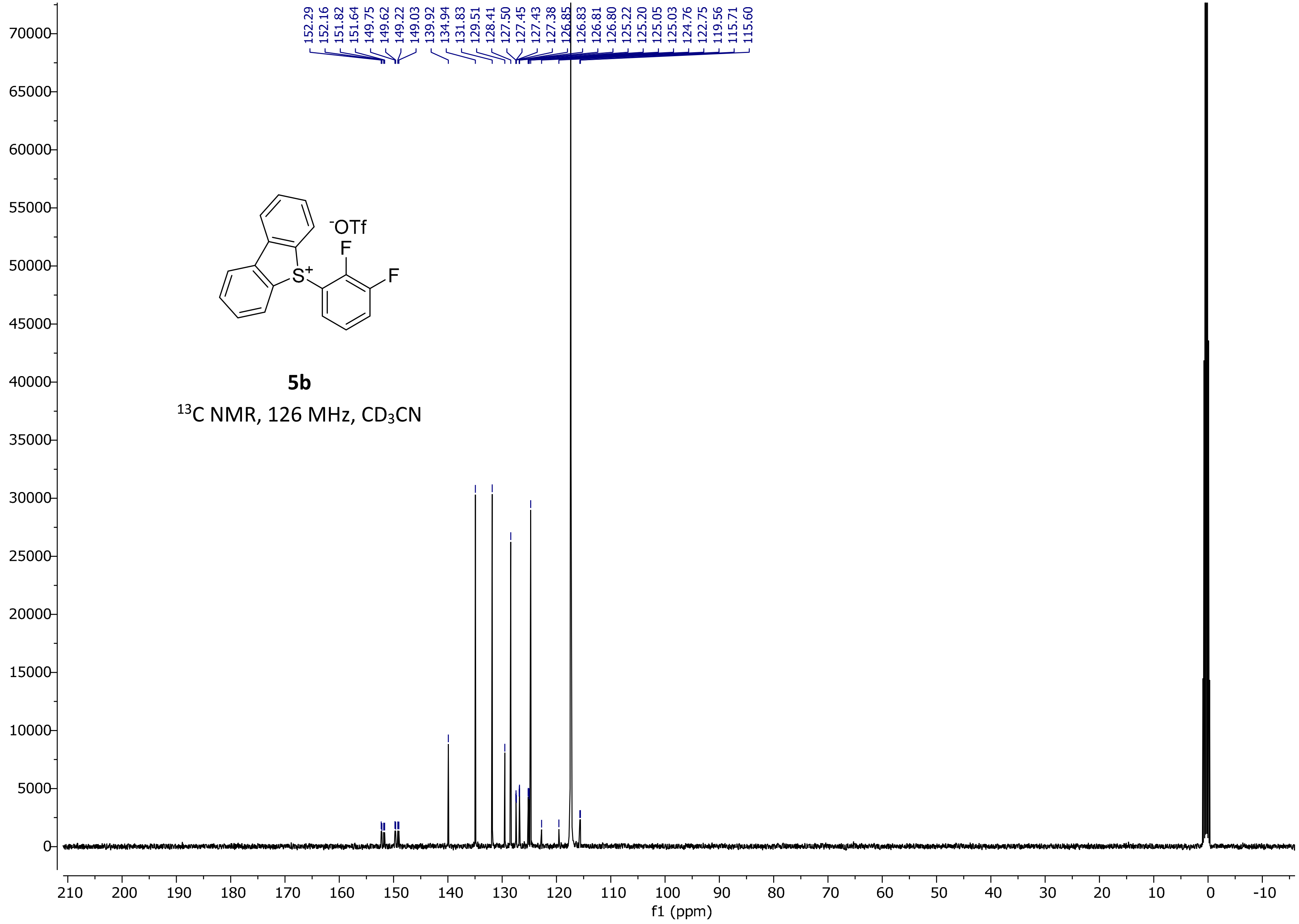
152.29
152.16
151.82
151.64
149.75
149.62
149.22
149.03
139.92
134.94
131.83
129.51
128.41
127.50
127.45
127.43
127.38
126.85
126.83
126.81
126.80
125.22
125.20
125.05
125.03
124.76
122.75
119.56
115.71
115.60
-OTf
F
F
S+
5b
13C NMR, 126 MHz, CD3CN
f1 (ppm)

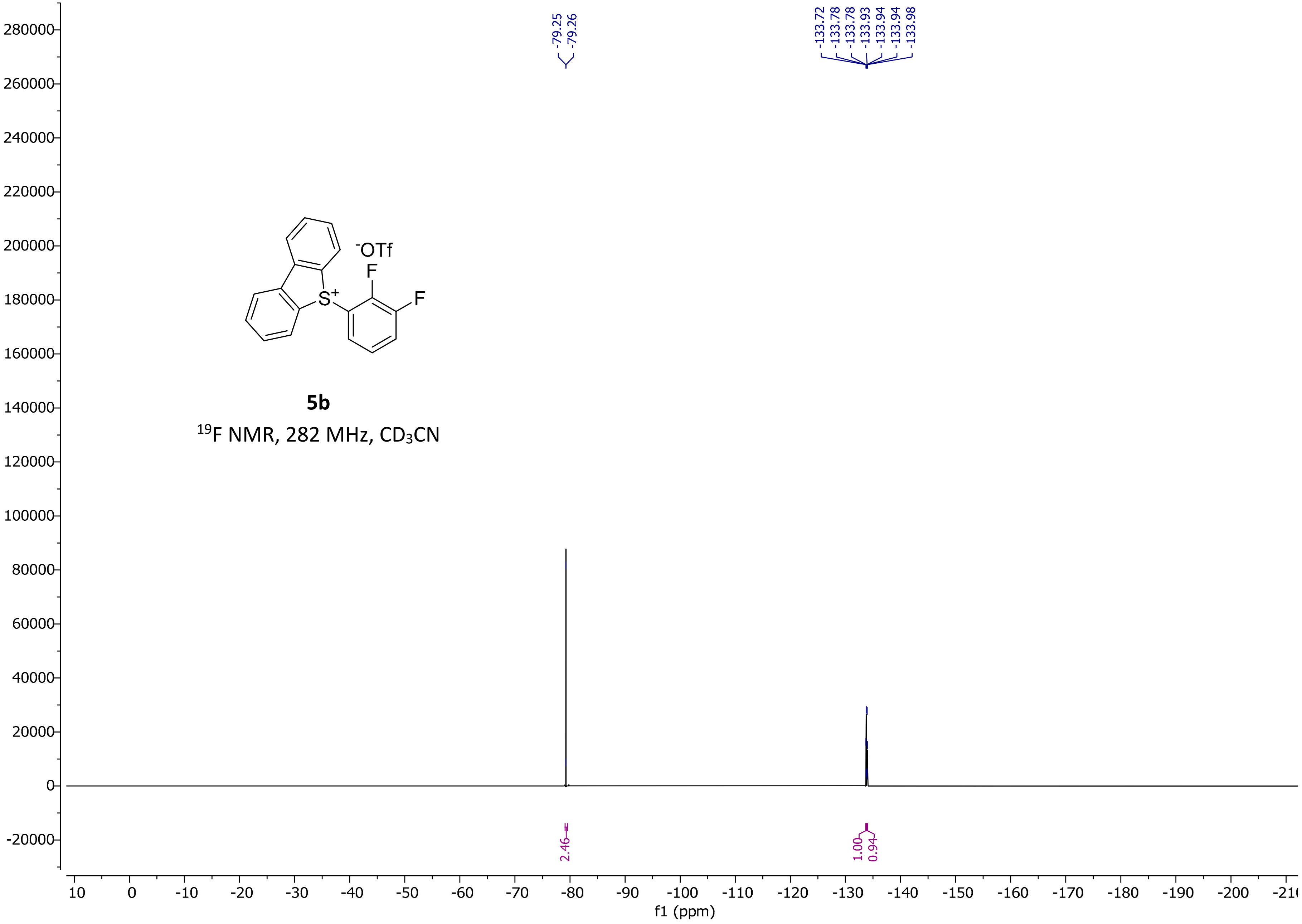
-79.25
-79.26
-133.72
-133.78
-133.78
-133.93
-133.94
-133.94
-133.98
⁻OTf
F
F
S+
5b
^{19}F NMR, 282 MHz, CD_3CN
2.46
1.00
0.94
f1 (ppm)

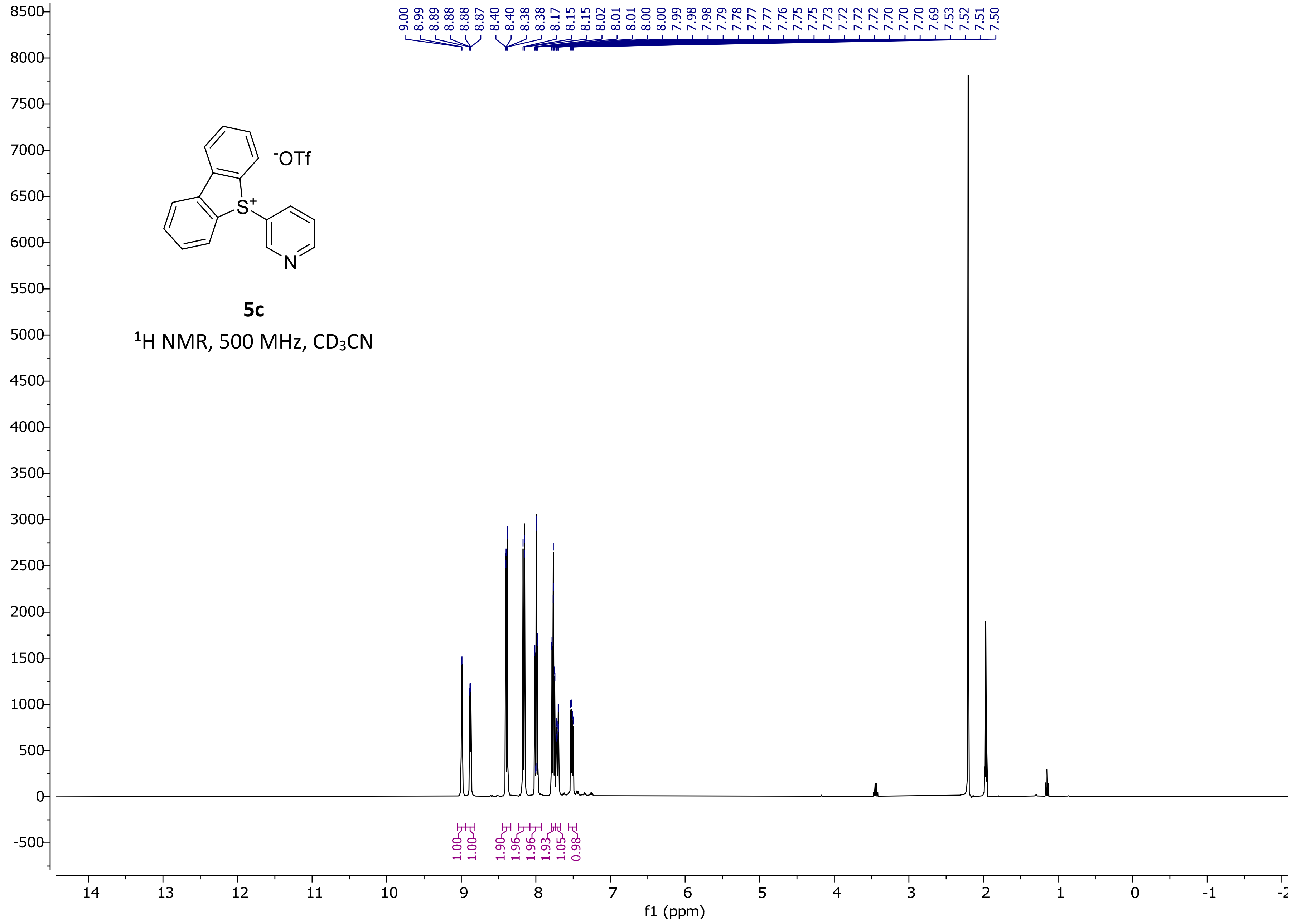
-OTf
S+
N
5c
1H NMR, 500 MHz, CD3CN
f1 (ppm)

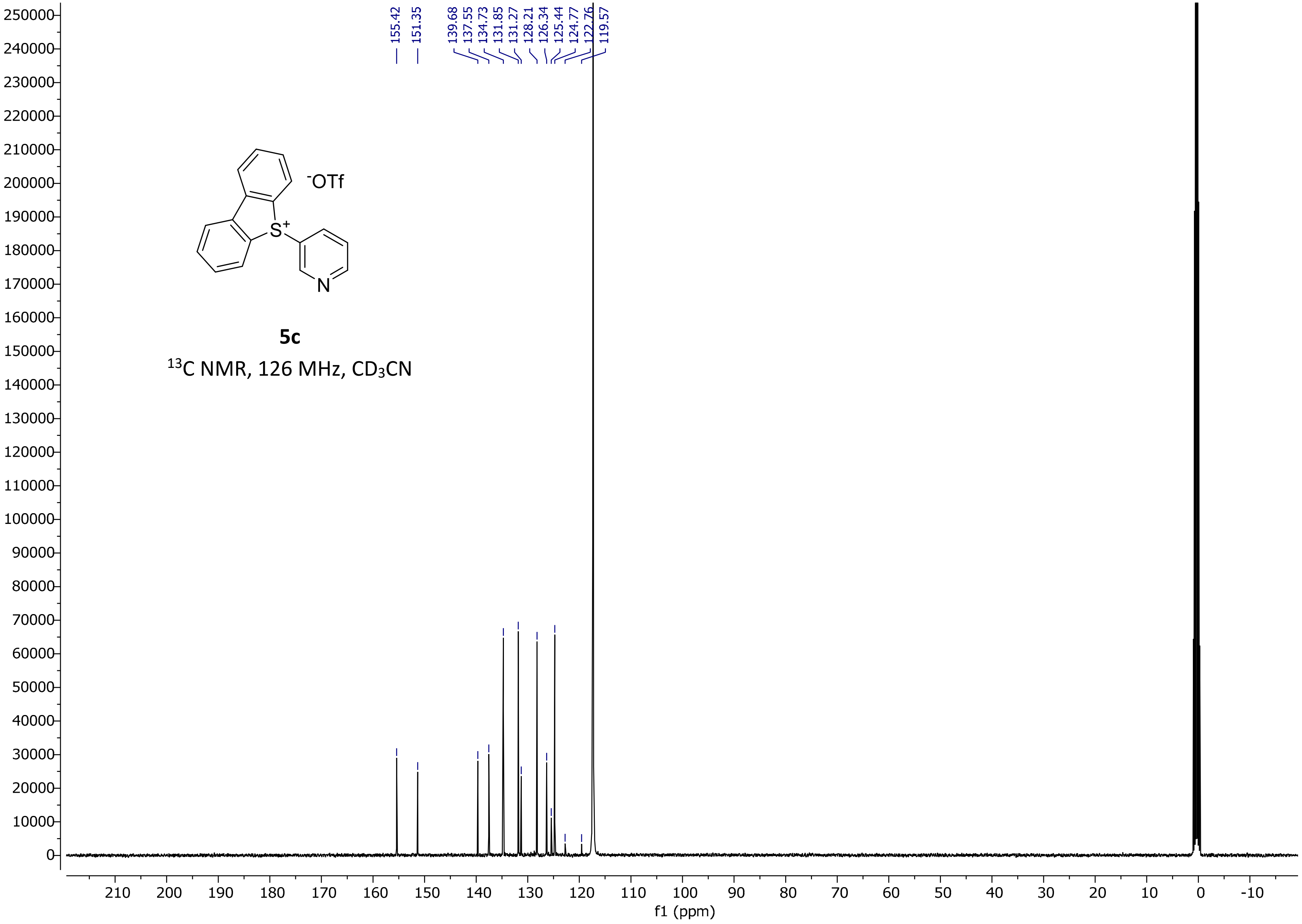

155.42
151.35
139.68
137.55
134.73
131.85
131.27
128.21
126.34
125.44
124.77
122.76
119.57
-OTf
S+
N
5c
^{13}C NMR, 126 MHz, CD_3CN
f1 (ppm)

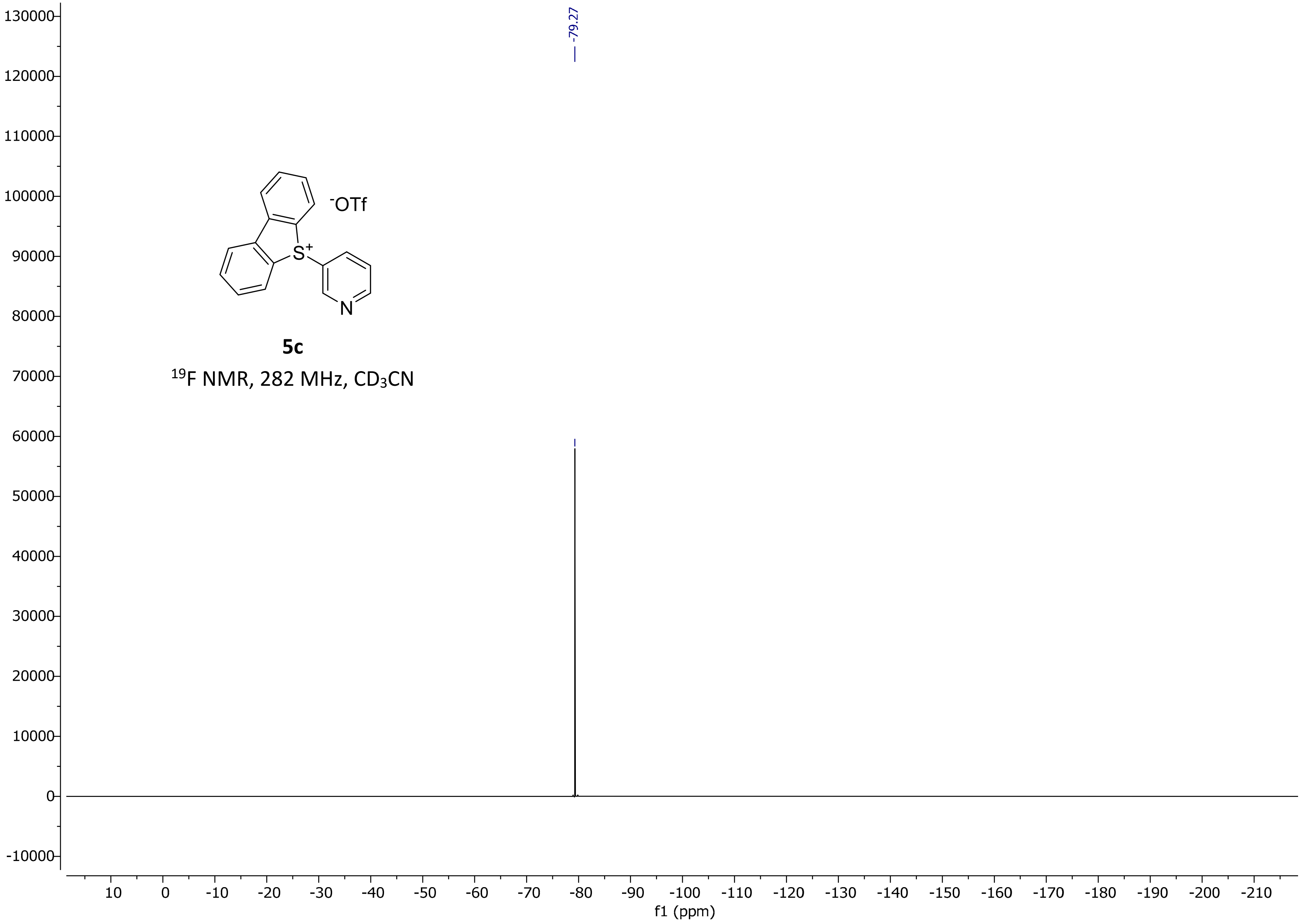

-79.27
-OTf
S+
N
5c
19F NMR, 282 MHz, CD3CN
f1 (ppm)

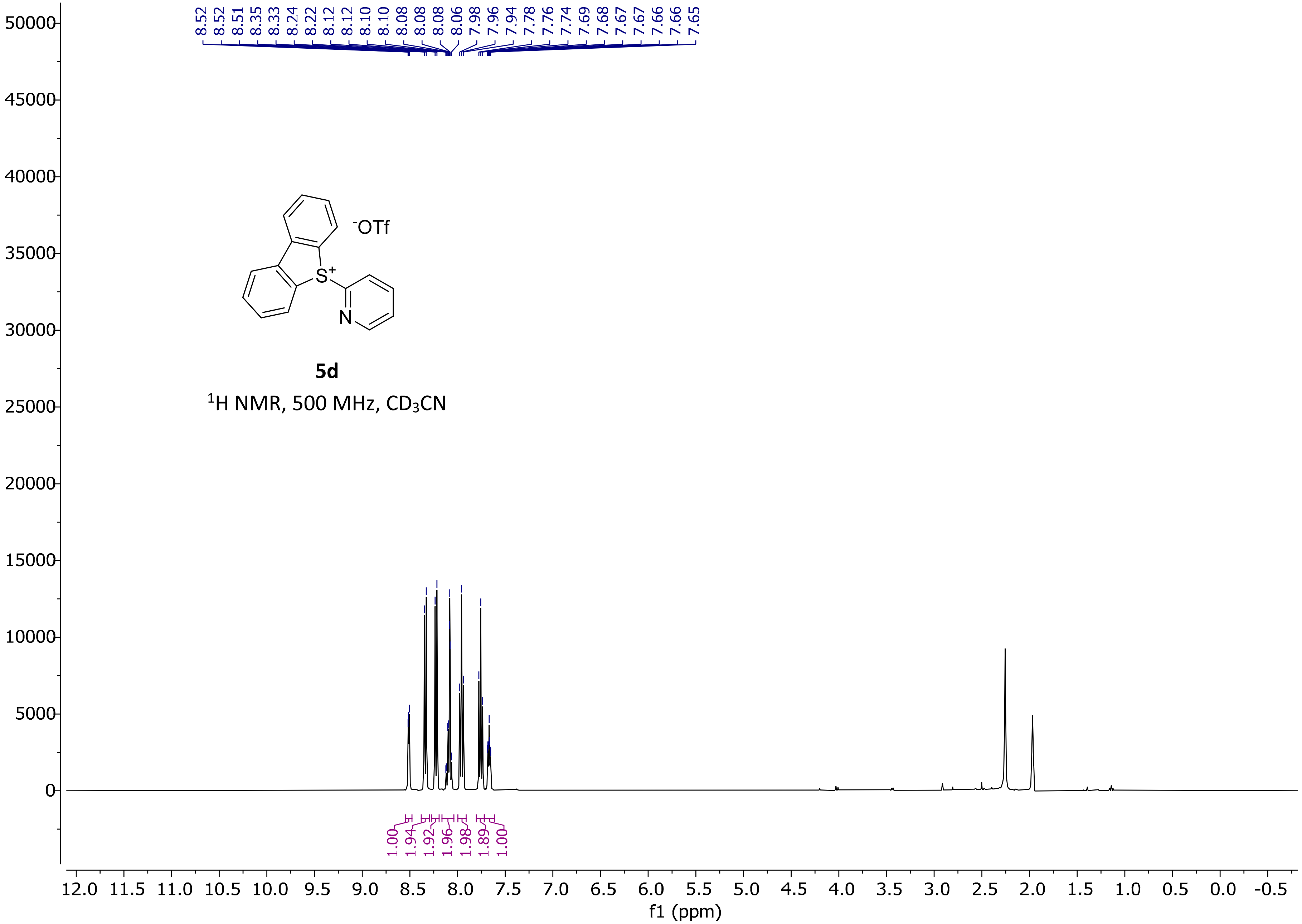

5d

^{1}H NMR, 500 MHz, CD_3CN

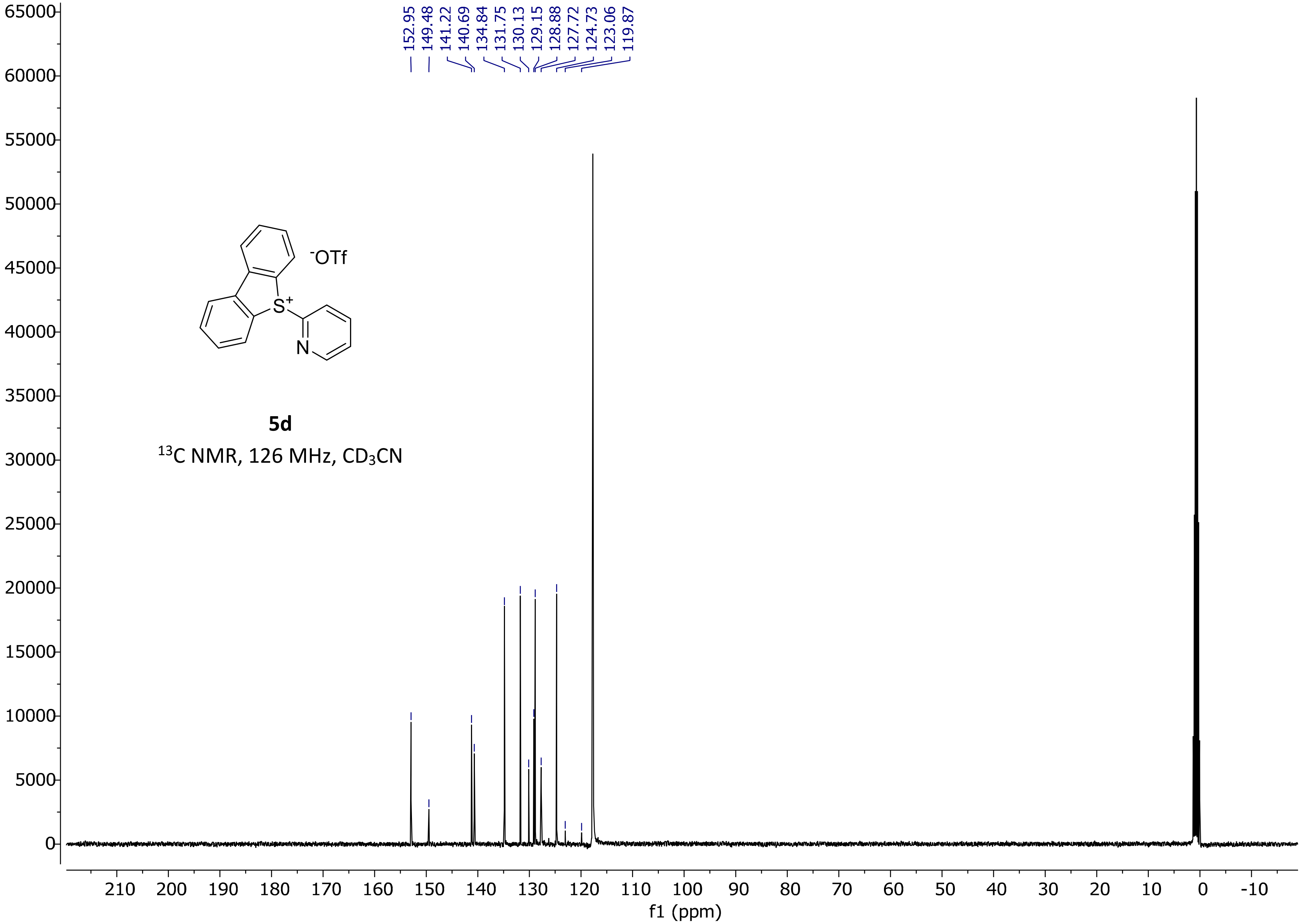

5d

^{13}C NMR, 126 MHz, CD_3CN

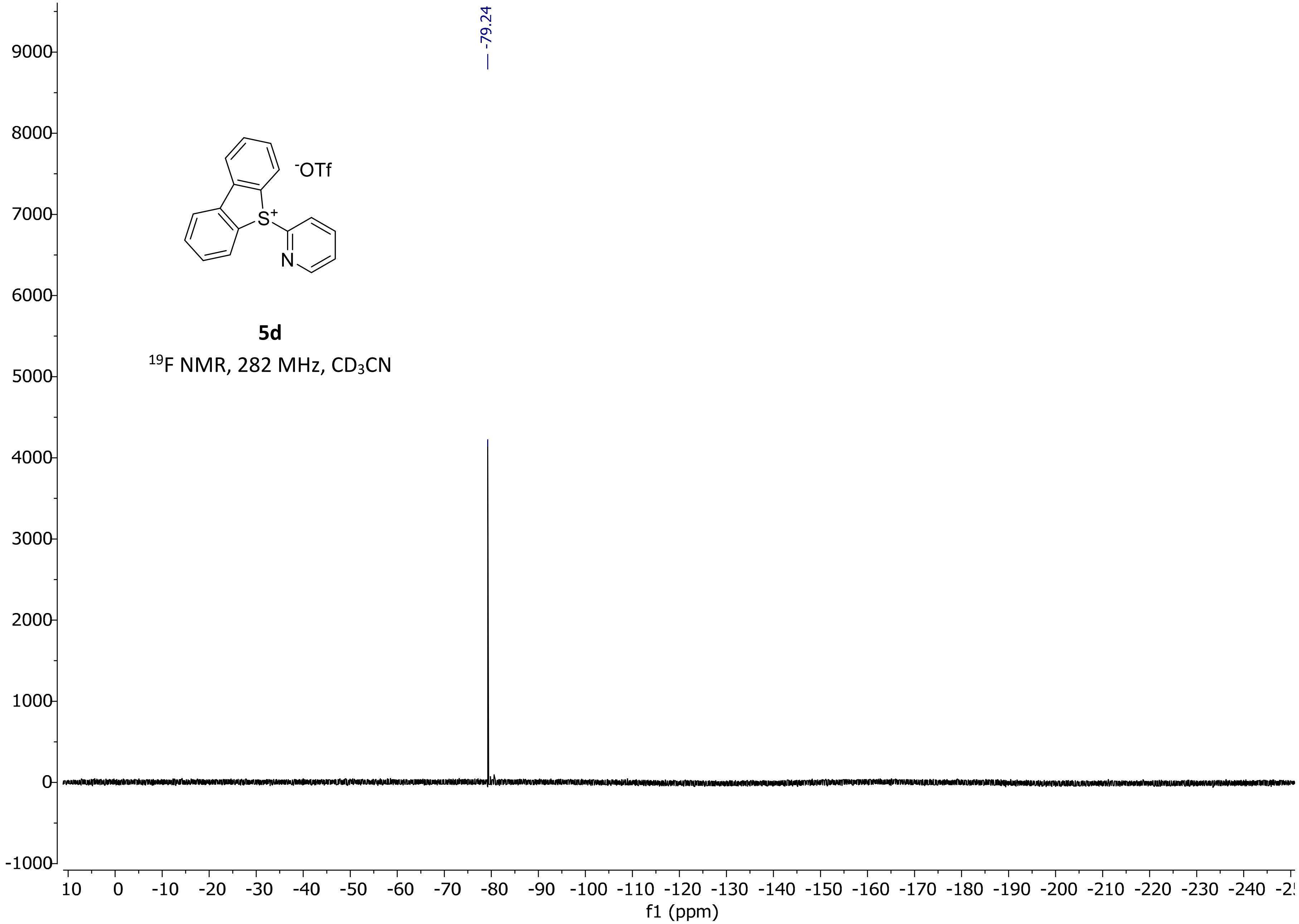
-79.24
-OTf
S+
N
5d
^{19}F NMR, 282 MHz, CD_3CN
f1 (ppm)

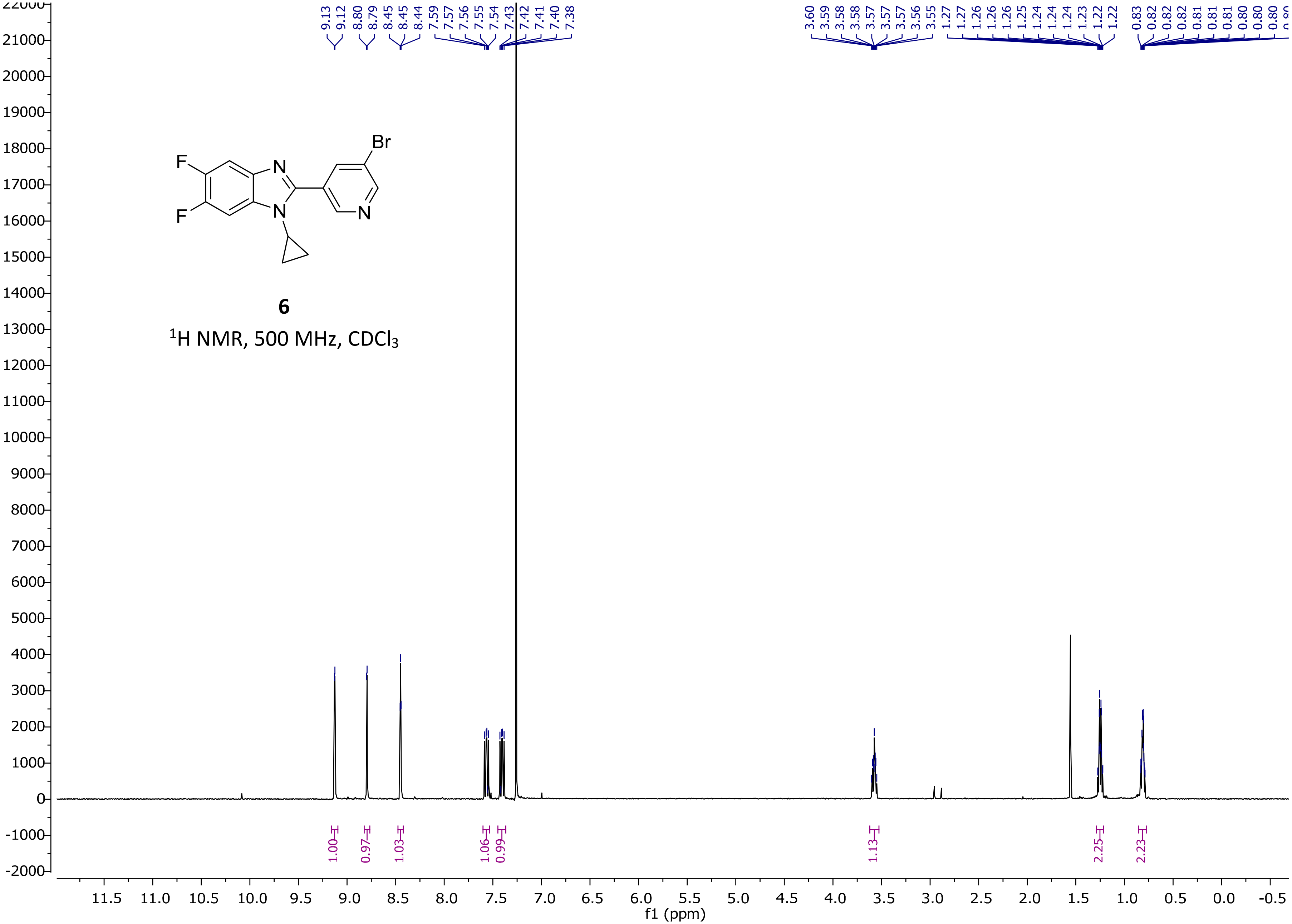
9.13
9.12
8.80
8.79
8.45
8.45
8.44
7.59
7.57
7.56
7.55
7.54
7.43
7.42
7.41
7.40
7.38
3.60
3.59
3.58
3.58
3.57
3.57
3.57
3.56
3.55
1.27
1.27
1.26
1.26
1.25
1.24
1.24
1.24
1.23
1.22
1.22
0.83
0.82
0.82
0.82
0.81
0.81
0.81
0.80
0.80
Br
F
F
N
N
N
6
^{1}H NMR, 500 MHz, $CDCl_3$
1.00
0.97
1.03
1.06
0.99
1.13
2.25
2.23
f1 (ppm)

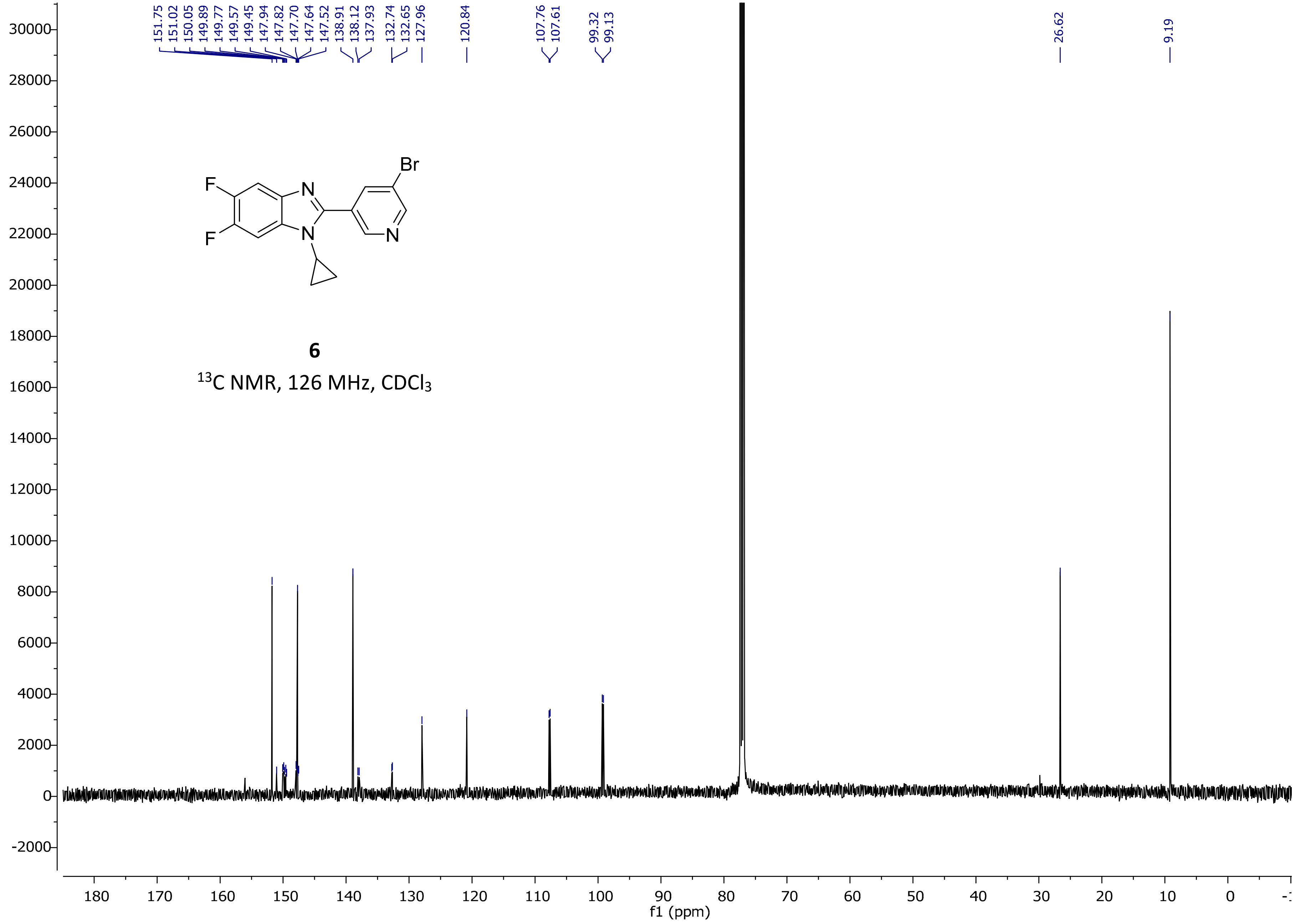
151.75
151.02
150.05
149.89
149.77
149.57
149.45
147.94
147.82
147.70
147.64
147.52
138.91
138.12
137.93
132.74
132.65
127.96
120.84
107.76
107.61
99.32
99.13
26.62
9.19
Br
F
F
N
N
N
6
^{13}C NMR, 126 MHz, $CDCl_3$
f1 (ppm)

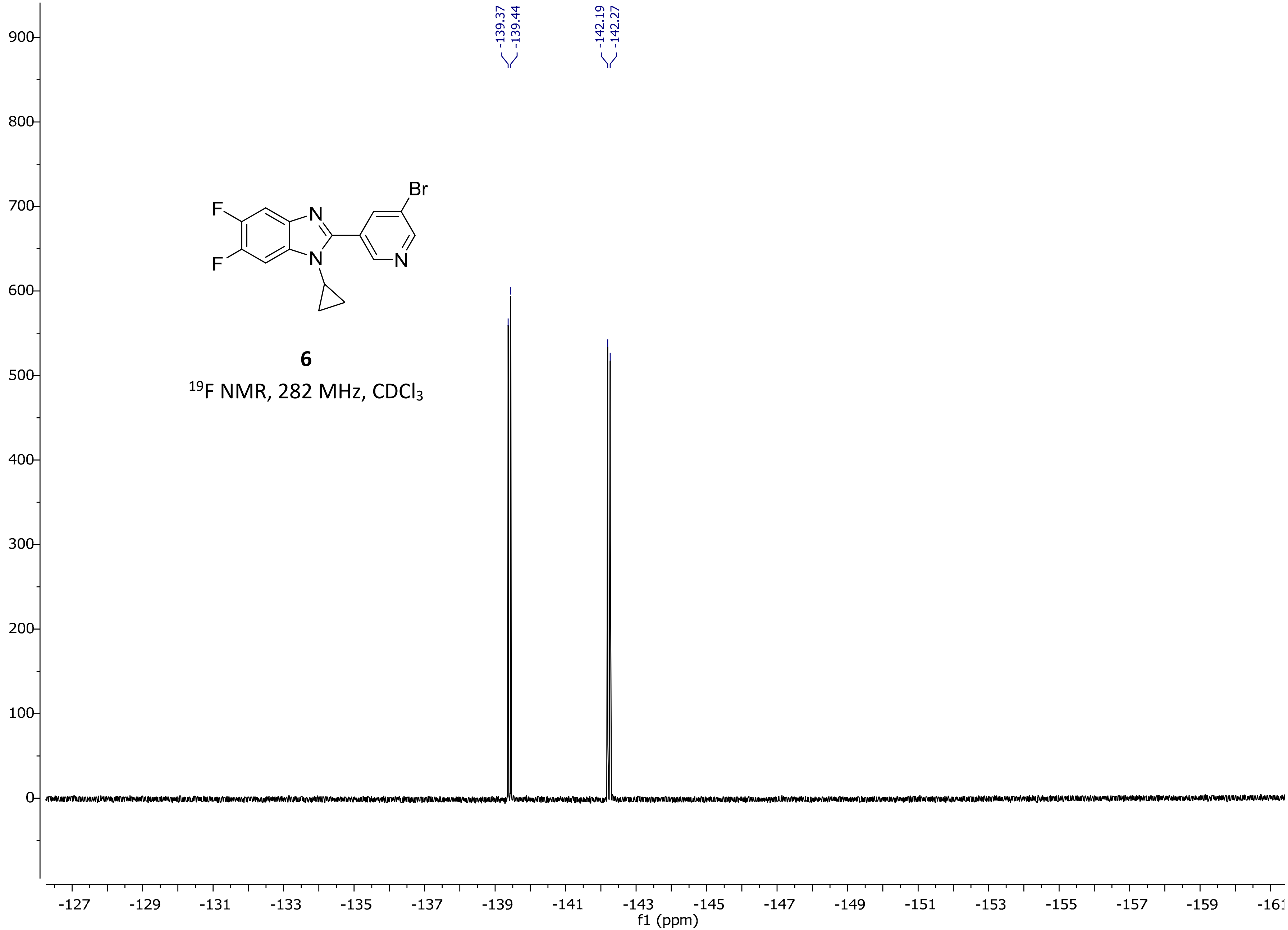

6

^{19}F NMR, 282 MHz, $CDCl_3$

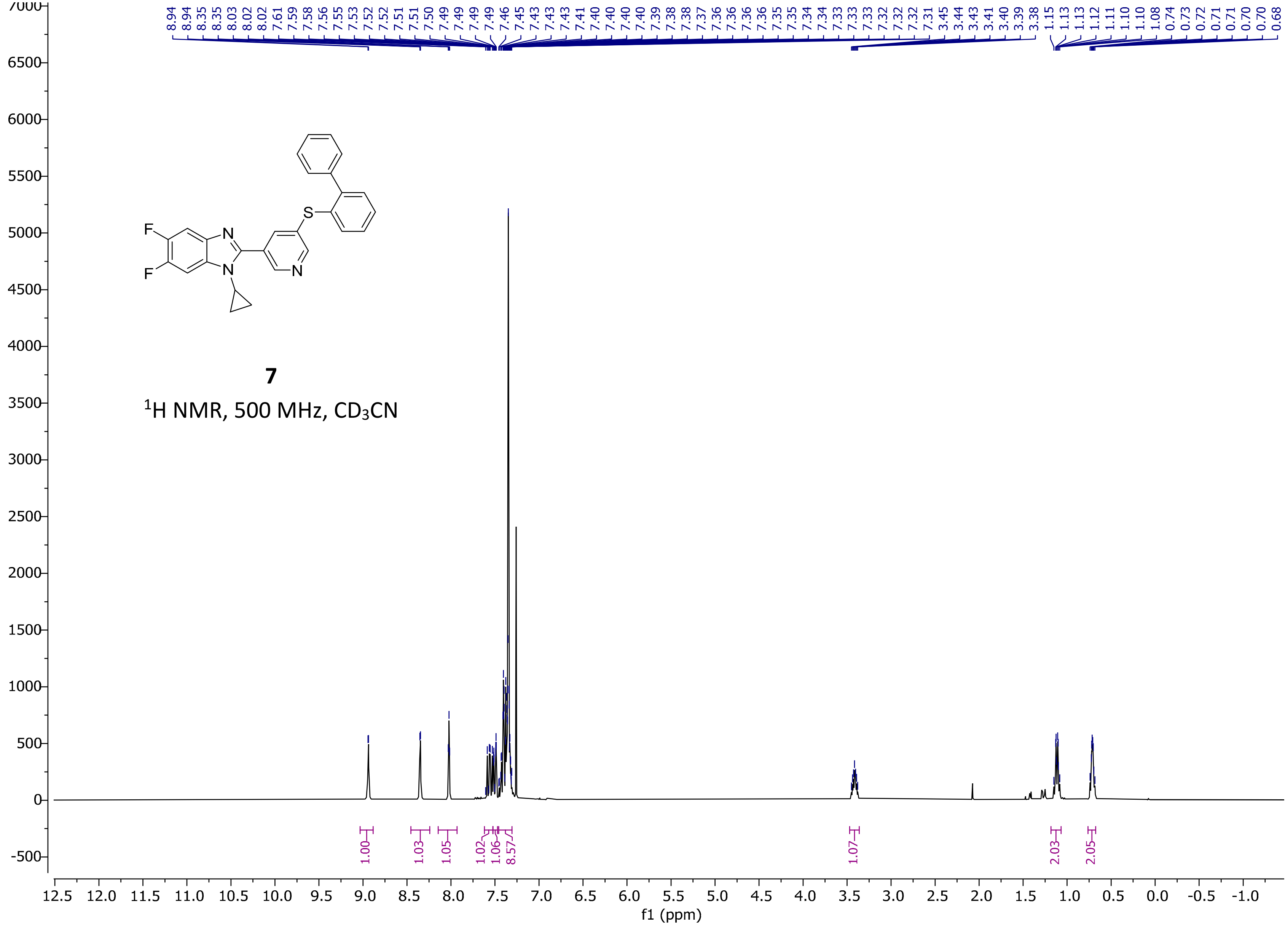
8.94 8.94 8.35 8.35 8.03 8.02 8.02 7.61 7.59 7.58 7.56 7.55 7.53 7.52 7.52 7.51 7.51 7.50 7.49 7.49 7.49 7.49 7.46 7.45 7.43 7.43 7.43 7.41 7.40 7.40 7.40 7.40 7.39 7.38 7.38 7.37 7.36 7.36 7.36 7.35 7.35 7.34 7.34 7.33 7.33 7.33 7.32 7.32 7.32 7.31 3.45 3.44 3.43 3.41 3.40 3.39 3.38 1.15 1.13 1.13 1.12 1.11 1.10 1.10 1.08 0.74 0.73 0.72 0.71 0.71 0.70 0.70 0.68
F
F
N
N
S
N
7
1H NMR, 500 MHz, CD3CN
1.00
1.03
1.05
1.02
1.06
8.57
1.07
2.03
2.05
f1 (ppm)

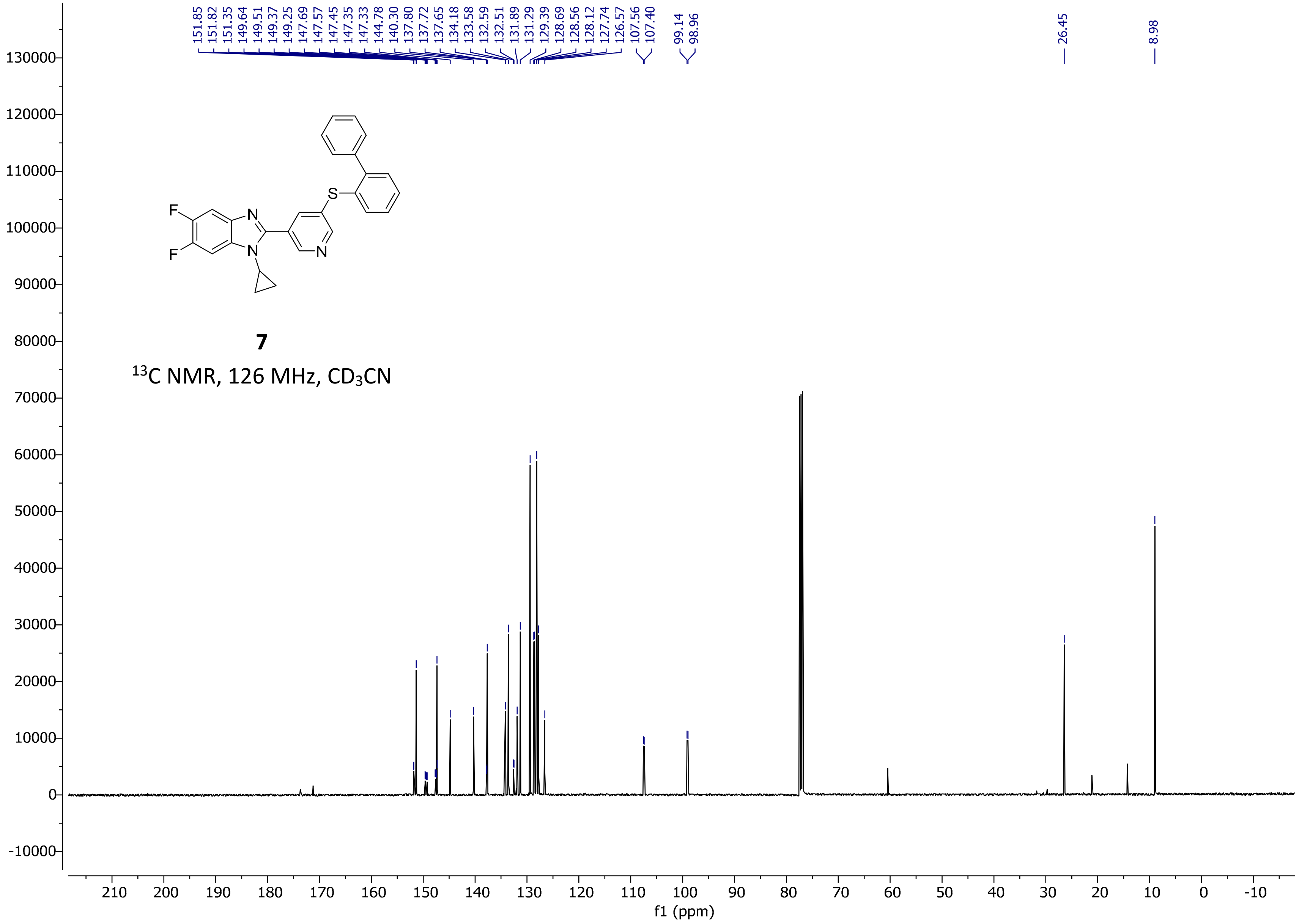
151.85
151.82
151.35
149.64
149.51
149.37
149.25
147.69
147.57
147.45
147.35
147.33
144.78
140.30
137.80
137.72
137.65
134.18
133.58
132.59
132.51
131.89
131.29
129.39
128.69
128.56
128.12
127.74
126.57
107.56
107.40
99.14
98.96
26.45
8.98
7
^{13}C NMR, 126 MHz, CD_3CN
f1 (ppm)

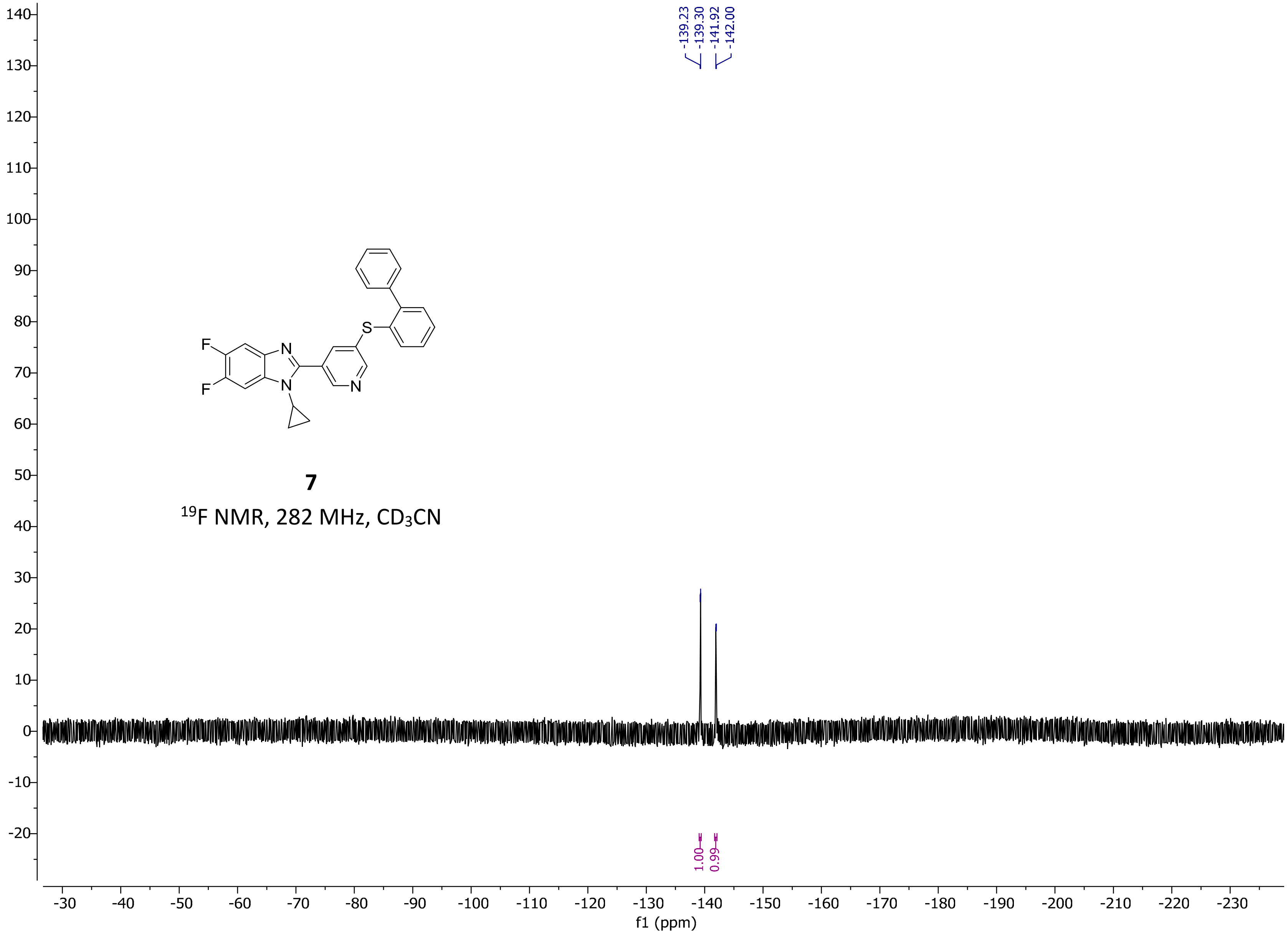
-139.23
-139.30
-141.92
-142.00
F
F
N
N
S
N
7
^{19}F NMR, 282 MHz, CD_3CN
1.00
0.99
f1 (ppm)

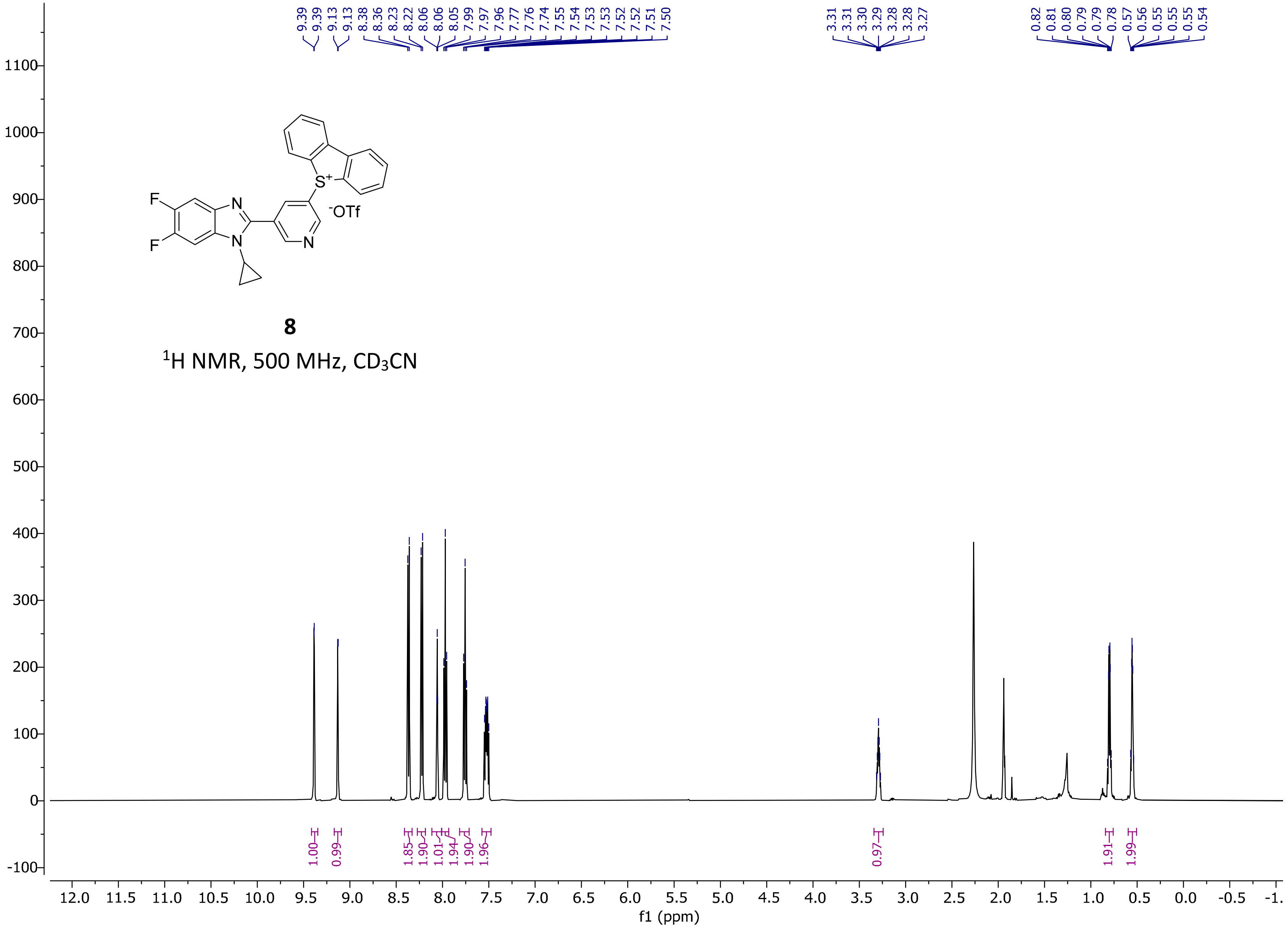

9.39
9.39
9.13
9.13
8.38
8.36
8.23
8.22
8.06
8.06
8.05
7.99
7.97
7.96
7.77
7.76
7.74
7.55
7.54
7.53
7.53
7.52
7.52
7.51
7.50
3.31
3.31
3.30
3.29
3.28
3.28
3.27
0.82
0.81
0.80
0.79
0.79
0.78
0.57
0.56
0.55
0.55
0.55
0.54
F
F
N
N
S+
N
-OTf
8
1H NMR, 500 MHz, CD3CN
1.00
0.99
1.85
1.90
1.01
1.94
1.90
1.96
0.97
1.91
1.99
f1 (ppm)

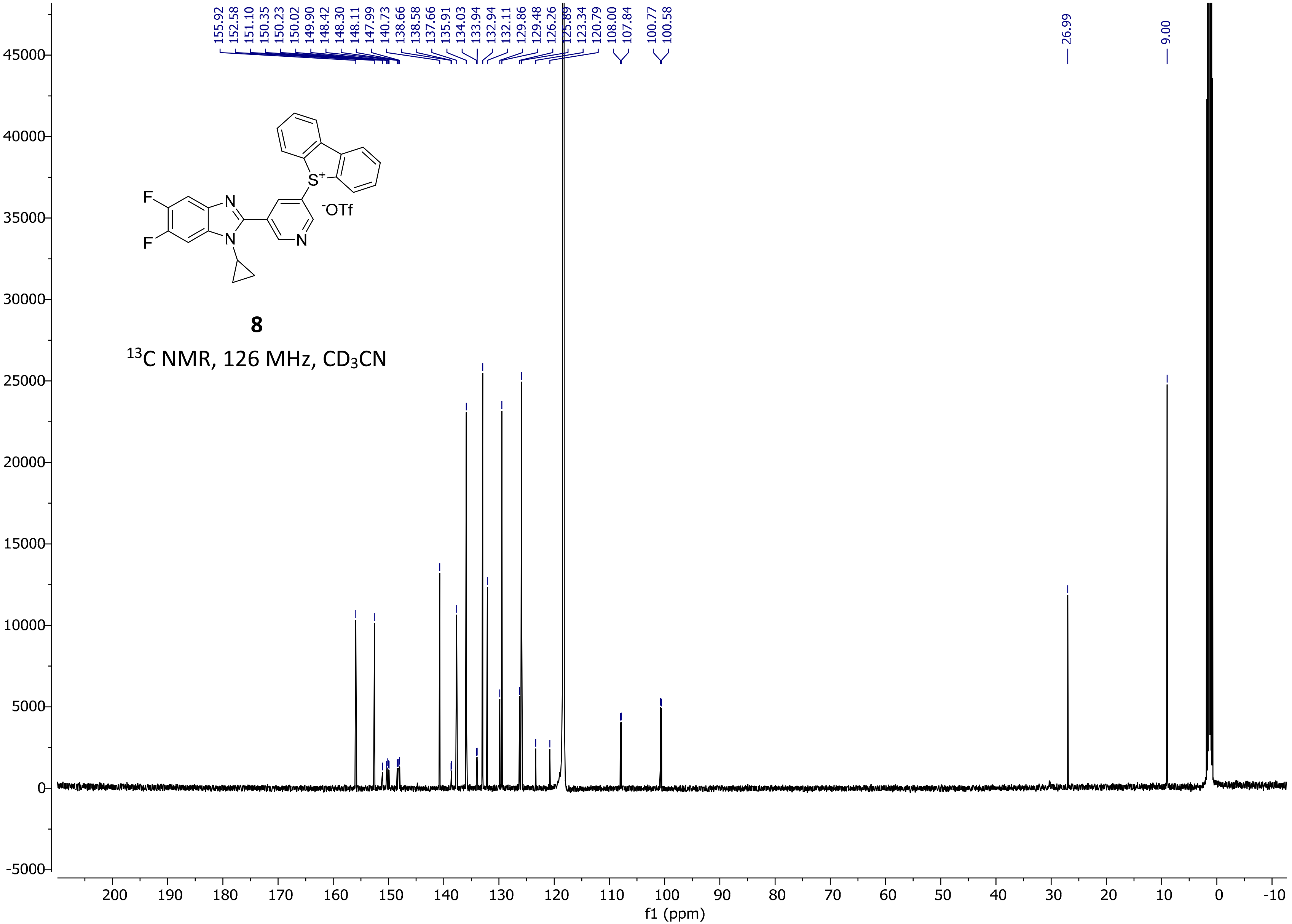
155.92
152.58
151.10
150.35
150.23
150.02
149.90
148.42
148.30
148.11
147.99
140.73
138.66
138.58
137.66
135.91
134.03
133.94
132.94
132.11
129.86
129.48
126.26
125.89
123.34
120.79
108.00
107.84
100.77
100.58
26.99
9.00
F
F
N
N
S+
-OTf
8
13C NMR, 126 MHz, CD3CN
45000
40000
35000
30000
25000
20000
15000
10000
5000
0
-5000
200 190 180 170 160 150 140 130 120 110 100 90 80 70 60 50 40 30 20 10 0 -10
f1 (ppm)

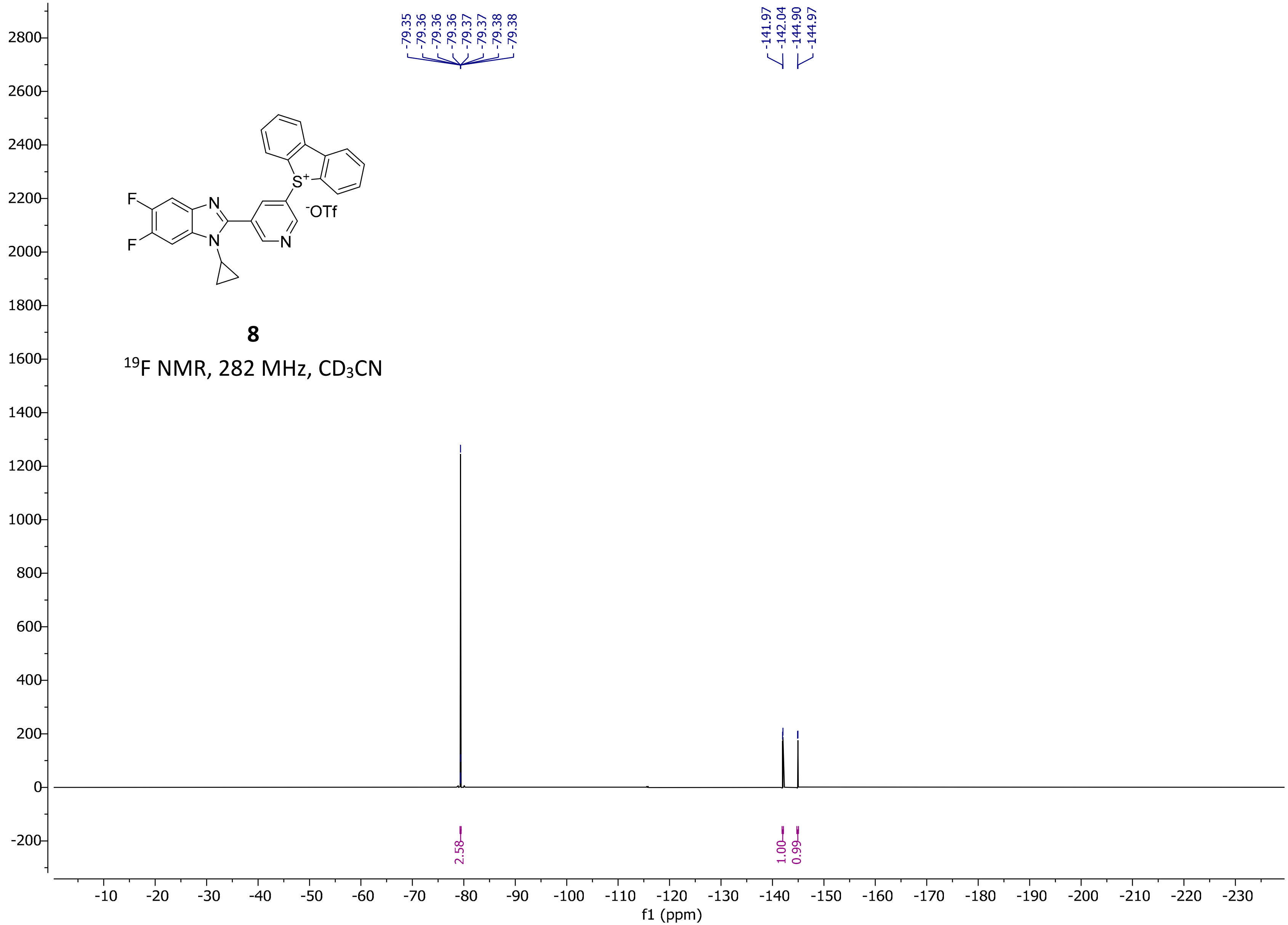
-79.35
-79.36
-79.36
-79.36
-79.37
-79.37
-79.38
-79.38
-141.97
-142.04
-144.90
-144.97
F
F
N
N
S+
-OTf
N
8
19F NMR, 282 MHz, CD3CN
2.58
1.00
0.99
f1 (ppm)

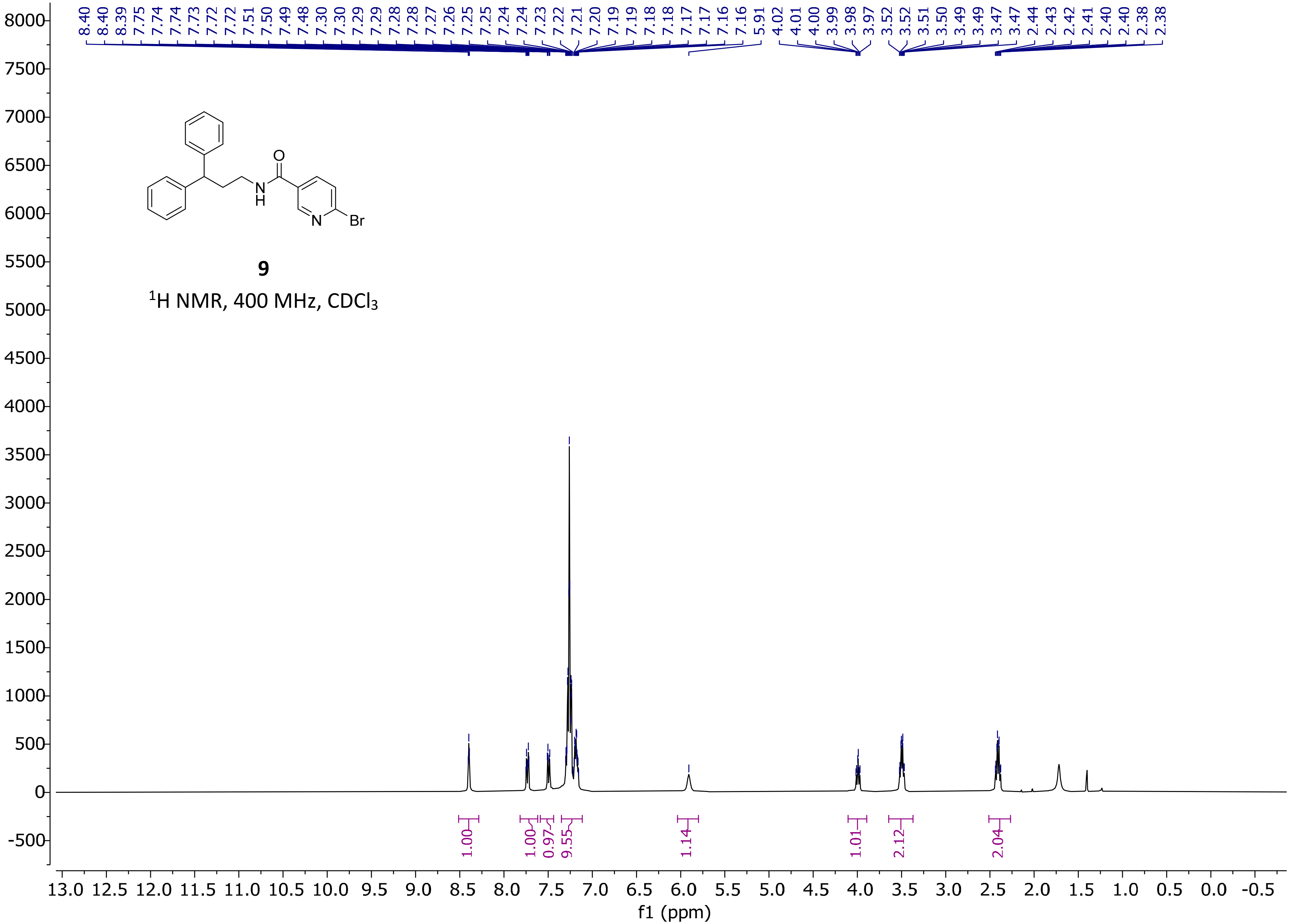
9
1H NMR, 400 MHz, CDCl3
f1 (ppm)

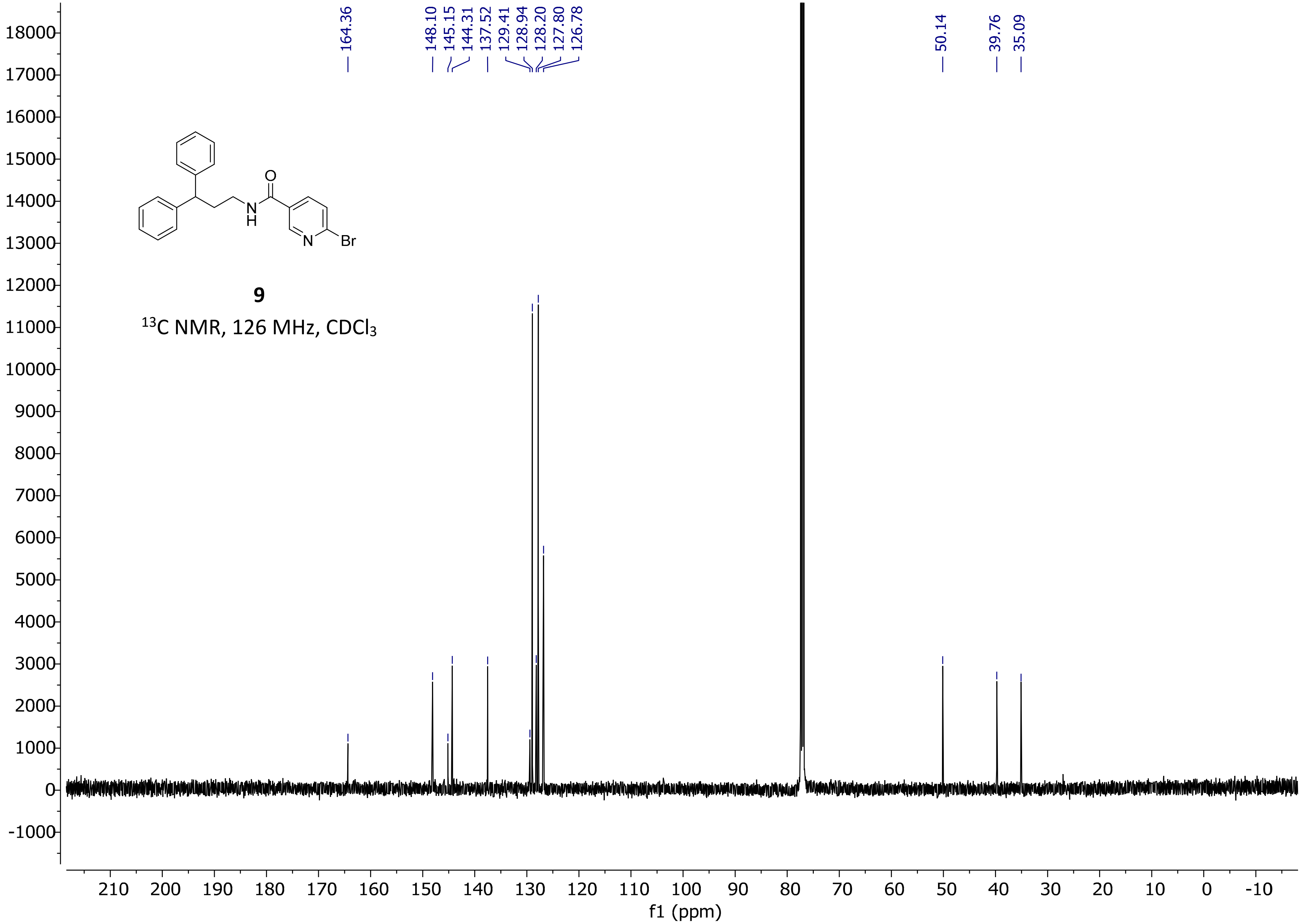
164.36
148.10
145.15
144.31
137.52
129.41
128.94
128.20
127.80
126.78
50.14
39.76
35.09
O
N
H
N
Br
9
^{13}C NMR, 126 MHz, $CDCl_3$
18000
17000
16000
15000
14000
13000
12000
11000
10000
9000
8000
7000
6000
5000
4000
3000
2000
1000
0
-1000
210 200 190 180 170 160 150 140 130 120 110 100 90 80 70 60 50 40 30 20 10 0 -10
f1 (ppm)

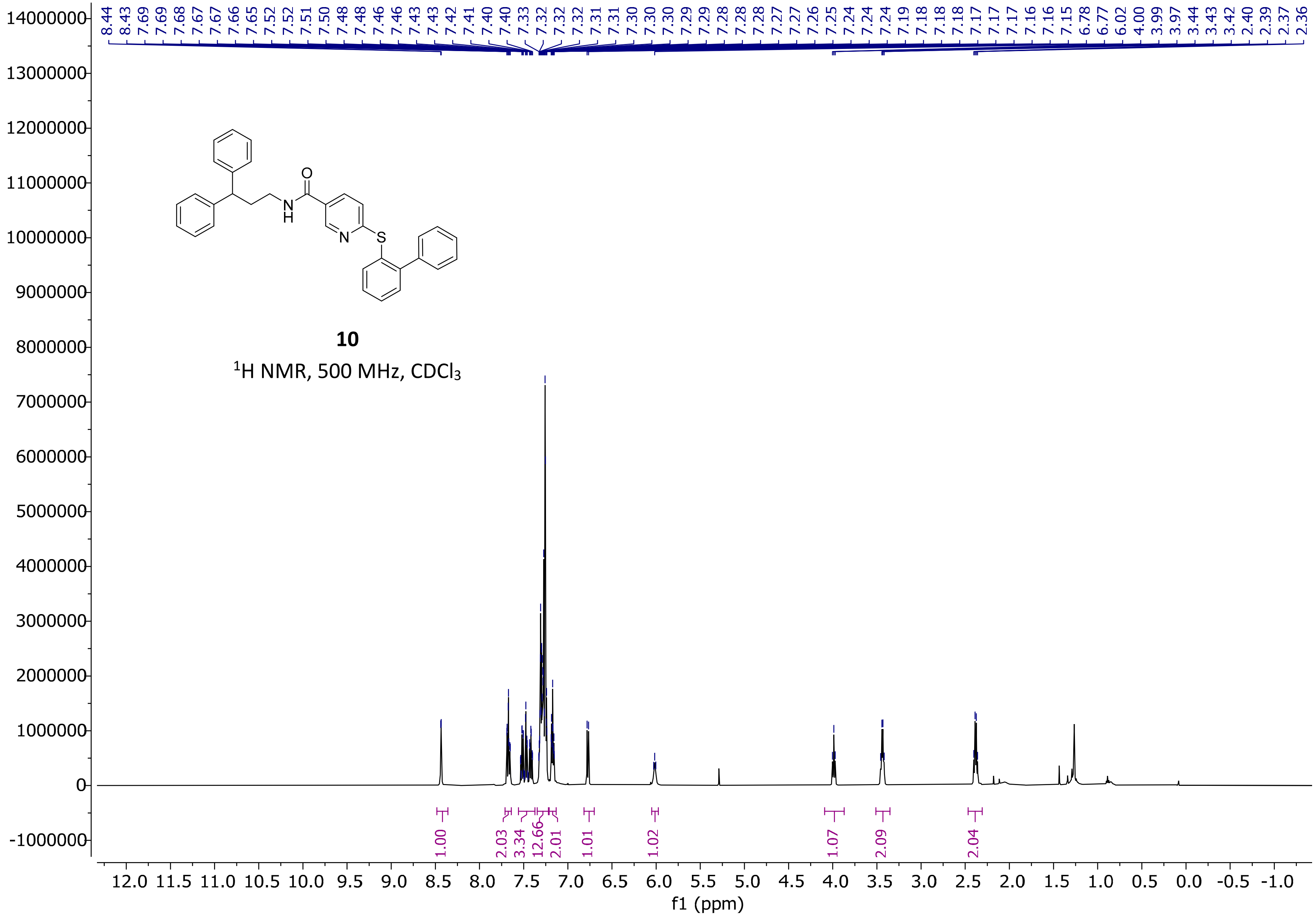

10

^{1}H NMR, 500 MHz, $CDCl_3$

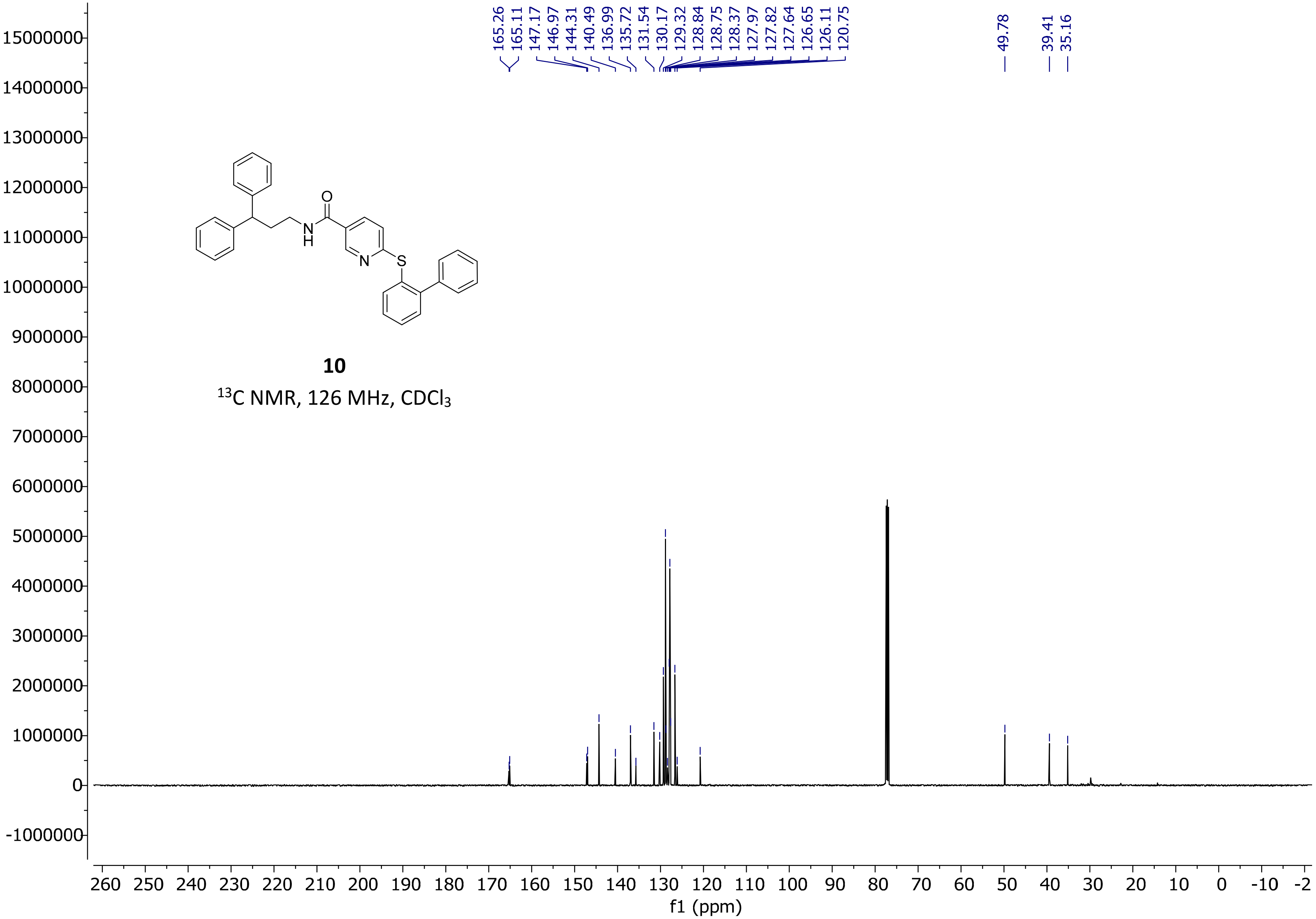

10

^{13}C NMR, 126 MHz, $CDCl_3$

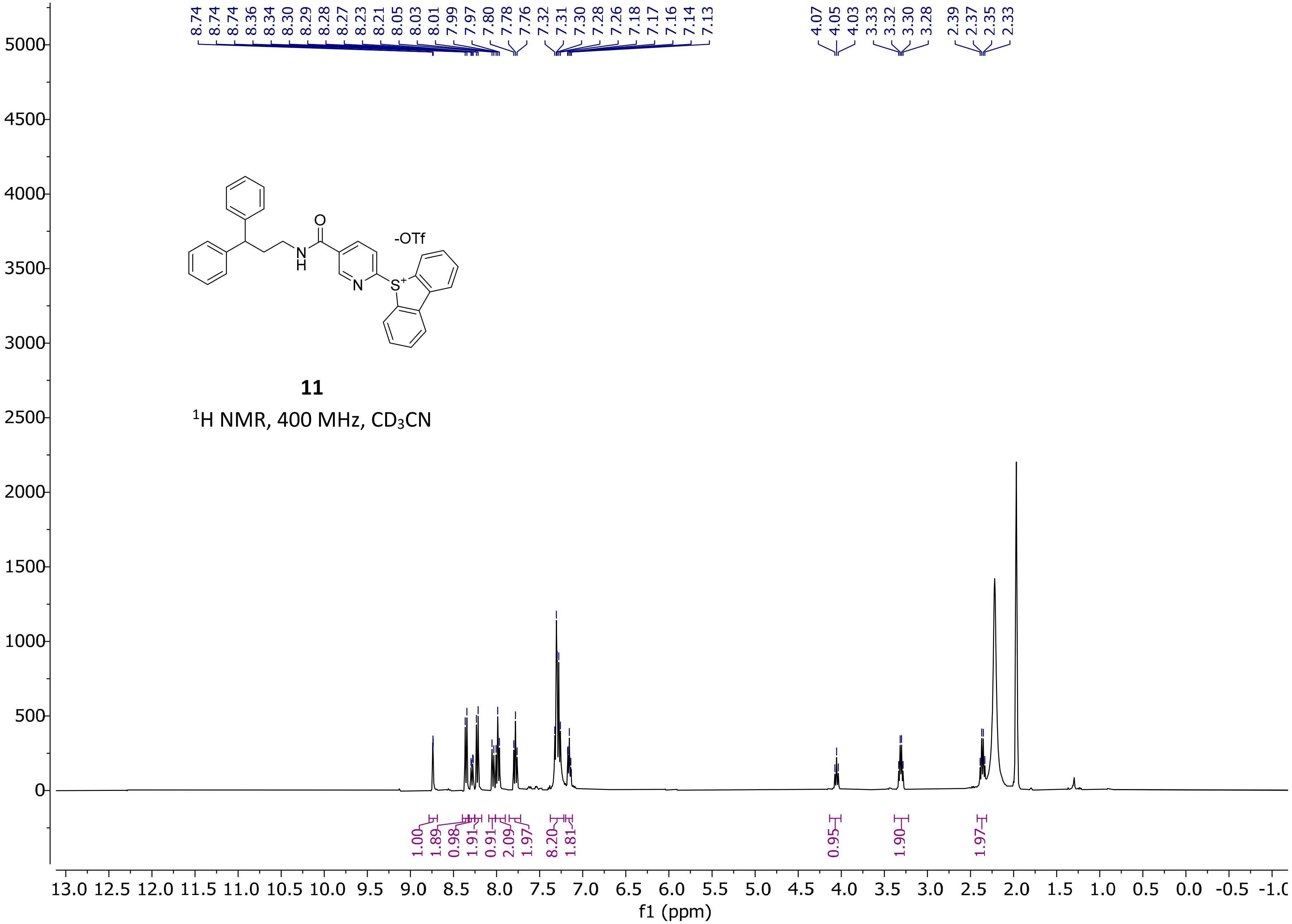

11

^{1}H NMR, 400 MHz, CD_3CN

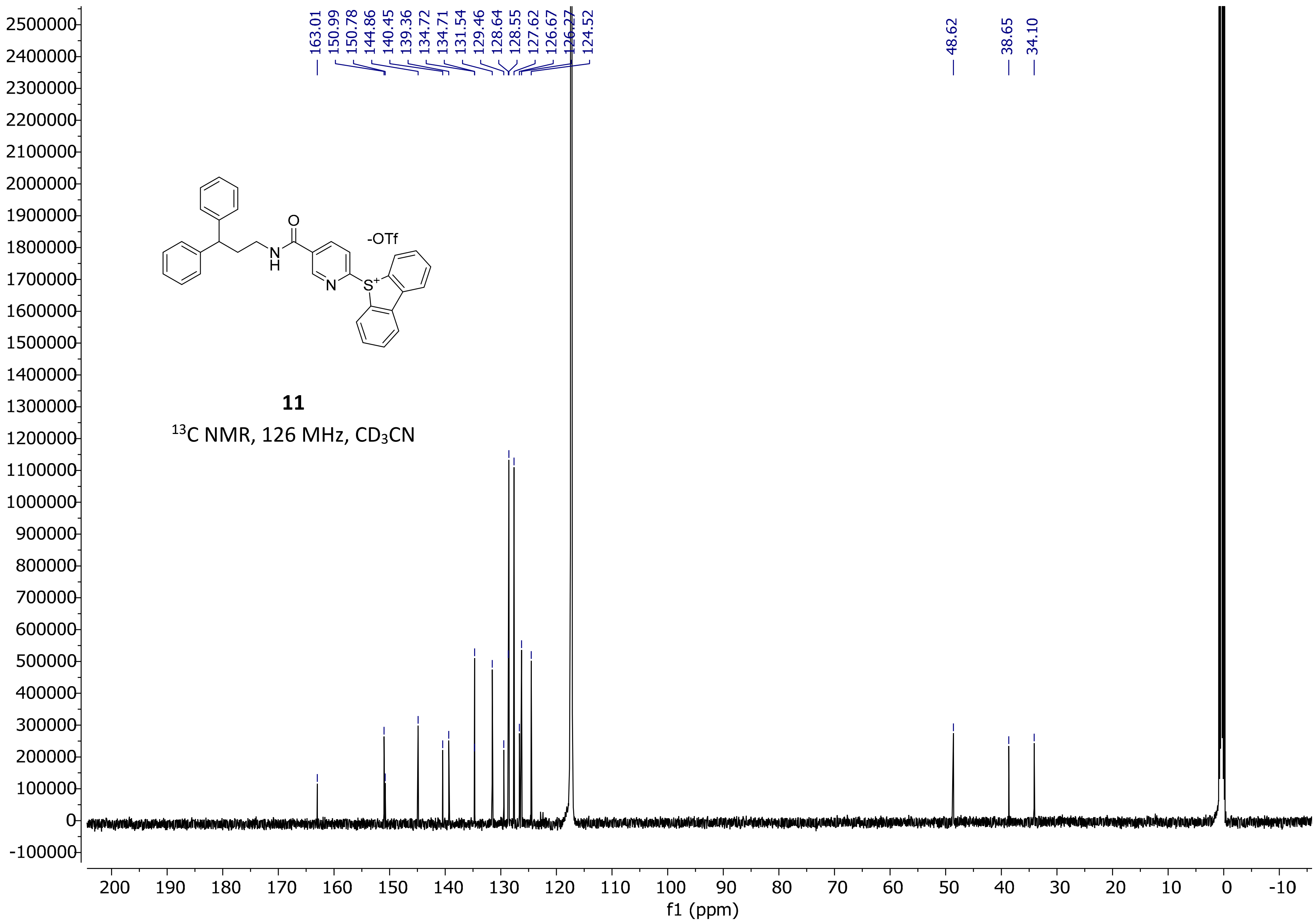
163.01
150.99
150.78
144.86
140.45
139.36
134.72
134.71
131.54
129.46
128.64
128.55
127.62
126.67
126.27
124.52
48.62
38.65
34.10
O
N
H
-OTf
N
S+
11
13C NMR, 126 MHz, CD3CN
f1 (ppm)

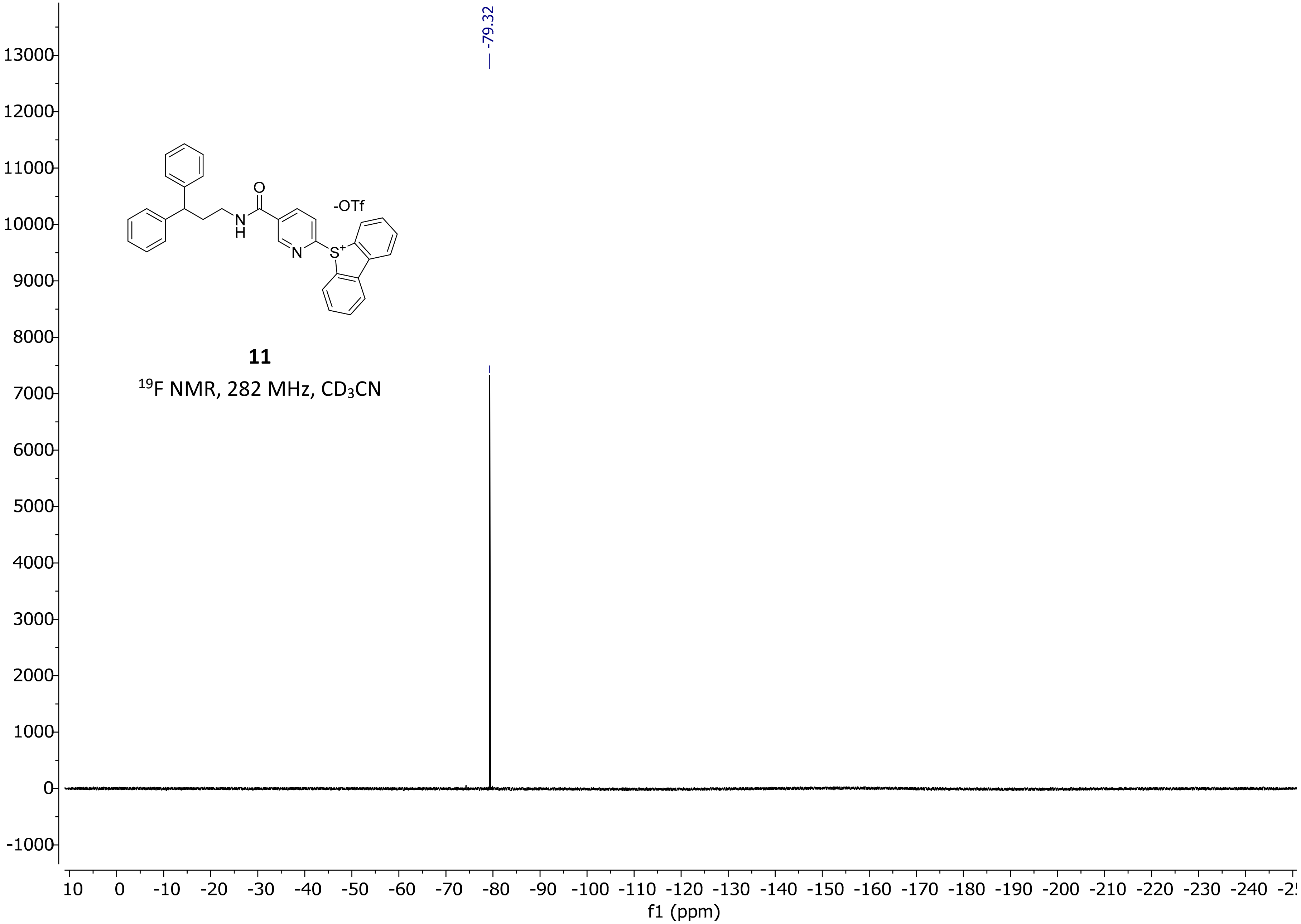

-79.32
-OTf
O
N
H
N
S+
11
^{19}F NMR, 282 MHz, CD_3CN
13000
12000
11000
10000
9000
8000
7000
6000
5000
4000
3000
2000
1000
0
-1000
10 0 -10 -20 -30 -40 -50 -60 -70 -80 -90 -100 -110 -120 -130 -140 -150 -160 -170 -180 -190 -200 -210 -220 -230 -240
f1 (ppm)

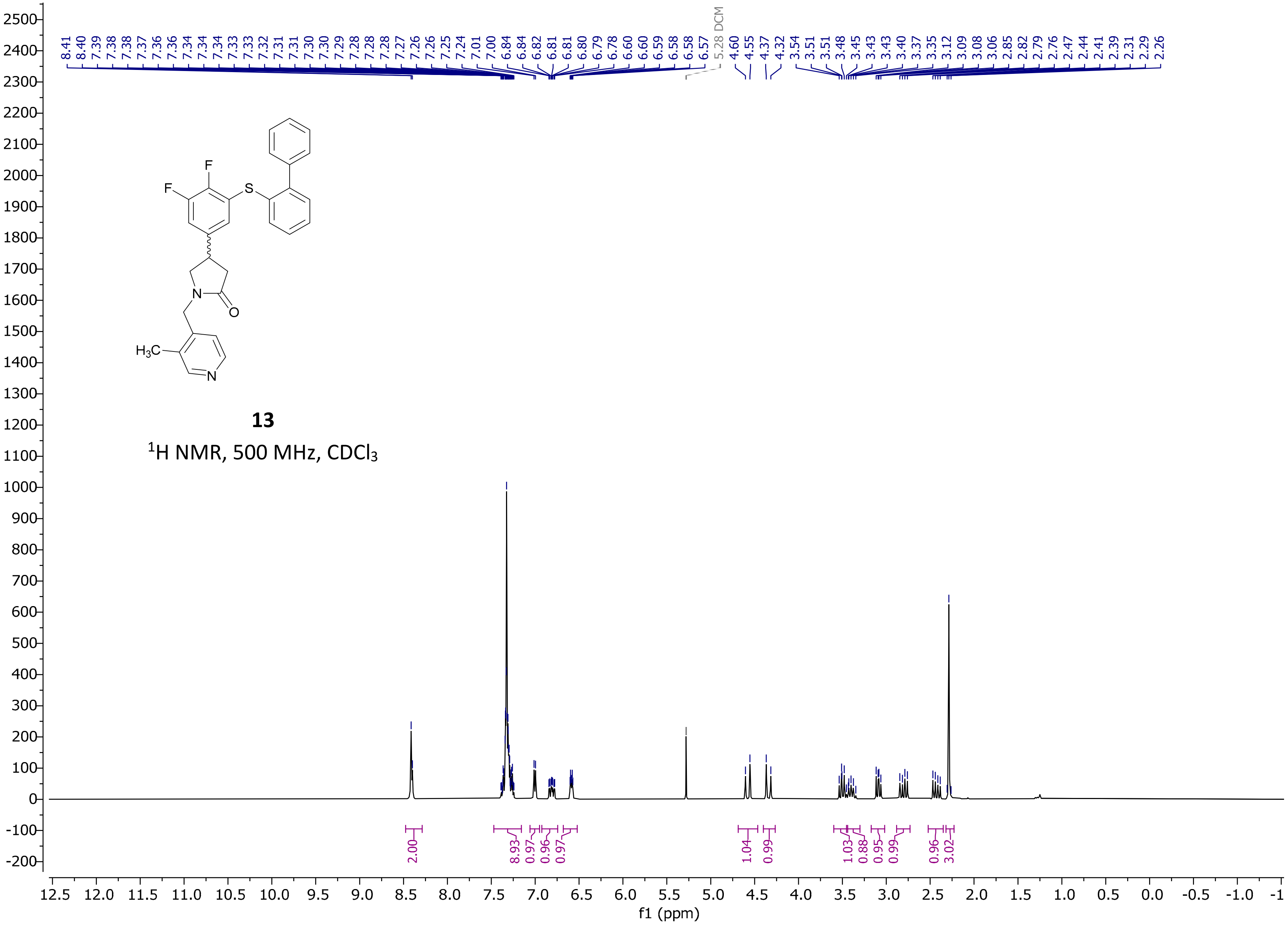

13

^1H NMR, 500 MHz, $CDCl_3$

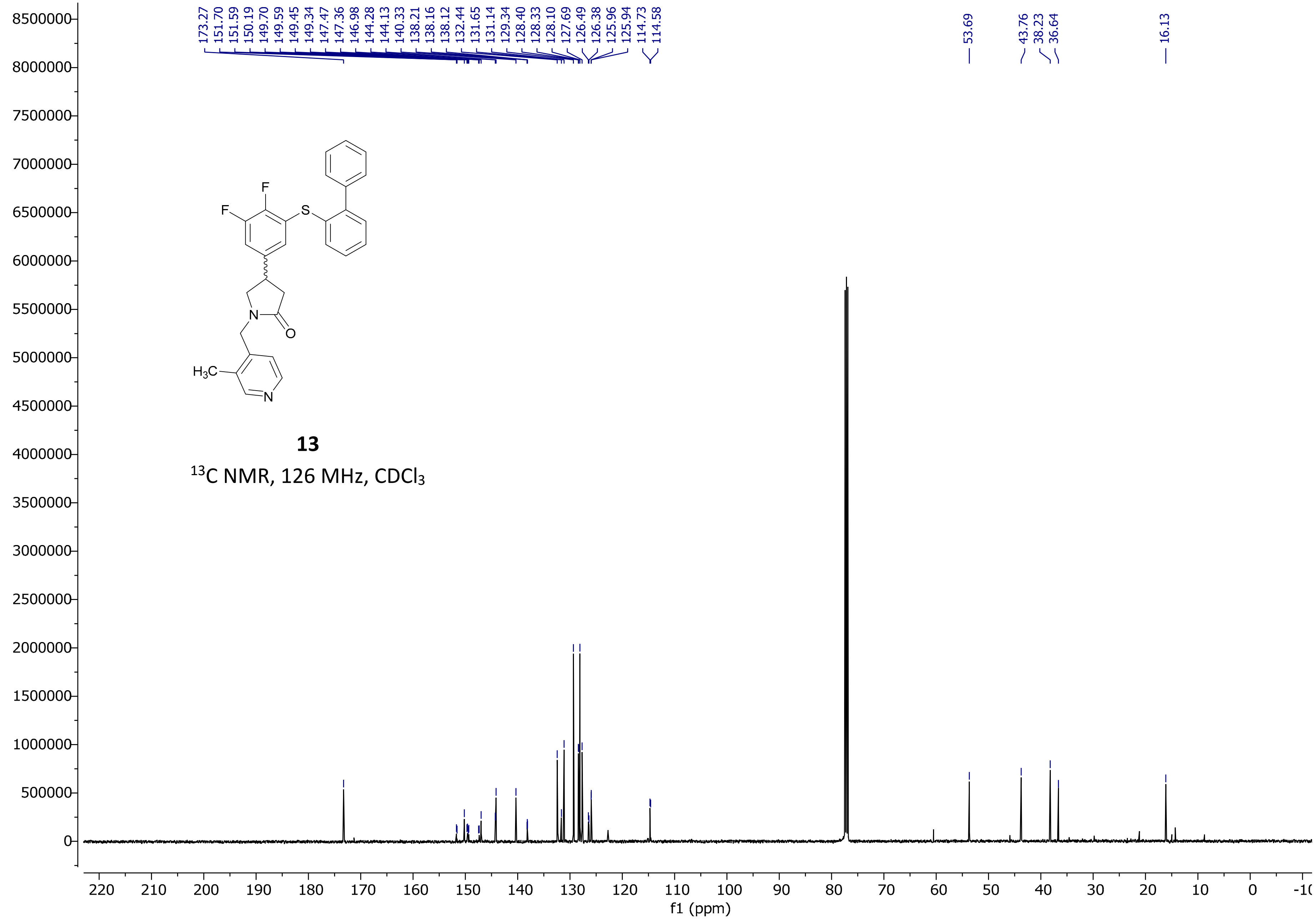

13

^{13}C NMR, 126 MHz, $CDCl_3$

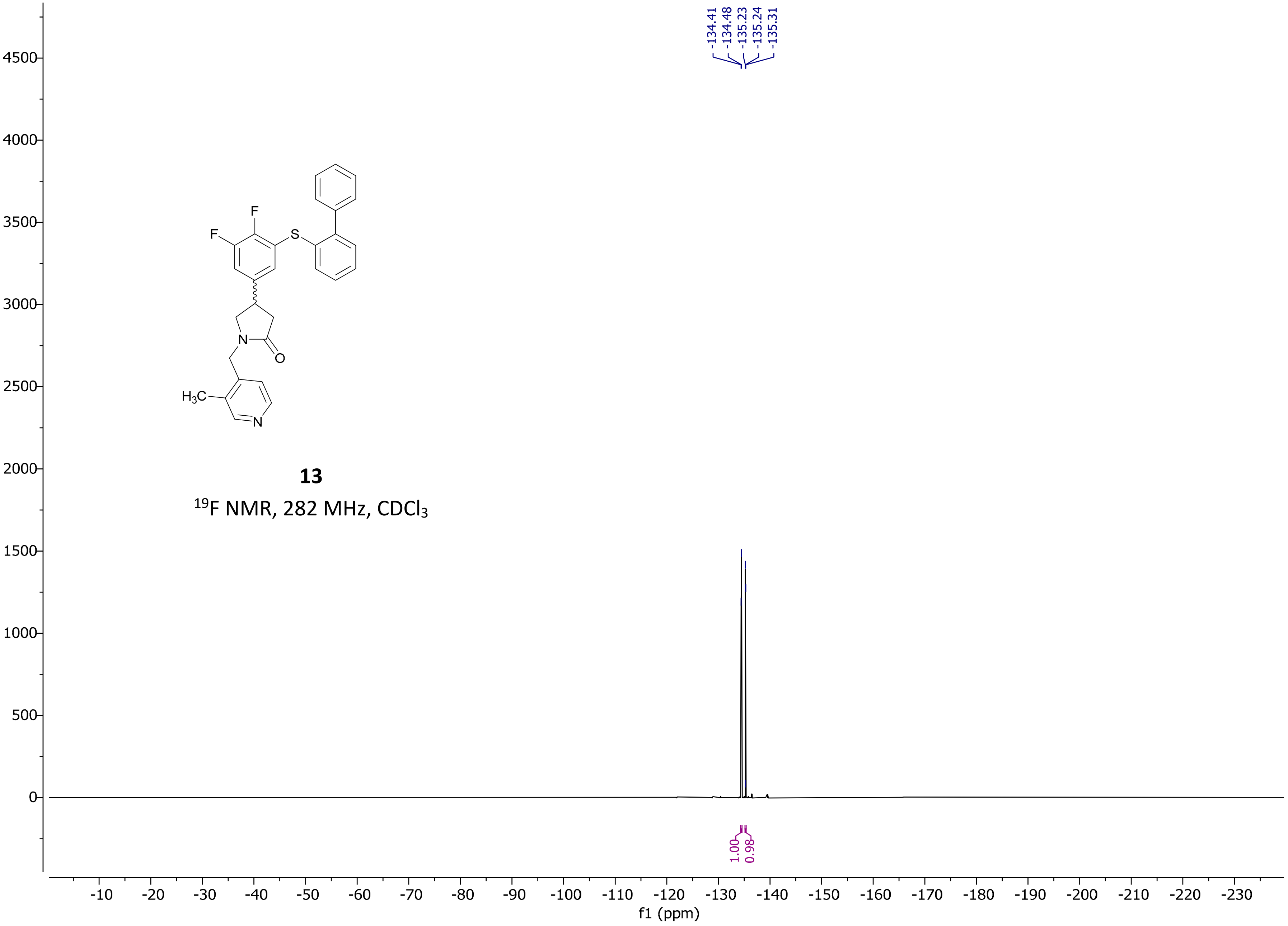

13

^{19}F NMR, 282 MHz, $CDCl_3$

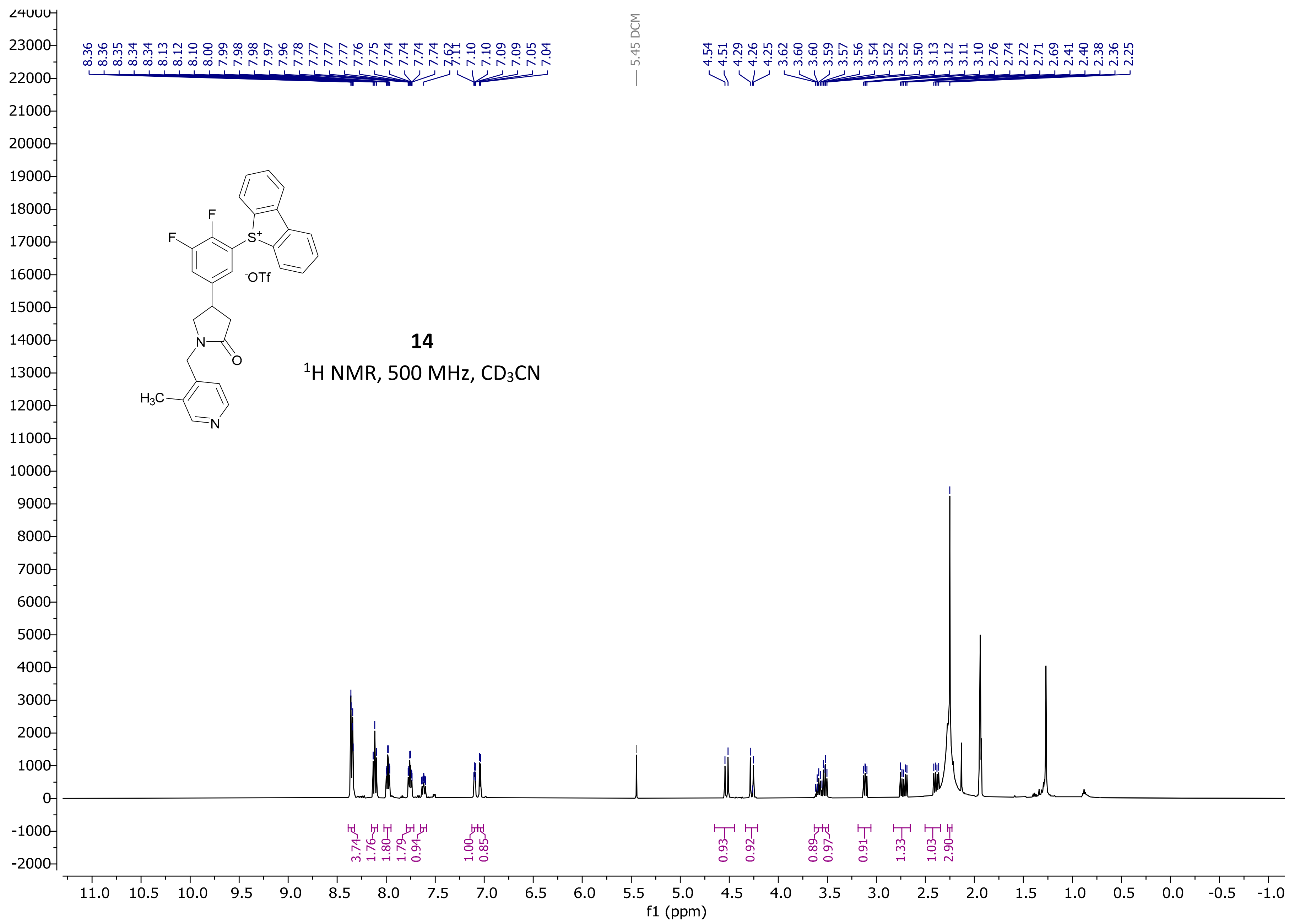
14
^{1}H NMR, 500 MHz, CD_3CN
F
F
S^+
$^-$OTf
N
O
H_3C
N
5.45 DCM
f1 (ppm)

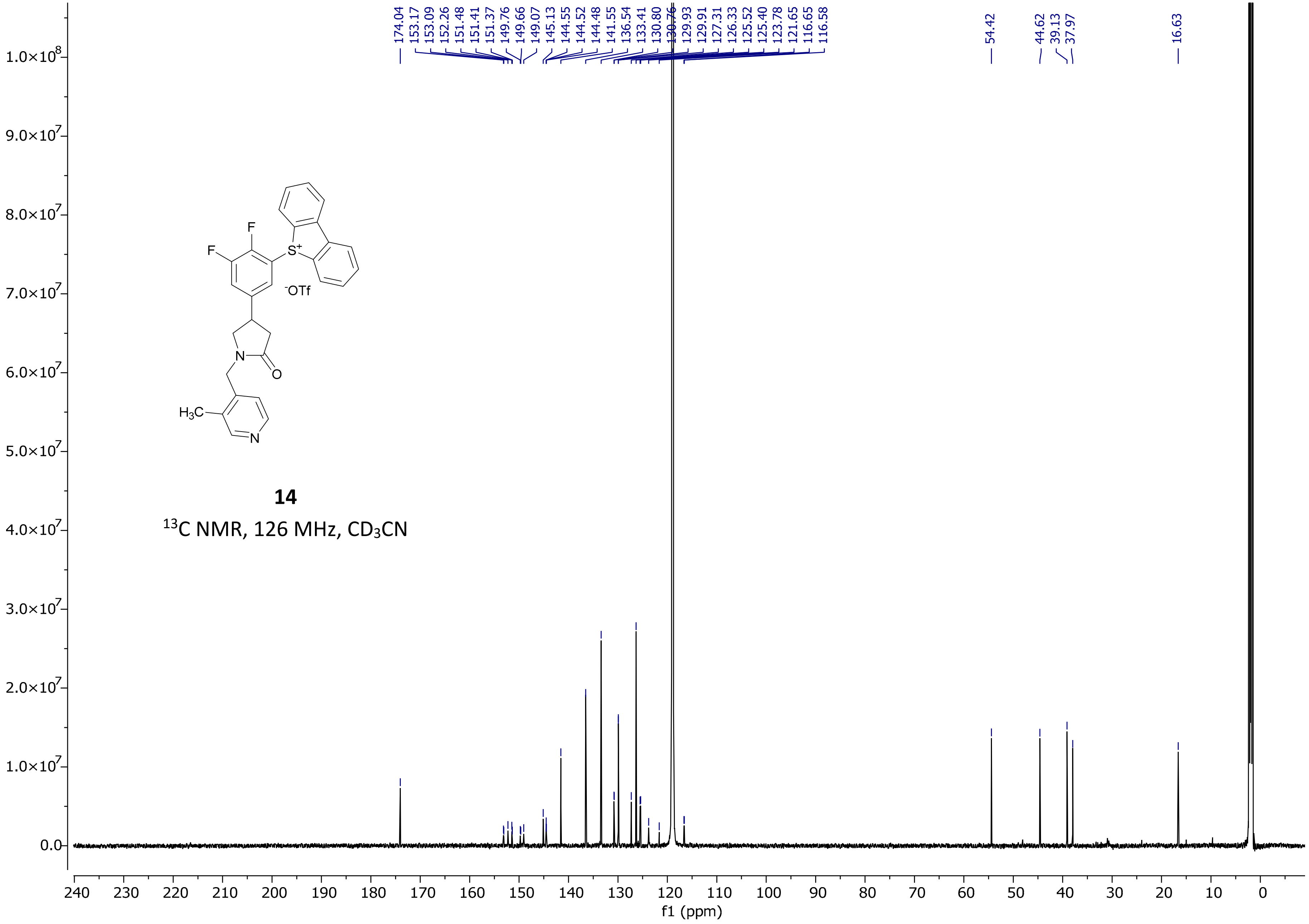
174.04
153.17
153.09
152.26
151.48
151.41
151.37
149.76
149.66
149.07
145.13
144.55
144.52
144.48
141.55
136.54
133.41
130.80
130.76
129.93
129.91
127.31
126.33
125.52
125.40
123.78
121.65
116.65
116.58
54.42
44.62
39.13
37.97
16.63
F
F
S+
-OTf
N
O
H3C
N
14
13C NMR, 126 MHz, CD3CN
f1 (ppm)

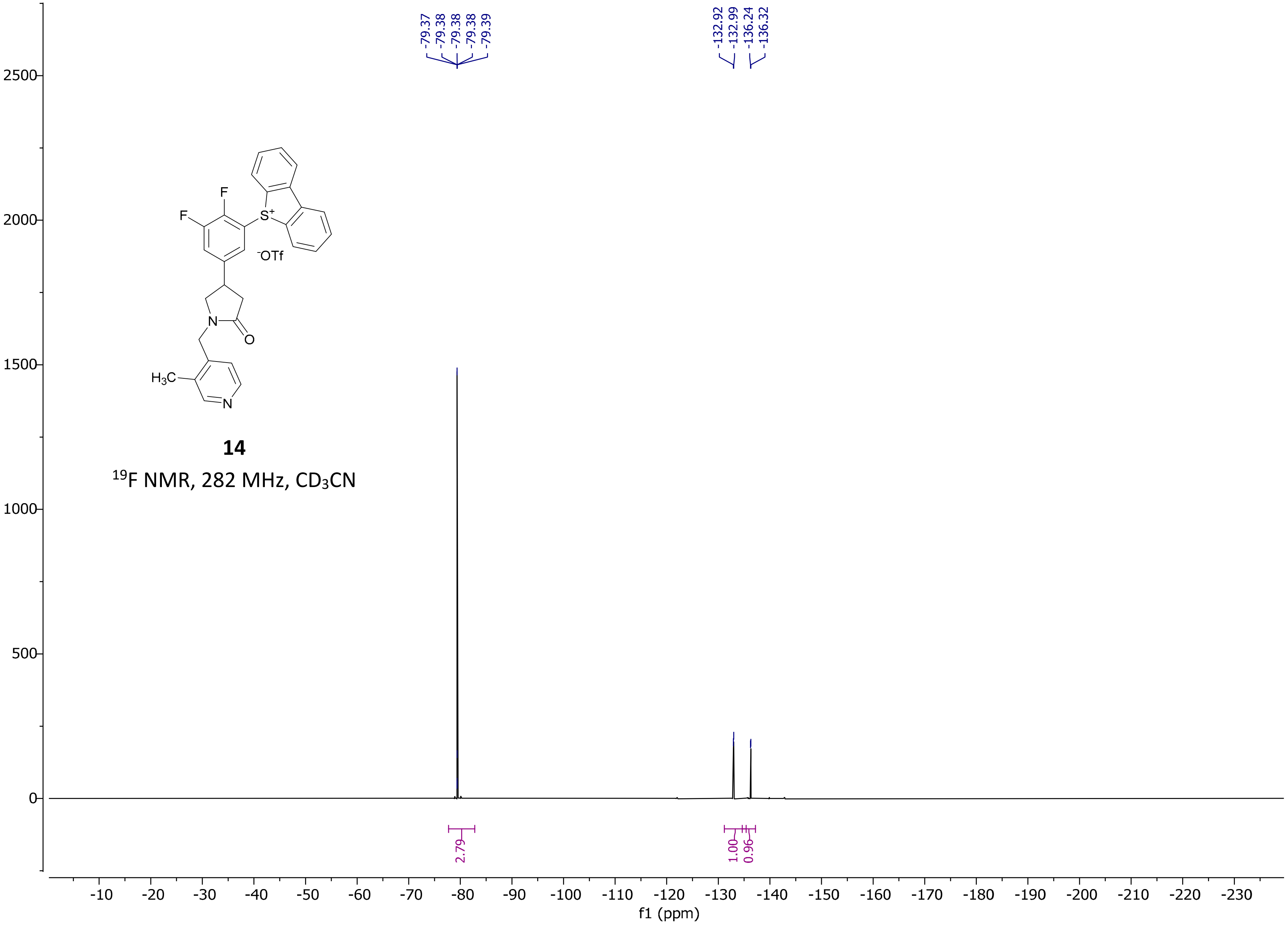

14

^{19}F NMR, 282 MHz, CD_3CN
